# Supporting clinical guidelines for opioid conversion to methadone and tapering to prevent withdrawal in critically ill children using physiology based pharmacokinetic modeling and simulation

**DOI:** 10.1371/journal.pone.0356917

**Published:** 2026-09-08

**Authors:** Marika A. de Hoop-Sommen, Sahar Alozai, Violette M. G. J. Gijsen, Tjitske M. van der Zanden, Henry T. Akinbi, Tomoyuki Mizuno, Alexander A. Vinks, Rick Greupink, Saskia N. de Wildt

**Affiliations:** 1 Department of Pharmacy, Pharmacology and Toxicology, Radboud university medical center, Nijmegen, The Netherlands; 2 Royal Dutch Pharmacy Association (KNMP), Medicines Information Centre (GIC), The Hague, The Netherlands; 3 Dutch Knowledge Centre Pharmacotherapy for Children, Rotterdam, The Netherlands; 4 Division of Neonatology, Perinatal Institute, Cincinnati Children's Hospital Medical Center, Cincinnati, Ohio, United States of America; 5 Department of Pediatrics, University of Cincinnati College of Medicine, Cincinnati, Ohio, United States of America; 6 Division of Translational and Clinical Pharmacology, Cincinnati Children's Hospital Medical Center, Cincinnati, Ohio, United States of America; 7 Department of Neonatal and Pediatric Intensive Care, Division of Pediatric Intensive care, Erasmus MC-Sophia Children’s Hospital, Rotterdam, The Netherlands; Harvard Medical School, UNITED STATES OF AMERICA

## Abstract

Long-term opioid use is common in critically ill children and can cause withdrawal symptoms at discontinuation. To avoid withdrawal, short-acting opioids are usually converted into a long-acting drug, such as methadone, which is then carefully tapered. However, the exact conversion ratio and the optimal tapering protocol are unknown. This study aimed to design optimized conversion and tapering schedules of morphine and fentanyl to methadone for pediatric intensive care unit patients using physiologically-based pharmacokinetic (PBPK) modeling and expert panel decision-making. PBPK models of fentanyl, morphine, and methadone were verified with published pharmacokinetic data and opioid conversion ratios and relevant tapering protocols identified through literature review. Then, plasma concentration-time curves of opioid infusions (1 µg/kg/h fentanyl, 10 µg/kg/h morphine), several opioid-methadone conversion ratios, and different methadone tapering protocols were simulated. Predicted methadone plasma concentrations were compared to minimal therapeutic concentrations (i.e., 1 ng/mL for fentanyl, 4 ng/mL for morphine, and 60 ng/mL for methadone). These simulations were reviewed by the expert panel consisting of pediatric intensivists and hospital pharmacists to establish recommendations. Simulations showed that a conversion ratio of 1:10 for fentanyl and 1:1 for morphine resulted in therapeutic methadone concentrations. Most previously established tapering recommendations resulted in equal maximum methadone concentrations, except for the low-risk tapering schedules of two of the three protocols. The robustness of these findings is reinforced by both a target attainment analysis and a sensitivity analysis employing the therapeutic target of methadone. The panel established consensus-based Dutch recommendations for conversion and tapering. These findings demonstrate that PBPK models can support dosing decisions by simulating clinical scenarios. The resulting recommendations have been implemented in the Dutch Pediatric Formulary, ensuring that the model-informed recommendations are directly applied in clinical practice.

## Introduction

Long-term opioid use is common in critically ill children in the pediatric intensive care unit (PICU) and can cause iatrogenic withdrawal syndrome (IWS) if not tapered carefully. The most predictive risk factors for IWS are the cumulative opioid dose, peak dose, and duration of opioid treatment [1]. Patients are often classified into withdrawal risk categories based on these predictive factors; the longer the opioid use and the higher the (cumulative) dose, the longer the tapering period [2,3].

While the evidence for the relationship between withdrawal symptoms and opioid dose and duration is compelling in the PICU population, the evidence for a specific tapering protocol or the exact thresholds for withdrawal risk categories is not. Nevertheless, clinical consensus supports the conversion of short-acting opioids to long-acting drugs [1]. Methadone is considered an ideal drug due to its favorable oral bioavailability and long half-life, which diminishes the chance of withdrawal symptoms [4]. In addition, the long half-life allows for simplified dosing regimens, making the drug suitable for use in the outpatient setting when switching from intravenous (IV) to oral (PO) administration. However, it is unknown how opioids are ideally converted to methadone.

Although the implementation of tapering protocols in itself has been shown to reduce adverse outcomes, including duration of PICU length of stay, evidence is insufficient to recommend any particular tapering strategy [1]. This may lead to inadequate tapering processes, which can further increase the complexity of treatment and affect patient well-being. Several conversion ratios and tapering strategies have been suggested, but the underlying evidence to support these approaches is unclear.

In the absence of high level evidence from clinical trials, pharmacokinetic (PK) modeling may be helpful to simulate several scenarios to support dosing decisions [5]. This is a method increasingly used not only in drug development but also in clinical care [6]. An attractive modeling tool, especially when existing PK data are limited, is physiologically-based pharmacokinetic (PBPK) modeling and simulation [7]. A PBPK model is a mathematical model of human physiology, consisting of multiple compartments representing human organs and tissues connected by blood flow. These models have evolved to also include pediatric data on organ size and enzyme and transporter ontogeny, making them ideally suited to predict plasma concentrations in children. The physiological characteristics are combined with drug-specific data to predict the absorption, distribution, metabolism, and excretion of a drug, and to simulate doses based on target exposures (i.e., desired therapeutic blood concentrations [7]. Simulated scenarios can then be presented to an expert panel to decide by consensus on the preferable conversion and tapering approaches, in the absence of clinical trials supporting optimized tapering protocols [8].

Hence, the aim of our study is to design optimized conversion and tapering schedules of morphine and fentanyl to methadone for pediatric ICU patients using PBPK modeling and expert panel decision-making, to be implemented in the Dutch Pediatric Formulary (DPF).

## Materials and methods

To enable decision making on optimal conversion and tapering of opioids, we reviewed the literature on conversion and tapering and compared expected drug exposures and therapeutic target using modeling and simulation. These simulations were used by an expert panel to establish national opioid conversion and tapering recommendations.

### Review of literature

#### Identification and selection of conversion ratios and tapering protocols.

To identify the available conversion ratios and tapering schedules, we performed a systematic literature search in May 2026, using PubMed, Embase and CINAHL, to find articles describing fentanyl or morphine conversion in critically ill children at the PICU. The search terms and the in- and exclusion criteria can be found in the Supporting Information (S1 File, section 1.1 and 1.2). Included studies were summarized in a table (S1 File, section 1.3), describing all relevant study characteristics. In addition, we compared the literature results with the most commonly used Dutch guideline [9]. A selection was then made from the conversion ratios found in literature based on the spread in the ratios and whether the ratio was used in multiple studies. For the selection of tapering protocols, protocols were chosen that distinguished between withdrawal risk categories.

#### Identification of therapeutic targets.

The overall concept of our approach is based on ‘exposure matching’. When converting one opioid to another, it was our goal to keep the combined opioid concentrations stable at an equipotent concentration, followed by a slow decrease in methadone concentration to prevent withdrawal symptoms and to avoid oversedation. We therefore also searched the literature for therapeutic targets of fentanyl, morphine, and methadone (S1 File, section 2.2).

### Selection and verification of the PBPK models

For our simulations, we used the PBPK software platform Simcyp™ (version 23, Certara, Sheffield, UK). We first selected existing PBPK models for our study drugs (morphine, fentanyl, and methadone). We then verified the predictions of the models with PK data from published adult and pediatric PK studies (see S1 File for an in-depth explanation on model selection and verification).

### Model simulations

A total of three different simulations were carried out. The first to see which age groups could be distinguished based on methadone exposure, as exposure is affected by the child's growth and organ maturation. The second to determine the optimal conversion ratio and tapering schedule and the third to examine whether the conversion and tapering should be age-adjusted.

#### Methadone exposure over age.

We simulated a 0.05 mg/kg dose every 12 hours for 10 days in 9 different age groups and compared the area under the curve (AUC) at day 10, when steady state has been reached for every age group (S1 File, section 2.3.2). Age groups with similar exposures were merged. The age group with the lowest exposure and thus lowest maximum plasma concentrations (C_max_), and hence the highest methadone clearance, was used for further simulations to ensure that all age groups would reach the minimum therapeutic methadone concentration.

#### Optimal conversion ratio and tapering protocol.

Different conversion ratios and tapering schedules, which were retrieved from the literature search, were simulated in the age group with the lowest exposure. To visualize the effect of different conversion ratios, the simulations were started with a four-day infusion dose of fentanyl (1 µg/kg/h) or morphine (10 µg/kg/h). After four days, oral methadone administration was initiated using different conversion ratios and after the second dose of methadone, fentanyl and morphine doses were tapered off in three steps. They were halved twice before discontinuation; the first opioid halving coincided with the second methadone dose and discontinuation after the fourth methadone dose [2,10–12]. Thereafter, different methadone tapering protocols were simulated per withdrawal risk category for comparison. To determine the optimal conversion ratio and tapering protocol, we compared the simulated mean C_max_ to the therapeutic target for methadone retrieved from literature.

In addition, for the 1–18-year-old age group, we quantified the proportion of virtual patients achieving the therapeutic target for the tapering protocols. As a clear definition of adequate target attainment is lacking, we evaluated this for the following scenarios: target attainment is achieved if the plasma concentration is at or above the therapeutic target during three different time intervals. We calculated this both cumulatively and consecutively; cumulatively, on the assumption that if a plasma level briefly dips below the therapeutic minimum, this does not immediately lead to withdrawal symptoms, and consecutively by requiring concentrations to remain continuously above the therapeutic minimum, in case this assumption is incorrect. To evaluate if the observed differences in outcomes were statistically different, we conducted two-sided Chi-Squared tests in GraphPad Prism 10.5.0.

#### Effect of age on conversion and tapering.

Finally, we used the optimal conversion ratio and tapering schedule to explore the effect of age on opioid conversion and methadone tapering. Since all three drugs are cleared trough different pathways, e.g., different drug metabolizing enzymes, it is plausible that the ontogeny profiles of these enzymes are responsible for differences in drug exposure between different age groups. Moreover, organ growth and maturation also influence drug kinetics, which may together affect the conversion ratio. Therefore, we ran the same simulations as before, but now in the previously identified age groups, with the optimal conversion ratio and tapering schedules. To determine the effect of age on conversion, we calculated the ratio between the C_max_ values of the opioid and methadone. A less than 2-fold change in this ratio was considered as a minimal age effect.

### Sensitivity analysis

After establishing the optimal tapering protocol, we performed a sensitivity analysis to take into account the robustness of the methadone therapeutic target. In this analysis, we varied the target with steps of 10 ng/mL, and for each age group we assessed the impact on the probability of target attainment (PTA). PTA was evaluated over a 48‑hour interval, starting 48 hours after methadone initiation, which corresponds to the time at which the primary opioid is discontinued and methadone is expected to exert its therapeutic effect, until 48 hours later, at the start of methadone tapering. For every age group, we determined the proportion of children whose methadone concentrations remained at or above the therapeutic target for 100%, 80%, or 50% of this 48-hour interval (cumulatively).

### Establish the dosing recommendation

The modeling results of the conversion ratio and tapering schedules were subsequently discussed with an expert group consisting of four pediatric intensivists (from four Dutch ICUs), four hospital pharmacists (two Dutch centers), two PharmD’s, one general pediatrician, and the managing director of the DPF to establish a dosing recommendation for methadone tapering in critically ill children in the PICU, including an advice for fentanyl and morphine conversion to methadone. Final recommendations were then submitted to the international editorial board of the pediatric formularies for further consensus-based approval.

## Results

### Review of literature

A total of 715 studies were screened for eligibility, of which 28 studies were ultimately found to be relevant for this study (Fig A in S1 File). All 28 studies are summarized in Table D in S1 File.

### Identification and selection of conversion ratios and tapering protocols

#### Conversion ratios.

Seventeen of the twenty-eight included studies report a clear conversion ratio, thirteen for the conversion of fentanyl to methadone and ten for the conversion of morphine to methadone (Table 1) [2–4,10–23]. Furthermore, the Dutch IWS prevention guideline also provides guidance on conversion [9]. The studies show a 26-fold variation in fentanyl and an 8-fold variation in morphine conversion ratios, reflecting different approaches to account for differences in PK and pharmacodynamic (PD) between the drugs. Table 1 shows that opioid potency, elimination half-life, and oral bioavailability were considered differently.

**Table 1:** Conversion ratios by daily dose for fentanyl and morphine to methadone from literature.

Author	Fentanyl IV: PO Methadone		Morphine IV: PO Methadone	
	Ratio	PK/PD adjustment	Ratio	PK/PD adjustment
Ford 2022 [2]	1: 17.3	F = 1: 0.77	4.6: 1	F = 1: 0.77
Wilson 2021 [3]	1: 8.3	Potency = 100: 1 t_½_/F = × 0.5	6: 1	Potency = 2: 1 t_½_/F = × 0.5
Tiacharoen 2020 [13]	1: 26	NA	3.85: 1	NA
Friedman 2020 [14]	1: 5	NA	NA	NA
Robertson 2000 [15] Walters 2019 [4]^1^	1: 16.7	Potency = 100: 1	6: 1	Potency = 1: 1
Solodiuk 2019 [16] Curley 2015 [17]^1^	NA	Not, only dose-based	6: 1	NA
Srinivasan 2017 [11] Abdouni 2016 [18]^1^	1: 2.5	NA	NA	NA
Fife 2016 [19]	NA	NA	7.9: 1	NA
Rotterdam 2016 [9]^2^	1: 3 to 1: 10	NA	1: 1	NA
Jeffries 2012 [20]	NA	NA	1: 1	NA
Bowens 2011 [21]	1: 16.7	Not, only dose-based	NA	NA
Siddappa 2003 [10]	1: 3	Potency = 100: 1 t_½_ = 0.75h: 24h	NA	NA
Meyer 2001 [22]	1: 10^3^	Fentanyl: morphine = 1: 15	1.5: 1	Potency = 0.5: 1
Tobias 1996 [12]	1: 1	No correction needed on potency, t_½_, or F	NA	NA
Tobias 1994 [23]	1: 3	Potency = 100: 1 t_½_ = 0.5h: 24h F = 1: 0.67	NA	NA

^1^These studies have the same source for their conversion ratios.

^2^Most commonly used Dutch guideline for tapering fentanyl and morphine with methadone.

^3^The fentanyl dosage was first converted to morphine (1: 15) and then to methadone. Abbreviations: F: bioavailability, t_½_: elimination half-life, NA: not available

We simulated the conversion ratios for fentanyl according to the two newest publications, i.e., 1:8.3 and 1:17.3, and two conversion ratios that were reported more than once, i.e., 1:3 and 1:10 [2,3,9,10,22,23]. For morphine, we simulated the most frequently reported 6:1 and 1:1 conversion ratios [3,4,9,15–17,20].

#### Tapering protocols.

**Fig 1:**
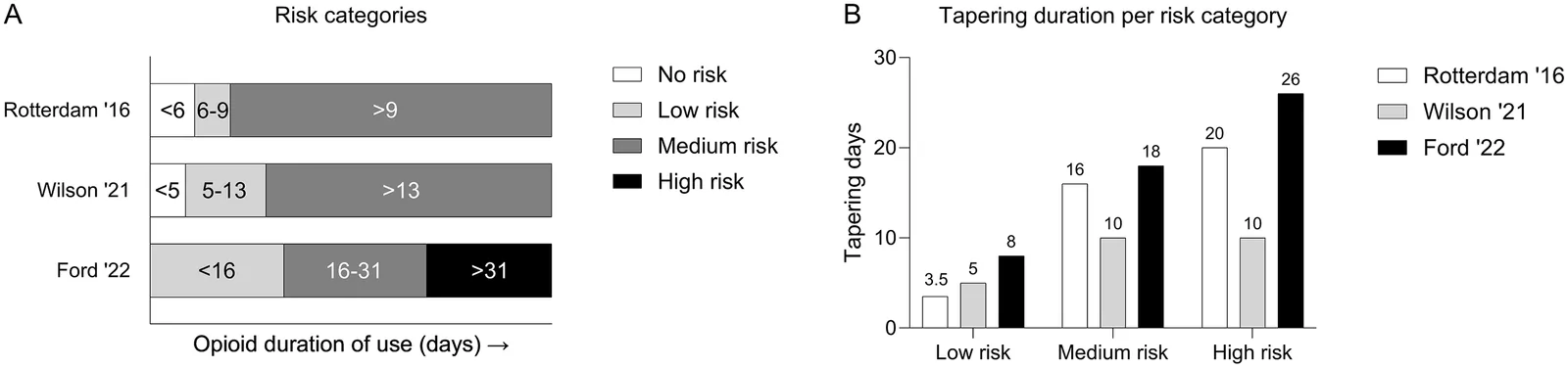
Differences in withdrawal risk categories between methadone tapering protocols. A: the numbers in the bars represent the cut-off values (in days) of withdrawal risk categories per tapering protocol. For the no risk category, no tapering is recommended. B: the duration of each tapering schedule per withdrawal risk category. The exact tapering schedules can be found in Table 2.

Of the 28 included articles, 25 provided information on methadone tapering (Table D in S1 File). Fifteen of these studies lacked details on, for example, the dosing interval, which hampered simulations (Fig A in S1 File). Four studies used a fixed-dose tapering schedule in connection with a fixed conversion, whereby each child received the same starting dose and dosing schedule of methadone regardless of the opioid dose (Fig A in S1 File). Six studies published clearly formulated tapering protocols and distinguished between different withdrawal risk categories [2–4,9,13,15]. However, the protocol by Tiacharoen et al. did not include specifications for the final phase leading up to methadone discontinuation, and the protocol by Wilson et al. is a more comprehensive version of the protocols by Robertson et al. and Walters et al.; consequently, these three protocols were excluded. This resulted in the final selection of three different protocols (i.e., Wilson, Ford, and Rotterdam) for our simulations [2,3,9]. Each protocol consisted of three withdrawal risk categories: risk category 1 (low risk), risk category 2 (medium risk), and risk category 3 high risk.The definition of risk categories (defined by the number of days of opioid use), the total duration of the methadone tapering schedule, and the tapering steps themselves (single dose and dose frequency) varied per protocol. (Fig 1, Table 2).

**Table 2:** Simulated tapering schedules.

Study	7 days opioid use Risk category 1 (Low-risk)	20 days opioid use Risk category 2 (Medium-risk)	>31 days opioid use Risk category 3 (High-risk)	Total tapering duration	Note
Rotterdam [9]	X mg q8h once 0.9*X mg q8h once 0.8*X mg q8h once 0.7*X mg q8h once 0.6*X mg q8h once 0.5*X mg q8h once 0.4*X mg q8h once 0.3*X mg q8h once 0.2*X mg q8h once 0.1*X mg q8h once	X mg q8h 48h 0.9*X mg q8h 48h 0.8*X mg q8h 48h 0.7*X mg q8h 48h 0.6*X mg q8h 48h 0.5*X mg q8h 48h 0.4*X mg q8h 48h† 0.3*X mg q8h 48h 0.2*X mg q8h 48h† 0.1*X mg q8h 48h	X mg q8h 48h 0.9*X mg q8h 48h 0.8*X mg q8h 48h 0.7*X mg q8h 48h 0.6*X mg q8h 48h 0.5*X mg q8h 48h 0.4*X mg q8h 48h† 0.3*X mg q8h 48h 0.2*X mg q8h 48h† 0.1*X mg q8h 48h	After total opioid use of: 7 days: 3.5 days 20 days: 16–20 days >31 days: 20 days † second part of tapering in steps of 10–20% (clinicians’ decision). Medium-risk simulations skipped the †-marked steps.	Max 0.7 mg/kg/day in 2–3 doses 3 risk categories based on duration of opioid use: <6 days (no taper) 6-9 days (low risk) ≥10 days (medium + high risk)
Wilson et al. [3]	X mg q6h 24h 0.8*X mg q6h 24h 0.8*X mg q8h 24h 0.8*X mg q12h 24h 0.8*X mg q24h 24h Stop when ≤0.1 mg/kg/dose If >0.1 mg/kg/dose, taper by 20% of starting dose	X mg q6h 48h 0.8*X mg q6h 48h 0.8*X mg q8h 48h 0.8*X mg q12h 48h 0.8*X mg q24h 48h Stop when ≤0.1 mg/kg/dose If >0.1 mg/kg/dose, taper by 20% of starting dose	X mg q6h 48h 0.8*X mg q6h 48h 0.8*X mg q8h 48h 0.8*X mg q12h 48h 0.8*X mg q24h 48h Stop when ≤0.1 mg/kg/dose If >0.1 mg/kg/dose, taper by 20% of starting dose	After total opioid use of: 7 days: - ≤ 2.5 mg/kg/day: 5 days - > 2.5 mg/kg/day: > 5 days 20 or >31 days: - ≤ 2.5 mg/kg/day: 10 days - > 2.5 mg/kg/day: > 10 days	Max 10 mg/dose 3 risk categories based on duration of opioid use: <5 days (no taper) 5-13 days (low risk) >13 days (medium + high risk)
Ford et al. [2]	X mg q6h 48h X mg q8h 48h X mg q12h 48h X mg q24h 48h	X mg q6h 48h 0.85*X mg q6h 48h 0.85*X mg q8h 48h 0.7*X mg q8h 48h 0.6*X mg q8h 48h 0.45*X mg q8h 48h 0.45*X mg q12h 48h 0.3*X mg q12h 48h 0.3*X mg q24h 48h	X mg q6h 48h 0.9*X mg q6h 48h 0.8*X mg q6h 48h 0.7*X mg q6h 48h 0.7*X mg q8h 48h 0.6*X mg q8h 48h 0.5*X mg q8h 48h 0.4*X mg q8h 48h 0.4*X mg q12h 48h 0.3*X mg q12h 48h 0.2*X mg q12h 48h 0.1*X mg q12h 48h 0.1*X mg q24h 48h	After total opioid use of: 7 days: 8 days 20 days: 18 days >31 days: 26 days	3 risk categories based on duration of opioid use: ≤15 days (low risk) 16-31 days (medium risk) >31 days (high risk)

† second part of tapering in steps of 10–20% (up to the clinicians’ decision). When chosen for steps of 20% (fast tapering), the steps with * can be skipped. Abbreviations: h: hours, qXh: every X hours

### Identification of therapeutic targets

For fentanyl and morphine, reported analgesic plasma concentrations in children range between 1–3 ng/mL and 4–65 ng/mL, respectively [24,25]. Both ranges are based on data from children that received a fentanyl or morphine infusion after cardiac surgery. As we primarily simulated the starting dose of these opioids, we assessed whether simulated plasma concentrations reached the minimal therapeutic thresholds (1 ng/mL for fentanyl, 4 ng/mL for morphine). In addition, we also evaluated whether plasma concentrations above the upper limit of the therapeutic range were reached to assess the risk of toxicity.

For methadone, a minimal effective plasma concentration of 60 ng/mL to prevent IWS is described for neonates [26]. At this concentration, neonates of mothers who used methadone during pregnancy did not suffer from withdrawal symptoms. A similar postoperative methadone concentration (58 ng/mL) is found for adults as the minimal analgesic concentration [27]. As we could not identify additional data on therapeutic concentrations for children aged 1 month to 18 years, we assumed that a methadone plasma concentration of at least 60 ng/mL is needed to avoid withdrawal when converting opioids in these children. No strict toxic threshold has been reported for methadone, as habituation complicates this, leading to tolerance of increasingly higher concentrations.

While the concentration-effect relationship may be subject to age-related variation, data are too scarce to take into account, and hence we used for all age groups the same therapeutic target concentration [28].

### Selection and verification of the PBPK models

Verification of the PBPK models of R-methadone, S-methadone, fentanyl, and morphine proved successful, as all models accurately described PK (for more in-depth information on the verification, see the S1 File).

### Model simulations

#### Methadone exposure over age.

The 10-day simulation of 0.1 mg/kg/day shows that the exposure in children aged 1–18 years is approximately similar, hence, we merged them into one age group (S1 File 2.3.2). The 1–18-years-olds also show the lowest AUC values, so this age group was used for simulations of different conversion ratios and tapering protocols.

### Optimal conversion ratio and tapering protocol

#### Conversion ratios.

**Fig 2:**
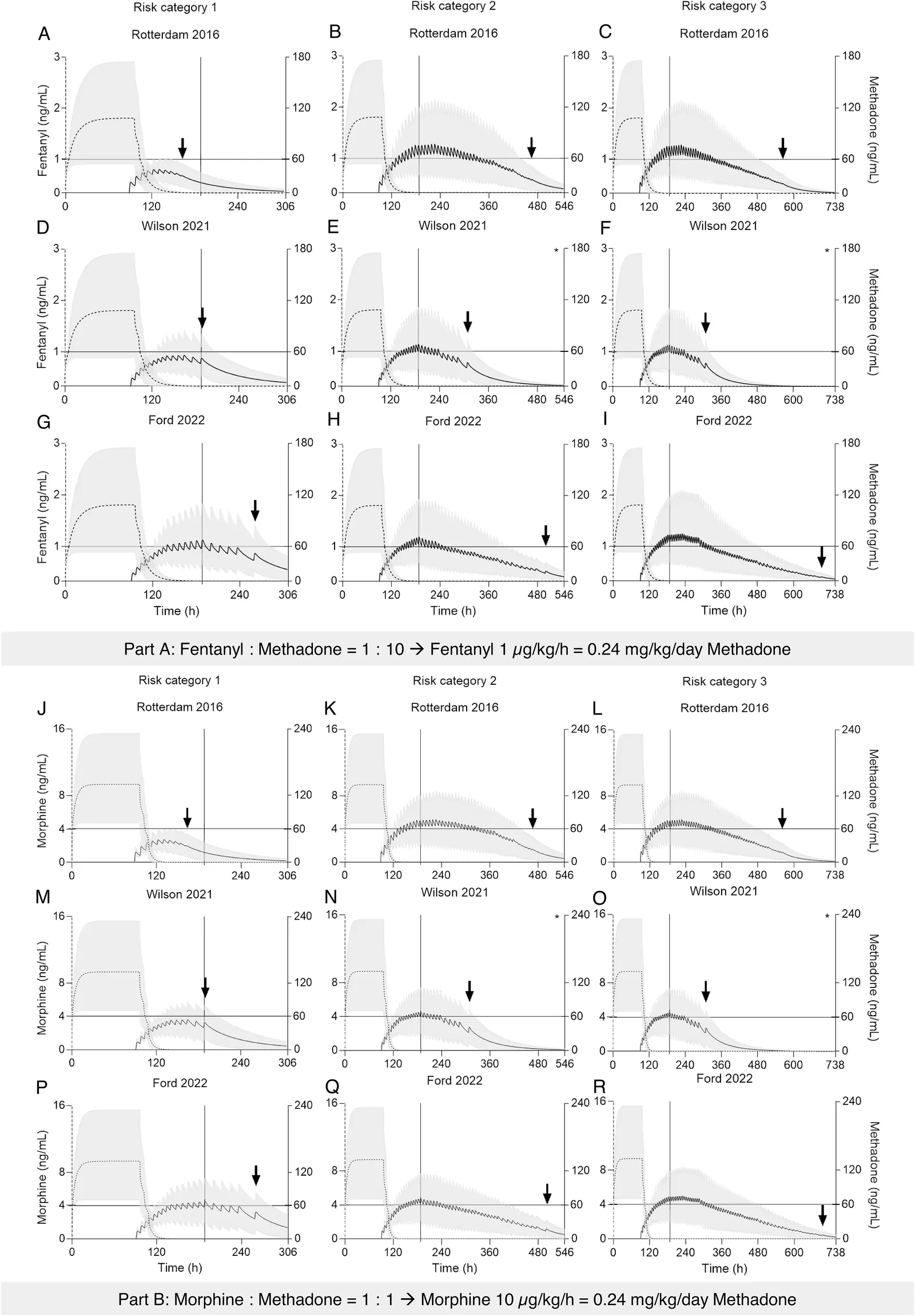
Simulations of a 1:10 conversion of fentanyl to methadone and a 1:1 conversion of morphine to methadone, tapered according to three tapering schedules. A 1 µg/kg/h infusion of fentanyl (part A, graph A-I) and a 10 µg/kg/h infusion of morphine (part B, graph J-R) are both converted to 0.24 mg/kg/day of methadone. Each row represents a tapering protocol, each column a withdrawal risk category. The left (dotted) y-axis represents the fentanyl (part A) or morphine (part B) concentration, the right y-axis the methadone concentration. The dotted line is the mean predicted plasma concentration of fentanyl (A) or morphine (B), the bold black line is the mean predicted plasma concentration of methadone. The gray areas are the 5^th^ to 95^th^ percentile of these predictions. The black horizontal line represents the minimal analgesic concentration of fentanyl (1 ng/mL, part A) of morphine (4 ng/mL, part B) and the minimal methadone concentration needed to avoid withdrawal symptoms (60 ng/mL). To compare the timing of C_max_, a black vertical line at t = 88 hours after the initiation of methadone administration was drawn, with the black arrows indicating the last administered dose. The methadone tapering schedules are simulated in 1-18-year-olds. *Similar tapering schedule.

The effect of the different conversion ratios and tapering protocols on drug concentration in 1–18-year-olds is illustrated in Fig 2 and D-G in S1 File. With regard to the conversion ratio, Fig 2 shows that a 1:10 conversion of fentanyl and a 1:1 conversion of morphine both lead to equal methadone starting doses, resulting in predicted mean C_max_ values close to the 60 ng/mL threshold for most tapering schedules. In contrast, a 1:8.3 and a 1:3 conversion of fentanyl led to mean methadone concentrations below the 60 ng/mL threshold for all tapering schedules, while the 1:17.3 conversion resulted in a mean methadone concentration far above this threshold. For morphine, a 6:1 conversion resulted in subtherapeutic methadone concentrations.

#### Tapering protocols.

**Fig 3:**
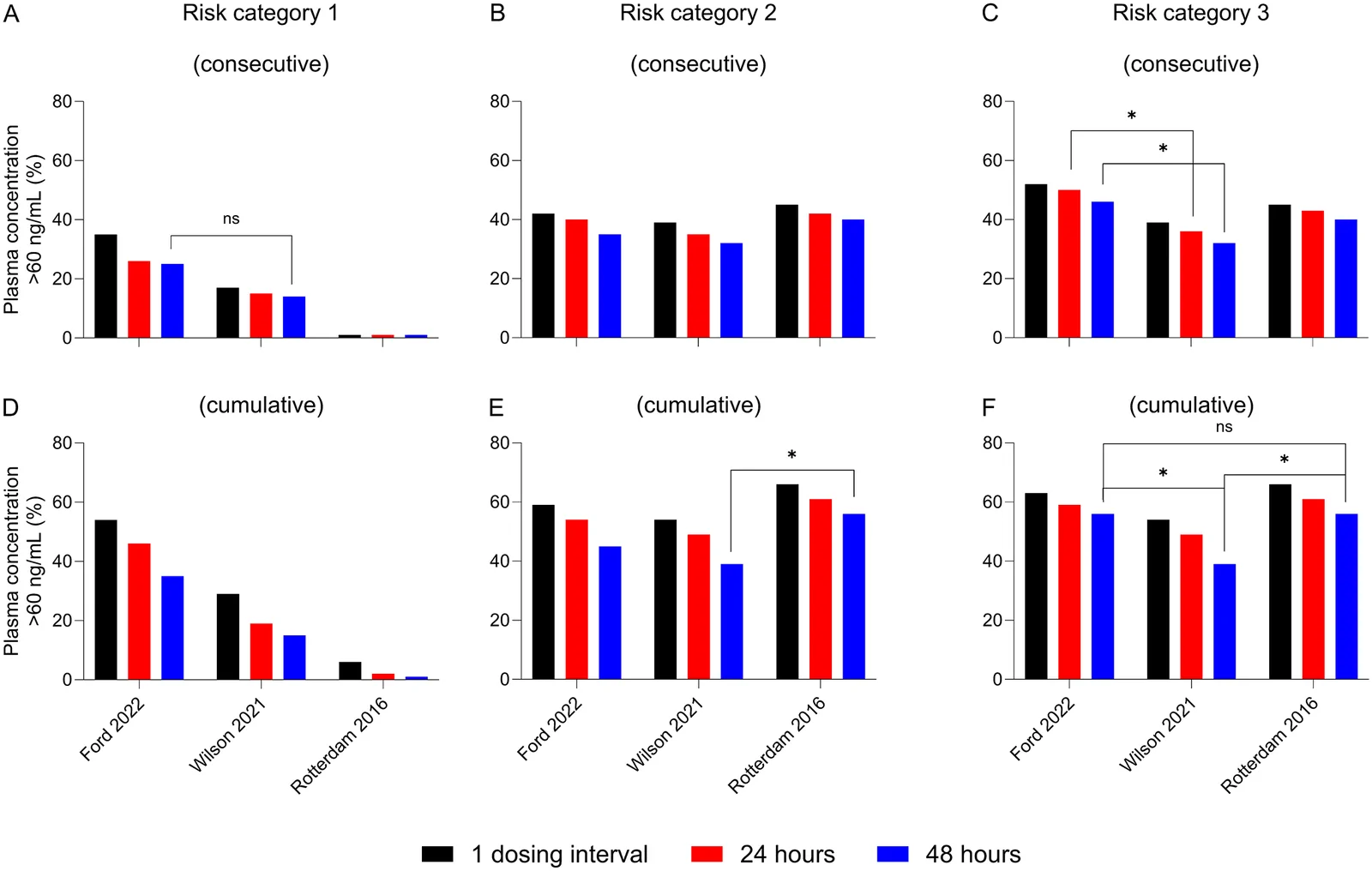
Proportion of patients reaching therapeutic target. For every risk category and every tapering protocol, the proportion of virtual patients reaching the therapeutic target of 60 ng/mL is calculated for three time intervals, consecutively and cumulatively. Panels 3A and 3D demonstrate statistically significant different percentages of patients reaching therapy target, with the exception of the 48-hour consecutive interval of Ford and Wilson. Panels 3B and 3E show comparable target attainment across protocols, aside from the 48-hour cumulative interval where Wilson and Rotterdam diverge. Panels 3C and 3F also show almost equivalent target attainment, with the exception of the 24- and 48-hour consecutive intervals of Ford and Wilson and the 48-hour cumulative interval in which Wilson differs significantly from both Ford and Rotterdam.

Simulations of the different tapering protocols after conversion show that therapeutic methadone concentrations (with mean C_max_ values of 60.3 to 68.5 ng/mL) were achieved for seven out of nine tapering schedules (Fig 2). The time of C_max_ attainment was also quite similar (ranging from 90.4 to 98.5 hours). To further quantify target attainment, we calculated the proportion of virtual patients reaching methadone plasma concentrations ≥60 ng/mL for three different time intervals: 1) one dosing interval (6 hours for Ford and Wilson, 8 hours for Rotterdam), 2) 24 hours, and 3) 48 hours, both cumulatively and consecutively for each time interval. Fig 3 clearly shows that for risk category 1, the percentage of patients reaching a plasma concentration of at least 60 ng/mL is higher when using the Ford protocol compared to the other protocols. For risk category 2, no significant differences are apparent between the protocols, except for the 48-hour cumulative interval of Wilson and Rotterdam. The same largely applies to risk category 3, with the exception of the Wilson protocol, showing a significantly lower proportion of patients reaching the 60 ng/mL target for at least 24 hours (consecutively) and 48 hours (both consecutively and cumulatively). Overall, the Ford protocol demonstrated the highest and most optimal rates of target attainment.

### Effect of age on conversion and tapering

**Fig 4:**
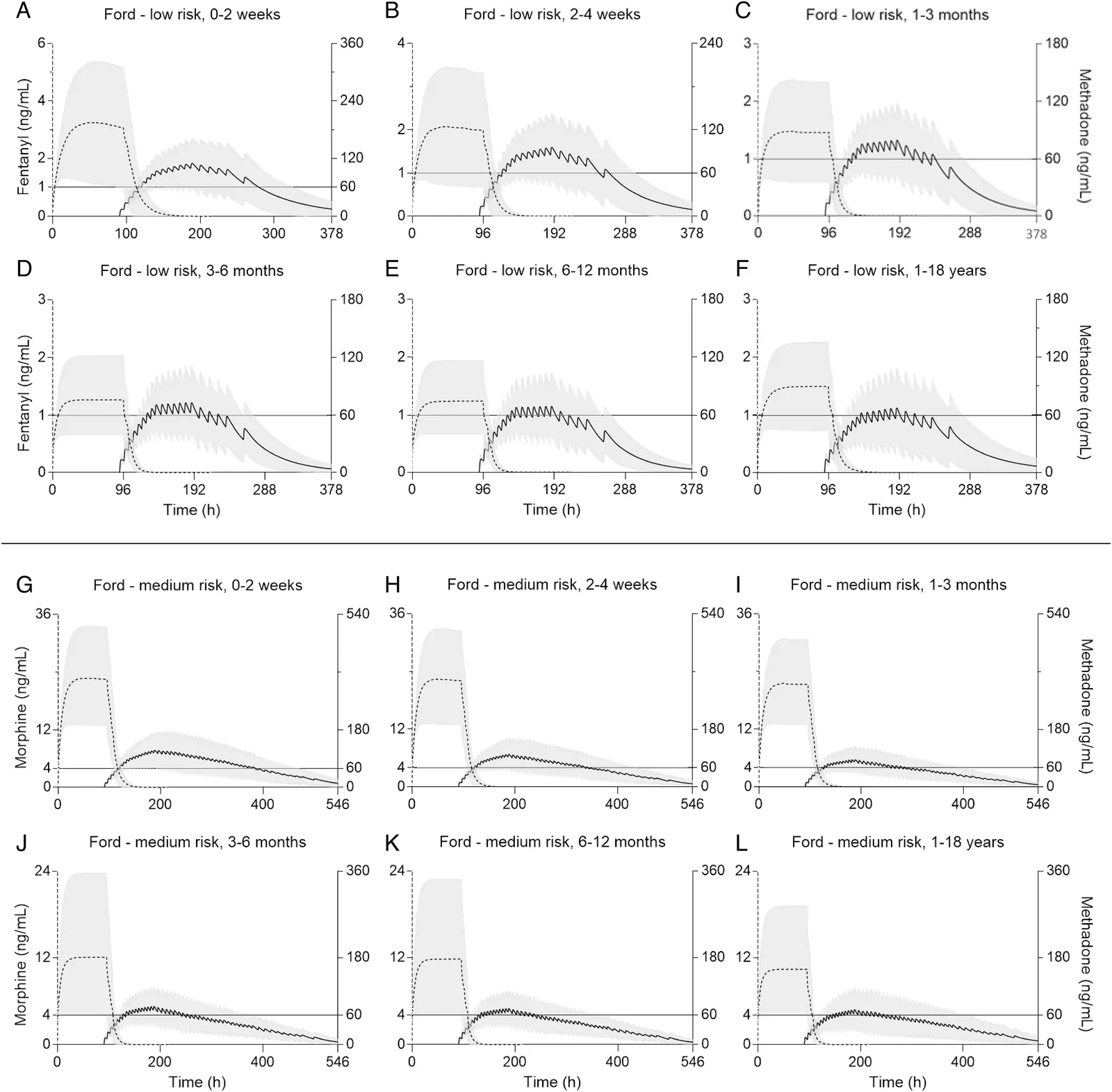
The effect of age on fentanyl and morphine conversion and methadone tapering. For each age group, fentanyl 1 µg/kg/h and morphine 10 µg/kg/h infusions are 1: 10 and 1: 1 converted to methadone, respectively. The left (dotted) y-axis represents the fentanyl (A-G) or morphine (H-N) concentration, the right y-axis the methadone concentration. The dotted line is the mean predicted plasma concentration of fentanyl or morphine, the black line is the mean predicted plasma concentration of methadone. The gray areas are the 5^th^ to 95^th^ percentile of these predictions. The black horizontal line represents the minimal analgesic concentration of fentanyl (1 ng/mL) or morphine (4 ng/mL) and the minimal methadone concentration needed to avoid withdrawal symptoms (60 ng/mL). For fentanyl and morphine, the low- and medium-risk withdrawal categories from the Ford protocol were simulated, respectively.

Fig 4 and Fig H in S1 File show that age mainly affects neonatal drug exposure. For fentanyl and morphine, the mean simulated plasma concentrations remained safely within the therapeutic window. Few neonates showed higher opioid concentrations, which might indicate oversedation, necessitating dose reductions. C_max-opioid_ to C_max-methadone_ ratios were calculated per age group (Table 3) to evaluate whether age affects opioid conversion. For both opioids, these ratios did not change more than 2-fold, indicating a minimal effect of age on conversion. To evaluate the age effect on methadone tapering, we compared methadone plasma concentration-time curves between the different age groups. Neonatal methadone C_max_ was higher than the C_max_ values in older children. Although no upper limit has been specified for this C_max_, the mean C_max_ in neonates remained within twofold that in older children, with mean C_max_ values of 108 and 92 ng/mL at ages 0–2 and 2–4 weeks, respectively.

**Table 3:** C_max_ ratios of opioid to methadone conversions.

Age	Fentanyl-methadone ratio	Morphine-methadone ratio
0-2 weeks	0.025	0.20
2-4 weeks	0.018	0.23
1-3 months	0.016	0.26
3-6 months	0.019	0.12
6-12 months	0.018	0.12
1-18 years	0.022	0.12

### Sensitivity analysis

**Fig 5:**
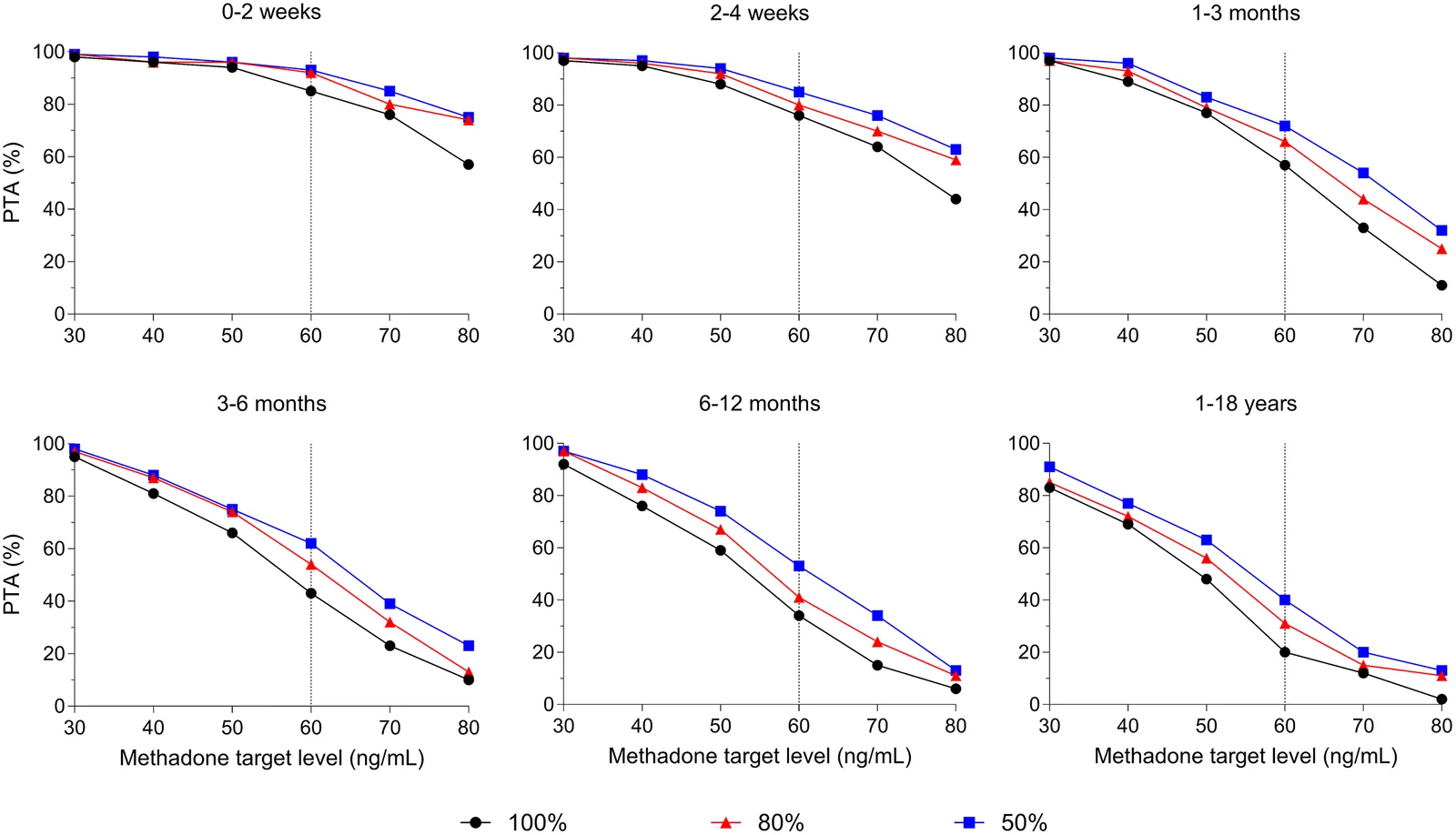
Sensitivity analysis of the therapeutic target of methadone. For every age group, a sensitivity analysis is carried out to evaluate the effect of a potentially incorrectly chosen therapeutic target for methadone. The X-axis shows the six different therapeutic targets studied, and the y-axis shows the PTA. Target attainment is assessed between 48 and 96 hours after the initiation of methadone and calculated for the full-time interval (100%, from 48 to 96 hours consecutively, black circles), 80% of the time interval (cumulative, red triangles), and 50% of the time interval (cumulative, blue squares). The dotted grey vertical line at 60 ng/mL is the therapeutic target used in this manuscript.

To explore the effect of a change in therapeutic target, we assessed the PTA at three different levels: 100%, 80%, and 50% of time (cumulative) above a varying therapeutic target (30–80 ng/mL). The results of the analysis for each age group are shown in Fig 5. PTA decreases with increasing age, which is also shown in Table 4 for the 60 ng/mL therapeutic target.

**Table 4:** Probability of target attainment at 60 ng/mL.

%T ≥ 60 ng/mL	0-2 weeks	2-4 weeks	1-3 months	3-6 months	6-12 months	1-18 years
100	85%	76%	57%	43%	34%	20%
80	92%	80%	66%	54%	41%	31%
50	93%	85%	72%	62%	53%	40%

Abbreviation: %T > 60 ng/mL; percentage of time that the plasma concentration is ≥ 60 ng/mL

### Establish the dosing recommendation

In an online meeting, the above-mentioned results were discussed with the working group, to complement our modeling results with clinical expert opinion. This resulted in the following recommendations: 1) fentanyl should be converted 1:10 to methadone, 2) morphine should be converted 1:1 to methadone, 3) a modified tapering schedule of the Ford protocol is adopted: the medium risk tapering schedule for the medium and high risk categories and no tapering for the low risk category (Table 5), for two reasons. First, the high-risk withdrawal tapering schedule is not adopted because the proposed duration (26 days) was considered too long and, according to clinical expertise, the same results can be achieved with the medium-risk tapering schedule. Second, the panel believed that, based on clinical experience, children who have used an opioid for less than five days do not need to be converted to methadone (addition of a no-risk category). The modified recommendations were also accepted by the international editorial board and are available on the Pediatric Formulary websites (www.kinderformularium.nl, www.kinderformularium.de, www.kindermedika.at, and www.koble.info [29]).

**Table 5:** Final methadone tapering schedule.

No-risk category <5 days opioid use	Low-risk category 5-15 days opioid use	Medium-risk category >15 days opioid use
No conversion to methadone	X mg q6h 48h X mg q8h 48h X mg q12h 48h X mg q24h 48h	X mg q6h 48h 0.85*X mg q6h 48h 0.85*X mg q8h 48h 0.7*X mg q8h 48h 0.6*X mg q8h 48h 0.45*X mg q8h 48h 0.45*X mg q12h 48h 0.3*X mg q12h 48h 0.3*X mg q24h 48h

Abbreviations: h: hours, qXh: every X hours

## Discussion

This study combined PBPK modeling and simulation with consensus-based decision-making to tackle a complex clinical question on how to optimally taper off opioids using methadone to prevent withdrawal symptoms in PICU patients. Reviewing the literature revealed a huge variety of conversion ratios and tapering protocols, but in the absence of comparative studies, it was impossible to determine the most optimal ones. Our simulations indicated that a 1:10 conversion ratio for fentanyl and a 1:1 conversion ratio for morphine achieved adequate mean methadone concentrations for most of the withdrawal risk categories. Further simulations showed that the tapering protocol by Ford optimally attains the minimum therapeutic concentration across all withdrawal risk categories. These insights were translated into a minimally adjusted Ford protocol, which is directly implemented in the DPF, first in consultation with Dutch clinicians and then with the international editorial board of the Pediatric Formularies.

The wide range of conversion ratios in literature can be explained by the different approaches used to determine them and the influence of previous opioid exposure and degree of opioid tolerance [1,30]. Interestingly, reported rates of withdrawal symptoms and oversedation were quite similar for the different conversion ratios. For example, for both the highest and lowest fentanyl conversion ratios (i.e., 1:17.3 and 1:1), withdrawal symptoms were reported in 17% of children [2,12]. Although the highest conversion ratio used by Ford resulted in higher doses of methadone, oversedation is not mentioned, while Tobias et al. reported 11% oversedation with a 1:1 conversion. For morphine, for both extreme conversion ratios of 7.9:1 and 1:1, a similar 42% of withdrawal symptoms was reported [14,17]. These divergent results can largely be explained by differences in study design, such as initial methadone dose, withdrawal risk based on duration of opioid use, dosing intervals, tapering duration, whether or not withdrawal symptom scoring tools are used, and patient characteristics and setting [1]. This complicates the comparison of the results, making it impossible to determine the optimal conversion based on literature only. However, with this modeling and simulation study, we support this decision-making, by simulating target therapeutic concentrations.

The choice of tapering protocol based on literature is difficult, as studies only compare the use of a protocol versus non-use. The use of protocols, however, appears to reduce withdrawal symptoms and shorten tapering duration [1–4,15,20]. Our study visualized the drug concentration differences between tapering protocols, showing that the tapering schedules for the low-risk withdrawal category of Wilson and the Rotterdam protocol led to lower methadone levels than all other withdrawal risk categories. This difference is directly attributable to the respective dosing regimens, as the initial methadone dose is only maintained for 8 and 24 hours according to the Rotterdam and Wilson protocols, respectively, while Ford recommends to maintain the initial dose for 48 hours (Table 2). However, withdrawal rates do not fully align with this: Ford reported 17% compared to 1% by Wilson [2,3]. This difference may be due to different criteria for continuation of tapering. Both used the Withdrawal Assessment Tool-1 (WAT-1), but Wilson continued tapering at WAT-1 scores <3, while Ford used a score of <4. This means that children treated according to the Wilson protocol show less withdrawal than those treated according to the Ford protocol, which also explains the almost equal percentage of rescue doses administered (i.e., 3.9% and 2.8%, respectively). Oversedation, however, was not mentioned by Ford, while Wilson reported oversedation in only 1% of all patients.

Side effects of methadone, such as oversedation, in children in the PICU are rarely reported, and data on toxicity are even more limited. The few reports available reporting toxic, but not fatal, methadone plasma concentrations indicate a range of 30–360 ng/ml, of which it is unclear at what time after ingestion these levels were measured [31,32]. Only Sadhasivam et al. and Watt et al. used safety thresholds; Sadhasivam et al. used an unsubstantiated safety limit of 100 ng/mL perioperatively, which they associated with the occurrence of respiratory depression and Watt et al. based their surrogate safety target of 300 ng/mL on studies by Inturrisi et al. who examined the use of methadone in adults with chronic cancer-related pain [33–36]. However, it is difficult to extrapolate either of these situations to the PICU setting, as children using methadone perioperatively are likely to be opioid-naive, and the pathophysiology of chronic cancer-related pain differs significantly from that of acute pain as seen in the PICU [37,38]. Nevertheless, our simulations have shown that only the mean neonatal methadone concentrations approached the 100 ng/mL limit, while maximum concentrations never exceeded 180 ng/mL. In addition, it should be noted that the simulated fentanyl and morphine concentrations are also higher compared to older children, suggesting that neonates might require a lower dose than older children. Reducing fentanyl and morphine dosages based on enzyme ontogeny to achieve exposures comparable to those in older children would consequently result in lower methadone exposure after conversion. Interestingly, such a dose reduction is already proposed for morphine [28].The most crucial phase of opioid tapering is the time between the initiation of methadone and the discontinuation of the other opioid. A balance must be struck between withdrawal and oversedation while tapering off the IV opioid and achieving effective methadone plasma concentrations. The long half-life of methadone (about 24 hours) complicates this further, as it takes three to five days to reach therapeutic concentrations. Therefore, it is important not to discontinue the fentanyl or morphine, which both have shorter half-lives, too early, to prevent withdrawal symptoms when their concentration may be already subtherapeutic while methadone is not clinically effective yet. In most studies, opioid reduction is therefore only started after the second dose of methadone [2,10–12]. Our simulations showed that this results in the plasma concentration of the opioid falling below the therapeutic threshold, while methadone has not yet reached a therapeutic concentration. Nevertheless, the synergistic nature of the combination of these drugs will likely prevent withdrawal symptoms, but this needs further study [39]. Ward et al. simulated several loading doses that would allow methadone to reach steady state more quickly, but to our knowledge, these schedules have not yet been applied in clinical practice [40]. In addition, these relatively high loading doses require caution as they could increase the occurrence of serious side effects, such as respiratory depression, QTc prolongation and arrhythmia, but also less severe but common side effects such as nausea and vomiting [41]. For safety reasons, we did not include such a dosing schedule in our proposal.

One of the strengths of this study is its unique perspective by combining literature review, PBPK modeling and clinical experts to support clinical decision on the opioid tapering process. Visualization of the conversion and tapering provided insights that could not be obtained from literature. Moreover, this PBPK approach is an ethically responsible, time-saving, and less expensive solution than, for example, a clinical pharmacokinetic trial. In addition, our study results were directly discussed with a Dutch clinical expert group and implemented in Dutch, German, Austrian, and Norwegian guidelines, making the model-informed recommendations fit for use in clinical practice. The generalizability beyond the Dutch and European setting may be affected by population-specific variations in methadone pharmacokinetics. Variability in pharmacogenetic factors, including CYP2B6 polymorphisms, is associated with differences in methadone metabolism, exposure, and dose requirements among different ethnicities [42–46]. Consequently, the suggested conversion and tapering protocol should be understood in relation to local patient characteristics and clinical practice.

However, our study does have some limitations. First, the inability to account for opioid tolerance. When children need a continuous opioid infusion for longer periods of time, the likelihood of developing tolerance is high [30]. In case of tolerance, a dose increase can generally overcome this [30], with an increased therapeutic opioid plasma level as a result. As our opioid to methadone conversion ratios are dose-dependent, this inherently results in an increased methadone dose, implying that the methadone therapeutic window shifts up. Although the exact mechanism of opioid tolerance is unknown, current evidence suggests that tolerance arises from a combination of neuronal adaptations and opioid receptor changes (i.e., desensitization, internalization, and downregulation), indicating that opioid tolerance occurs across the entire opioid class [47]. However, the phenomenon of incomplete cross-tolerance suggests that tolerance patterns differ between various opioids, resulting in the requirement of lower than equianalgesic dosages of the converted opioid [30]. In addition, methadone is able to partially reverse opioid tolerance through N-methyl-D-aspartate (NMDA) receptor antagonism and the higher the opioid tolerance, the greater the effect of this antagonism [30,47]. Both mechanisms may allow effective treatment with doses lower than those predicted by equianalgesic conversion ratios [48]. However, these effects have mainly been described in patients undergoing opioid rotation because of tolerance and hyperalgesia, rather than during opioid tapering. Tolerance is therefore expected to be less pronounced in our clinical setting, which may reduce its impact on conversion. Furthermore, the opioid conversion ratios evaluated in the present study were derived from clinical trials, and we assumed that some impact of tolerance may already be captured into the conversion ratios, since they were applied in clinical practice. And while the above may argue for a more conservative conversion in opioid tolerance, the high rate of undertreatment actually argues for a less conservative conversion, underscoring the importance of continuing to monitor for withdrawal symptoms and excessive sedation during the conversion [1,30].

Second, the therapeutic window of all opioids is extrapolated from clinical settings that do not all reflect the long-term pediatric ICU patient, leaving uncertainty as to whether the minimum therapeutic concentration is applicable to our target population. It is known that the pharmacodynamics of opioids in children differ from those in adults, which is why, for methadone, we performed a sensitivity analysis on the therapeutic target. This way, we showed how the PTA is affected by the choice of therapeutic target, including the purely adult-based efficacy threshold of 30 ng/mL [49,50]. Additionally, the observed decrease in PTA with increasing age should be interpreted in light of this uncertainty considering the therapeutic target. A lower PTA in older children suggests that fewer patients attained the predefined target concentration of 60 ng/mL, but does not necessarily indicate undertreatment. As the generalizability of the selected target concentration in our target population remains uncertain, and the required duration above the minimum target attainment to prevent withdrawal symptoms is unknown, the clinical significance of the age-related decline in PTA is unclear.

## Conclusion

This study presents a truly unique and pragmatic approach to decision-making in cases where existing literature does not provide sufficient guidance. By using PBPK modeling, we offer model-informed dosing recommendations as a solution to a pressing clinical need—particularly valuable when traditional evidence is lacking. This strategy, while not commonplace, is not without precedent. Similar model-based methodologies have been successfully implemented in areas facing similar gaps in evidence: for instance, in optimizing gentamicin dosing in neonates and infants [8], determining age-specific dexamethasone doses to prevent post-extubation stridor in children [51], and supporting drug use in pregnancy where clinical data are limited [52]. At present, our findings represent the best available support for opioid tapering as a complement to existing guidelines. Nevertheless, prospective studies remain essential when introducing new tapering protocols—enabling comparison with existing strategies and helping refine best practices. Additionally, more robust reporting of adverse events is needed to better understand potential toxicity and enhance patient safety. To truly assess the effectiveness and safety of the implemented conversion and tapering strategy of this study, a real-word data comparison pre- and post-implementation or a randomized controlled trial are essential. Together, these efforts will help solidify the scientific foundation for model-informed, evidence-based opioid tapering in clinical practice.

## Supporting information

S1 File Supporting information file. (PDF)
