# Supplementary material for: Supporting clinical guidelines for opioid conversion to methadone and tapering to prevent withdrawal in critically ill children using physiology based pharmacokinetic modeling and simulation
Source: PLoS One. 2026 Sep 8;21(9):e0356917. doi: 10.1371/journal.pone.0356917 (PMC13552780; doi:10.1371/journal.pone.0356917)
Supplement: Supplementary file 1 [file pone.0356917.s001.pdf]

Supporting information file

Supporting clinical guidelines for opioid conversion to methadone and tapering to prevent withdrawal in critically ill children using physiology based pharmacokinetic modeling and simulation

Authors: Marika A. de Hoop-Sommen*, Sahar Alozai*, Violette M.G.J. Gijsen, Tjitske M. van der Zanden, Henry T. Akinbi, Tomoyuki Mizuno, Alexander A. Vinks, Rick Greupink, Saskia N. de Wildt

*Both authors contributed equally

Content

1. Literature review 2
1.1 Methods 2
1.2 Literature search queries 2
1.3 Results literature review 5
2. PBPK modeling 56
2.1 Methods 56
2.2 Literature search queries 57
2.3 Results PBPK modeling 58
2.4 Pharmacokinetic studies 66
2.5 References 71
Appendix 1. Model verification; methadone in adults 75
Appendix 2. Model verification; methadone in pediatrics 80
Appendix 3. Model verification; methadone in neonates 82
Appendix 4. Model verification; fentanyl 83
Appendix 5. Model verification; morphine 85

1. Literature review

1.1 Methods

Studies were selected based on criteria to identify publications regarding opioid tapering and methadone administration in critically ill patients. Eligible studies were pediatrics between 1 month and 18 years who used morphine or fentanyl and undergo opioid tapering procedure. The review was not restricted by the date of publication. Included publications were limited to English-language. Studies focusing on the use of methadone in opioid withdrawal, addressing the conversion from morphine or fentanyl to methadone and the associated initial dosing regimen during the weaning process were included. In addition, research that described several approaches for tapering opioids after conversion to methadone was included. All study designs were considered in the analysis, excepting reviews and editorials. Exclusion criteria included studies targeting on neonates with neonatal abstinence syndrome. Studies that investigated methadone for the treatment of acute or chronic pain, rather than for managing of opioid withdrawal, were excluded for review.

1.2 Literature search queries

Multiple terms were combined and used across various fields, including Title/Abstract, MeSH, and All Fields. Several terminology, including "infant," "toddler," "minor," "boy," "girl," "kid," "child," "adolescent," "juvenile," and "pediatric," were used to ensure comprehensive coverage of the pediatric population. Key search terms used were ''methadone/administration and dosage'', ''methadone/therapeutic use'', ''morphine'', ''fentanyl'', ''substance withdrawal syndrome'', ''opioid withdrawal'', ''weaning protocol'', 'wean'', ''taper''. Search queries are shown in Tables A-C and a flow diagram of included studies in Figure A. Summaries of included studies are reported in Table D.

Table A. Search strategy PubMed

PubMed Search #	Search Term (blue: selected individually; black: standardized	Results
#1	(Infan* OR toddler* OR minors OR minors* OR boy OR boys OR boyfriend OR boyhood OR girl* OR kid OR kids OR child OR child* OR children* OR schoolchild* OR schoolchild OR school child[tiab] OR school child*[tiab] OR adolescen* OR juvenil* OR youth* OR teen* OR under*age* OR pubescen* OR pediatrics[mh] OR pediatric* OR paediatric* OR peadiatric* OR school [tiab] OR school*[tiab])	7071.225
#2	"methadone/administration and dosage"[MeSH] OR "methadone/therapeutic use"[MeSH] OR "methadone" [MeSH] AND Therapeutic uses [MeSH] OR (methadone [tiab] AND (administration and dosage* [tiab] OR therapeutic use* [tiab]))	7.378
#3	(#1+#2)	1.668
#4	#3 AND opioid withdrawal [All Fields]	316
#5	#4 NOT neonatal abstinence syndrome [All Fields]	179
#6	#5 NOT pregnant [All Fields]	158
#7	#6 AND weaning protocol [All Fields]	8
#8	#1 AND methadone [All Fields] AND opioid withdrawal [All Fields] AND weaning protocol [All Fields]	20
#9	#3 AND morphine [MeSH] OR fentanyl [MeSH] AND substance withdrawal syndrome [MeSH]	213
#10	#9 AND wean [All Fields] OR taper [All Fields]	24

Search 6: 158 results → screened based on abstract and titles → 35 selected and 123 excluded → 27 included and 8 excluded
Search 7: 8 results → screened based on abstract and titles → all 8 fall within search 6
Search 8: 20 results → screened based on abstract and titles → 11 fall within search 6 → 1 newly included and 8 excluded
Search 9: 213 results → screened on the basis of abstract and titles → 17 fall within search 6 → 1 newly included and 195 excluded
Search 10: 24 results → screened based on abstract and titles → 5 fall within search 6 and 9 (19 previously excluded)

Table B. Search strategy Embase

Embase Search #	Search Term (blue: selected individually; black: standardized	Results
#1	((Infan* OR toddler* OR minors OR minors* OR boy OR boys OR boyfriend OR boyhood OR girl* OR kid OR kids OR child OR child* OR children* OR schoolchild* OR schoolchild OR school) AND child.ti,ab. OR school) AND child*.ti,ab. OR adolescen* OR juvenil* OR youth* OR teen* OR under*age* OR pubescen* OR pediatric* OR paediatric* OR peadiatric* OR school.ti,ab. OR school*.ti,ab. OR 'pediatrics'/exp	4.691.196
#2	'methadone'/exp AND (administration:ti,ab OR dosage*:ti,ab OR 'therapeutic use':ti,ab) OR ('methadone'/exp AND 'therapeutic use'/exp) OR (methadone:ti,ab AND ('administration and dosage*':ti,ab OR 'therapeutic use*':ti,ab))	5.268
#3	(#1+#2)	478
#4	#3 AND opioid AND withdrawal [All Fields]	81
#5	#4 NOT neonatal abstinence syndrome [All Fields]	68
#6	#5 NOT pregnant [All Fields]	64
#7	#6 AND weaning protocol [All Fields]	5
#8	#1 AND methadone [All Fields] AND opioid withdrawal [All Fields] AND weaning protocol [All Fields]	27
#9	#3 AND ('morphine'/exp OR 'fentanyl'/exp) AND 'substance withdrawal syndrome'/exp	51
#10	#9 AND wean [All Fields] OR taper [All Fields]	4

Search 6: 64 results → screened based on abstract and titles → 7 already included from PubMed searches* → 2 selected and 55 excluded → 2 newly included and 55 excluded

Search 7: 5 results → screened based on abstract and titles → 2 already included from PubMed searches** → 0 selected and 3 excluded

Search 8: 27 results → screened based on abstract and title → 4 already included from PubMed searches*** → 1 selected and 22 excluded → 1 newly included and 22 excluded

Search 9: 51 results → screened on the basis of abstract and titles → 4 already included from PubMed searches**** → 1 already included in search 6 → 0 selected and 46 excluded

Search 10: 4 results → screened based on abstract and title → 3 already included from PubMed searches***** → 0 selected and 1 excluded

* Fife 2016, Jeffries 2012, Lugo 2001, Siddappa 2003, Wilson 2021

** Jeffries 2012, Wilson 2021

*** Ford 2022, Jeffries 2012, Steineck 2014, Wilson 2021

**** Jeffries 2012, Siddappa 2003, Fife 2016, Lugo 2001

***** Lugo 2001

Table C. Search strategy CINHAL

CINHAL Search #	Search Term (blue: selected individually; black: standardized	Results
#1	(MH "Infant+" OR MH "Child+" OR MH "Adolescent+" OR TX (infant* OR toddler* OR minor* OR child* OR schoolchild* OR adolescent* OR pediatric* OR paediatric*))	1.937.454
#2	"methadone/administration and dosage"[MeSH] OR "methadone/therapeutic use"[MeSH] OR "methadone"[MeSH] OR ((methadone[tiab] AND (administration and dosage*[tiab])) OR therapeutic use*[tiab])	5.808
#3	(#1+#2)	1.130
#4	#3 AND (MH "Substance Withdrawal Syndrome" OR TX (opioid* AND withdrawal))	131
#5	#4 NOT (MH "Neonatal Abstinence Syndrome" OR TX "neonatal abstinence syndrome")	77
#6	#5 NOT (MH "Pregnancy" OR TX pregnant)	54
#7	#6 AND (MH "Drug Withdrawal" OR TX (wean* OR taper* OR "weaning protocol"))	12
#8	#1 AND (MH "Methadone" OR TX methadon*) AND (MH "Substance Withdrawal Syndrome" OR TX withdrawal) AND TX (wean* OR taper* OR protocol*)	23
#9	#3 AND (MH "Morphine" OR MH "Fentanyl" OR TX (morphine OR fentanyl)) AND (MH "Substance Withdrawal Syndrome" OR TX withdrawal)	46
#10	#9 AND TX (wean* OR taper*)	6

Search 6: 54 results → screened based on abstract and titles → 5 already included from PubMed searches* → 0 selected and 49 excluded

Search 7: 12 results → screened based on abstract and titles → 5 already included from PubMed searches* → 0 selected and 7 excluded

Search 8: 23 results → screened based on abstract and title → 4 already included from PubMed searches** → 0 selected and 19 excluded

Search 9: 46 results → screened on the basis of abstract and titles → 5 already included from PubMed searches*** → 0 selected and 41 excluded

Search 10: 6 results → screened based on abstract and title → 3 already included from PubMed searches*** → 0 selected and 3 excluded

* Amirnovin 2018, Bowens 2011, Jeffries 2012, Walters 2021, Steineck 2014

** Amirnovin 2018, Jeffries 2012, Walters 2021, Steineck 2014

*** Bowens 2011, Jeffries 2012, Walters 2021

1.3 Results literature review

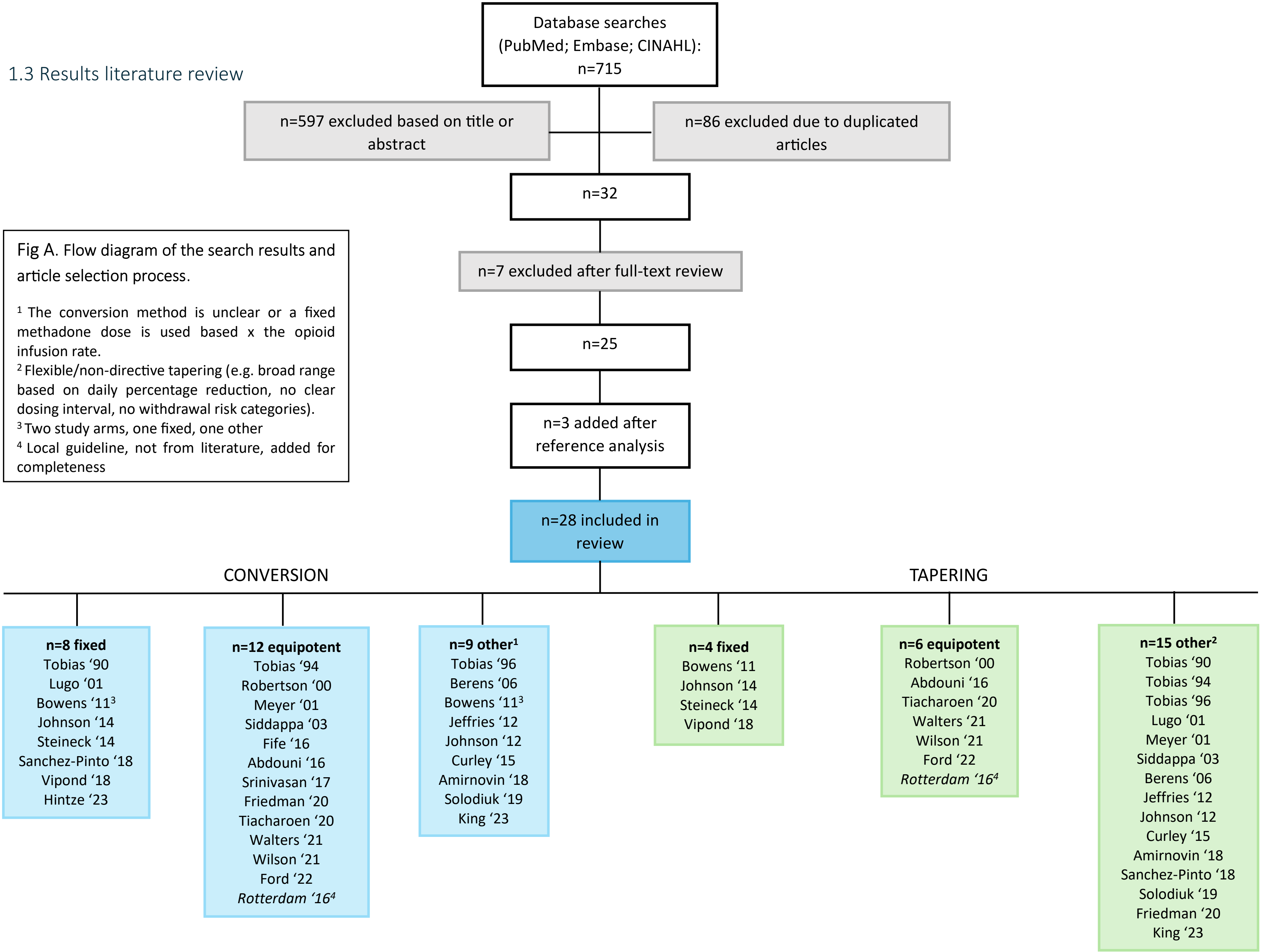

Fig A. Flow diagram of the search results and article selection process.

[1] The conversion method is unclear or a fixed methadone dose is used based x the opioid infusion rate.
[2] Flexible/non-directive tapering (e.g. broad range based on daily percentage reduction, no clear dosing interval, no withdrawal risk categories).
[3] Two study arms, one fixed, one other
[4] Local guideline, not from literature, added for completeness

Table D. Summary of included studies

<table>
<tr><th>Source</th><th>Quality of evidence</th><th>Description of study</th><th>Conclusion for monograph</th></tr>
<tr>
<td>DOI</td>
<td>- Retrospective chart review
- N=42: without guideline, N=134: with guideline
- Level of evidence: B</td>
<td>Impact of a standardized treatment guideline for pediatric Iatrogenic opioid dependence: a quality Improvement initiative
Abdouni R, 2016 (1)

Summary of study results
- Objective of study
To determine whether utilization of a hospital-based clinical practice guideline for the care of pediatric iatrogenic opioid dependence (IOD) would promote a decrease in opioid exposure and improve management of opioid abstinence syndrome (AS).
- Study design
Pediatrics who received continuous opioid infusions for at least 7 days and any length of methadone administration were included. Data on IOD patients from January 2005 to June 2010 was divided into 3 periods: baseline, phase 1, and phase 2. Primary outcome was decrease in opioid exposure, measured by methadone duration of use and any additional opioid bolus doses used in AS management. Documentation of additional opioid bolus doses was regarded as a surrogate measure of AS. Secondary outcomes included total cumulative fentanyl dose, continuous fentanyl infusion duration of use, and hospital and pediatric intensive care unit length of stay.
- Opioid use
This table is an adapted and reformatted version of the data presented in Abdouni et al. (2016), for illustrative purposes only.
<table>
<tr><th></th><th>Baseline Jan 2005 – March 2007 (N=30)</th><th>Phase 1 April 2007 – Sept 2008 (N=48)</th><th>Phase 2 Oct 2008 – June 2010 (N=98)</th></tr>
<tr><td>Total cumulative fentanyl dose (mg/kg)</td><td>2.8 ± 3.7</td><td>1.1 ± 1.1</td><td>0.8 ± 0.7</td></tr>
<tr><td>Continuous fentanyl infusion duration (days)</td><td>12.8 ± 4.8</td><td>11.0 ± 6.1</td><td>11.6 ± 6.2</td></tr>
</table>
</td>
<td>Dose recommendation suggested by this study:-

- Indication: pediatric iatrogenic opioid dependence
- Age/weight (median (IQR)):
▪ Without guideline: 1.9 (0.6-6.4) years
▪ With guideline: 1.1 (0.3-3.6) years
- Dose: mean unknown
- RoA: IV/oral
- Duration of use:
▪ Baseline: 15.3 ± 8.7
▪ Phase 1: 9.5 ± 3.7
▪ Phase 2: 8.1 ± 3.7

Critical appraisal of study results /considerations/remarks
- Fentanyl IV : methadone PO 1 : 2.5
- The study used modified version of the Neonatal Abstinence Scale to evaluate opioid withdrawal symptoms</td>
</tr>
</table>

		- <u>Rationale for methadone dose</u><br>The total fentanyl dose over the previous 24 hours is calculated by combining continuous infusion (dose × body weight × 24, converted to milligrams) and all intermittent doses.<br>The corresponding methadone dose is estimated by multiplying the total fentanyl dose by 2.5.<br>The calculated daily methadone dose is then divided into two equal doses administered every 12 hours.<br>For breakthrough withdrawal symptoms, an additional dose equivalent to 20% of the total daily methadone dose may be administered intravenous.<br>- <u>Taper schedule</u><br>*The tapering schedule is a reformulated text-based version of the protocol described by Abdouni et al. (2016).*<br><u>Opioid exposure ≥7 days</u><br>Step 1: Dose calculation (24h before extubation)<br>Calculate total 24-hour opioid dose (mg)<br>Convert fentanyl to methadone equivalent (multiply by 2.5)<br>Divide total methadone dose into 12-hour interval<br>Step 2: Initiation<br>Start methadone every 12 hours<br>Maintain current fentanyl infusion<br>Step 3: 12 hours later<br>Administer second methadone dose<br>Reduce fentanyl infusion by 50%<br>Step 4: 12 hours later<br>Administer third methadone dose<br>Discontinue fentanyl infusion<br>Step 5: 12 hours later<br>Administer fourth methadone dose<br>Step 6: Tapering phase<br>Begin methadone taper after fifth dose<br>- Exposure 7-14 days (5-day taper): reduce methadone by 20% per day and continue every 12 hours<br>- Exposure 14-30 days (10-day taper): reduce methadone by 10% per day and continue every 12 hours<br>- Exposure >30 (20-day taper): reduce methadone by 10% per day and continue every 12 hours<br>- <u>Conclusions by authors related to efficacy/safety</u><br>There was a significant decrease in methadone duration of use in IOD patients from 15.3 ± 8.7 days at baseline to 9.5 ± 3.7 days during phase 1 ($p = 0.002$), to 8.1 ± 3.7 days on phase 2	

		(reduction not significant, p = 0.106) of this evaluation. Introduction of a standardized, hospital-based clinical practice guideline for children with IOD reduced the length of exposure to opioids and improved opioid AS management.	

DOI	- Prospective pre- and postinterventional study<br>- N=119 (55 pre-intervention; 64 post-intervention)<br>- Level of evidence: B	Implementation of a risk-stratified opioid and benzodiazepine weaning protocol in a pediatric cardiac ICU<br>Amirnovin R, 2018 (2)<br><br>Summary of study results<br>- <u>Objective of study</u><br>To study the effectiveness of a comprehensive, risk-stratified opioid and benzodiazepine weaning protocol at decreasing drug burden without increasing withdrawal symptoms in critically ill children at risk for withdrawal in a pediatric CICU.<br>- <u>Study design</u><br>A pre- and post-intervention design to evaluate a tapering protocol for opioids and benzodiazepines in critically ill children with heart disease. Patients were stratified by withdrawal risk based on drug exposure duration. The protocol included weaning schedules stratified by risk of withdrawal and conversion from continuous to intermittent dosing. After implementation, outcomes from the post-intervention group were compared to those from the pre-intervention group, where no standardized protocol was used.<br>- <u>Opioid use</u><br>*This table is an adapted and reformatted version of the data presented in Amirnovin et al. (2018), for illustrative purposes only.*	Dose recommendation suggested by this study: -<br><br>- **Indication**: opioid and benzodiazepine weaning<br>- **Age/weight**: median (IQR):<br>Pre-intervention: 10 d (2-166)<br>Post-intervention: 10 d (1-170)<br>- **Dose**: mean unknown<br>- **RoA**: IV<br>- **Duration of use**: unknown<br><br>Critical appraisal of study results /considerations/remarks:<br>- The conversion ratios to morphine equivalence used for fentanyl was 0.01:1<br>- The study used the WAT-1 to evaluate opioid withdrawal symptoms

	Pre-intervention (morphine equivalents)	Post-intervention (morphine equivalents)
Daily prewean equivalents (average mg/kg/d per patient), median (IQR)	1.8 (1.4-2.7)	1.7 (1.4-2.2)
Duration of prewean (d), median (IQR)	4 (2-10)	5 (3-8)
Prewean total equivalents (mg/kg/patient), median (IQR)	18.2 (9.1-26.2)	16.4 (8.5-24.6)

		- <u>Rationale for methadone dose</u><br>Methadone dosing can be derived from the fentanyl infusion rate. For infusion rates between 1 and 4 µg/kg/h, the corresponding enteral methadone dose typically ranges from 0.05 to 0.15 mg/kg administered every 8 hours, with higher infusion rates requiring higher methadone doses.<br>- <u>Taper schedule</u><br>*The tapering schedule is a reformulated text-based version of the protocol described by Amirnovin et al. (2018).*<br>Low risk (<5 days)<br>- Discontinue opioid therapy without a structured taper.<br>Moderate risk (5-7 days), wean duration 5 days<br>- Convert continuous opioid infusion to intermittent IV hydromorphone.<br>- Discontinue the opioid infusion at time of conversion.<br>- Start hydromorphone (e.g. 0.1 mg/kg IV every 8 hours).<br>- Gradually taper the total daily opioid dose (e.g. ~20% per day).<br>High risk (8-30 days), wean duration 10 days<br>- Initiate enteral methadone dose.<br>- Continue opioid infusion during initial methadone dosing.<br>- After second methadone dose, reduce opioid infusion by 50%; after third methadone dose, reduce opioid infusion further by 50%.<br>- Continue stepwise reduction of opioid infusion.<br>- Discontinue opioid infusion after sequential methadone dosing.<br>- Taper methadone: reduce every other day by 20%.<br>Very risk (>30 days), wean duration >10 days<br>- Initiate enteral methadone dose.<br>- Continue opioid infusion during initial methadone dosing.<br>- After third methadone dose, reduce opioid infusion by 50%; after sixth methadone dose, reduce opioid infusion further by 50%.<br>- Continue stepwise reduction of opioid infusion.<br>- Discontinue opioid infusion after sequential methadone dosing.<br>- Taper methadone: reduce every other day by 10%.<br>- <u>Conclusions by authors related to efficacy/safety</u><br>Patients in the postintervention period had shorter duration of opioids (19.0 vs 30.0 d; $p < 0.01$). Despite the shorter duration of wean, there was a decrease in withdrawal occurrence (% Withdrawal Assessment Tool score ≥ 4, 4.9% vs 14.1%; $p < 0.01$). There was an 8-day reduction in hospital length of stay (34 vs 42 d; $p < 0.01$.	

This table is an adapted and reformatted version of the data presented in Amirnovin et al. (2018), for illustrative purposes only.

	Pre-intervention (morphine equivalents)	Post-intervention (morphine equivalents)
Total duration of exposure (d), median (IQR)	30 (19-51)	19 (13-28)
Duration of wean phase (d), median (IQR)	23 (15-35)	12 (7-16.5)
Total equivalents (mg/kg/patient), median (IQR)	48.4 (31.2-91.6)	31.2 (21.4-44.2)

<table>
<tr>
<td>DOI</td>
<td>- Prospective, randomized trial, double blind
- N=37
- Level of evidence: B/A2</td>
<td>A prospective evaluation of opioid weaning in opioid-dependent pediatric critical care patients.
Berens RJ, 2006 (3)

Summary of study results
- <u>Objective of study</u>
This study investigated whether opioid-dependent patients could be weaned with oral methadone as effectively in 5 days as in 10 days.
- <u>Study design</u>
Patients were enrolled into the study either while receiving a continuous opioid infusion (preemptive enrollment) or with an increase of NAS after cessation or decrease of opioids (rescue enrollment). Patients who demonstrated active opioid withdrawal symptoms (NAS ≥ 8 every 2 h for 3 consecutive scores) received “rescue therapy” with IV morphine, titrated to reduce the patient’s NAS to < 8 while maintaining a modified Ramsey sedation score > 4, before initiation of the methadone wean protocol. All preemptive enrollment patients were transitioned from continuous fentanyl or morphine to enteral methadone. The methadone was given as a initial “loading dose” every 12 h for 3 consecutive doses titrated for a NAS of ≥ 8 and sedation score ≥ 4. After receiving informed consent, the pharmacist randomized enrolled patients to either a 5-day or 10-day enteral methadone wean protocol.
- <u>Opioid use</u>
This table is an adapted and reformatted version of the data presented in Berens et al. (2006), for illustrative purposes only.
<table>
<tr><td></td><td>5-day wean (N=16)</td><td>10-day wean (N=21)</td></tr>
<tr><td>Total fentanyl dose (mcg/kg)</td><td>1415.7±780.1</td><td>2210.6±1728.9</td></tr>
<tr><td>Fentanyl exposure (days)</td><td>11.38±5.25</td><td>15.54±10.74</td></tr>
</table>
- <u>Taper schedule</u>
The 5-day wean protocol was defined by a 20% reduction of the initial methadone dose each of 5-days, followed by a 5-day course of placebo. The 10-day wean protocol was defined by a 10% reduction of the initial methadone dose each of 10 days with no placebo course.
- <u>Conclusions by authors related to efficacy/safety</u>
This study demonstrated that children at risk for opioid withdrawal can be prospectively safely transitioned to an enteral methadone weaning protocol of either 5 days’ or 10 days’ duration with minimal risk of opioid withdrawal. Although mean NAS scores were slightly higher in the 5-day regimen, the observed scoring was well below the clinically recognized threshold for withdrawal of an NAS score of ≥ 8. The use of a 5-day weaning protocol has the potential to decrease ICU days as well as total hospital days.</td>
<td>Dose recommendation suggested by this study: -

- Indication: treatment and prevention of opioid withdrawal symptoms with oral methadone in the PICU
- Age/weight: (months)
▪ 5-day wean: 9.45 ± 16.69
▪ 10-day wean: 9.80 ± 26.17
- Dose: first methadone dose (mcg/kg)
5-day wean: 235 ± 24
10-day wean: 277 ± 38
- RoA: oral
- Duration of use: unknown

Critical appraisal of study results /considerations/remarks
- Conversion ratio fentanyl : methadone is unknown
- The study used the NAS to evaluate opioid withdrawal symptoms. NAS has been validated in the neonatal population with in utero opioid exposure. Its use in the older iatrogenically exposed population has not been validated by strict scientific criteria</td>
</tr>
</table>

DOI	- Prospective, double-blind, randomized trial<br>- N=60 (weight-based approach – low dose: N=34, formula-based approach – high-dose: N=26<br>- Level of evidence: B	**A trial of methadone tapering schedules in pediatric intensive care unit patients exposed to prolonged sedative infusions.**<br>Bowens CD, 2011 (4)<br><br>Summary of study results<br>- <u>Objective of study</u><br>To compare the efficacy of a low-dose methadone tapering schedule to a high-dose methadone tapering schedule in pediatric intensive care unit patients exposed to infusions of fentanyl, with or without infusions of midazolam, for >5 days.<br>- <u>Study design</u><br>Patients enrolled in the study were randomized to receive one of two starting doses of methadone, a weight-based dose of methadone (low-dose group) or a weight and fentanyl infusion rate-based dose of methadone (high-dose group). Patients were evaluated for symptoms of opioid withdrawal using a withdrawal scale developed for this study. This scale, the Modified Narcotic Withdrawal Scale (MNWS), was modified from the Finnegan Neonatal Abstinence Syndrome monitoring tool, an opioid withdrawal assessment tool that has been validated for neonates. MNWS scores greater than eight were indicative of withdrawal.<br>- <u>Opioid use</u><br>▪ Current fentanyl dose (mcg/kg): 3.0 (2.0-5.0)<br>▪ Peak fentanyl rate (mcg/kg): 4.00 (2.63-6)<br>▪ Total fentanyl dose (mg/kg): 0.58 (0.3-1.1)<br>- <u>Rationale for methadone dose</u><br>The low dose of methadone was chosen based on published reports describing opioid tolerance and its treatment and is the lowest published dose. The high dose of methadone was chosen based on equianalgesic dose conversion and is the highest published dose. For example, a 10-kg patient randomized to the low-dose methadone group would initially receive 1 mg PO methadone per dose regardless of the fentanyl infusion rate. The high dose of methadone was 0.1 mg/kg/dose multiplied by the most recent fentanyl infusion rate (mcg/kg/hr). For example, a 10-kg patient who was receiving 3 g/kg/hr of fentanyl randomized to the high-dose methadone group would initially receive 3 mg PO methadone (0.1 mg/kg x 10 kg x 3). The maximum methadone dose was 20 mg PO.<br>- <u>Taper schedule</u><br>All patients received methadone every 6 hrs for the first 24 hrs, every 12 hrs for the second 24 hrs, and then once daily for the next 10 days. During the 10-day taper, the methadone dose was decreased by 10% per day of the original starting dose until the taper was finished. The fentanyl infusion was weaned off within 72 hrs of receiving the first dose of methadone.<br>- <u>Conclusions by authors related to efficacy/safety</u>	**Dose recommendation suggested by this study:** start low-dose methadone (0.1 mg/kg) and to then titrate upward if the patient experiences excessive withdrawal<br><br>- **Indication**: prevention of opioid withdrawal symptoms in the PICU<br>- **Age/weight**: 4.3 months (1.1-17.8)<br>- **Dose**: mean unknown<br>- **RoA**: oral<br>- **Duration of use**: 12 days<br><br>**Critical appraisal of study results /considerations/remarks**<br>- Fixed-dose methadone (0.1 mg/kg/dose) or 0.1 mg/kg/dose x the most recent fentanyl infusion rate (mcg/kg/hr)<br>- The study used a non-validated pediatric withdrawal scale (modified from the Finnegan NAS)<br>- N=25 failed to completed the assigned methadone taper

		In the low-dose methadone group, 45% of the patients had received a total fentanyl dose > 1.5 mg/kg. In the high-dose methadone group, 51% of the patients had received a total fentanyl dose > 1.5 mg/kg. Patients whose total fentanyl dose was > 1.5 mg/kg were just as likely to complete their assigned methadone taper compared to patients who received ≤ 1.5 mg/kg. The percentage of patients who successfully completed the 10-day methadone taper was the same in the low-dose methadone group as in the high-dose methadone group (56% vs. 62%). The incidence of withdrawal, the number of rescue morphine doses, and the incidence of oversedation were not different between patients who received low-dose methadone and those who received high-dose methadone.<br>- Other considerations<br>Patients that failed to complete the assigned methadone taper had a greater total fentanyl dose and longer pediatric intensive care unit length of stay compared to patients who completed the assigned methadone taper. Patients who had received fentanyl for ≥ 9 days were no more likely to fail the low-dose methadone taper than the high-dose methadone taper.		

<table>
<tr>
<td>DOI</td>
<td>- Unblinded, multicenter, cluster randomized clinical trial
- N=1225 with Sedation Protocol; N=1224 with Usual Care
- Level of evidence: B</td>
<td>Protocolized sedation versus usual care in pediatric patients mechanically ventilated for acute respiratory failure: a randomized clinical trial
Curley MAQ, 2015 (5)

Summary of study results
- <u>Objective of study</u>
To determine whether critically ill children managed with a nurse-implemented, goal-directed sedation protocol experience fewer days of mechanical ventilation than patients receiving usual care.
- <u>Study design</u>
Intervention PICUs (17 sites; n = 1225 patients) used a protocol that included targeted sedation, arousal assessments, extubation readiness testing, sedation adjustment every 8 hours, and sedation weaning. Control PICUs (14 sites; n = 1224 patients) managed sedation per usual care.
- <u>Opioid use</u>
This table is an adapted and reformatted version of the data presented in Curley et al. (2015), for illustrative purposes only.
<table>
<tr><td>Opioid</td><td>Sedation Protocol N=</td><td>Usual Care N=</td></tr>
<tr><td>Morphine</td><td>782</td><td>210</td></tr>
<tr><td>Fentanyl</td><td>431</td><td>989</td></tr>
<tr><td>Opioid* exposure, mean daily dose mg/kg (IQR)</td><td>1.3 (0.7-2.6)</td><td>1.7 (0.8-2.9)</td></tr>
</table>
* Morphine, fentanyl, hydromorphone, remifentanil
- <u>Rationale for methadone dose</u>
For morphine infusions, the methadone dose per administration was calculated as approximately equivalent to one hour of morphine exposure, given every 6 hours.
For fentanyl infusions, the methadone dose was calculated by multiplying the hourly fentanyl infusion rate (mcg/h) by 0.075, administered every 6 hours.
- <u>Taper schedule</u>
The tapering schedule is a reformulated text-based version of the protocol described by Curley et al. (2015).
Start methadone then taper morphine; specifically, decrease the morphine infusion to 50% at the time of the second methadone dose, then discontinue morphine at the time of the third methadone dose.
Taper methadone:
- Continue methadone at the initial dose; every 6 hours for approx. 72 hours.
- Change the frequency to 8 hours for approx. 72 hours.</td>
<td>Dose recommendation suggested by this study:-

- Indication: opioid and benzodiazepine weaning
- Age/weight: median (IQR):
Sedation Protocol: 1.4 years (0.3-7.0)
Usual Care: 2.6 years (0.6-9.2)
- Dose: mean unknown
- RoA: oral/IV
- Duration of use: unknown

Critical appraisal of study results /considerations/remarks:
- The study used the WAT-1 to evaluate opioid withdrawal symptoms
- Morphine IV : methadone oral/IV: 6 : 1</td>
</tr>
</table>

		- If needed, change the frequency to 12 hours for approx. 72 hours.<br>- Gradually decrease methadone dose: reduce to 90% of the initial dose, then progressively in steps (e.g. 80%, 70%, 60%, etc.). Maintain each reduced step for approx. 48 hours.<br>- Continue dose reductions in similar increments until minimal dose is reached.<br>- Stop methadone when the dose is sufficiently low and patient remains clinically stable.<br>- Conclusions by authors related to efficacy/safety<br>There was no difference in the duration of mechanical ventilation for patients managed per sedation protocol (median, 6.5 [IQR: 4.1-11.2]) compared with patients receiving usual care (median, 6.5 [IQR: 3.7-12.1]). Exploratory analyses of several secondary outcomes indicated that the sedation protocol was associated with a difference in patients' sedation experience; patients in the intervention group were able to be safely managed in a more awake and calm state while intubated, receiving fewer days of opioid exposure and fewer sedative classes without an increase in inadequate pain or sedation management or clinically significant iatrogenic withdrawal compared with patients receiving usual care, but they had more days with any report of pain and agitation, suggesting a complex relationship among wakefulness, pain, and agitation.	

DOI	- Retrospective chart review<br>- N=199<br>- Level of evidence: B	Methadone conversion in infants and children: Retrospective cohort study of 199 pediatric inpatients<br>Fife A, 2016 (6)<br><br>Summary of study results<br>- <u>Objective of study</u><br>This study is an attempt to ascertain an equianalgesic dose ratio for methadone in the pediatric population using standard adult dose conversion guidelines.<br>- <u>Study design</u><br>A retrospective chart review was conducted for patients who were converted from their initial opioid therapy regimen (morphine, hydromorphone, and/or fentanyl) to methadone. The primary endpoint was whether or not a dose correction was needed for methadone in the 6 days following conversion using standard dose conversion charts for adults. Documented clinical signs of withdrawal, unrelieved pain, or oversedation were examined.<br>- <u>Rationale for conversion dose</u><br>All opioids were converted to intravenous morphine equivalents, then multiplied the equivalents by three to reach an oral morphine equivalent.<br>- <u>Conclusions by authors related to efficacy/safety</u><br>The majority (53.7 percent) of the 199 children were converted to methadone on intensive care units prior extubation or postextubation. The mean conversion ratio was 23.7 mg of oral morphine to 1 mg of oral methadone (median, 18.8 mg:1 mg, SD=25.7). Most patients experienced an adequate conversion (n=115, 57.8 percent), while 83 (41.7 percent) appeared undermedicated, and one child was oversedated. There were no associations found with conversion ratios for initial morphine dose, days to conversion, or effect of withdrawal of concomitant agents with potential for withdrawal.	Dose recommendation suggested by this study: -<br><br>- **Indication**: patients who underwent conversion from a short-acting opioid to methadone<br>- **Age/weight**:<br>▪ 0-6 months: n=77<br>▪ 0.5-12 years: n=102<br>▪ 13-18 years: n=20<br>- **Dose**: unknown<br>- **RoA**: oral<br>- **Duration of use**: -<br><br>Critical appraisal of study results /considerations/remarks<br>- Morphine oral : methadone oral : 23.7 : 1<br>23.7 oral morphine = 7.9 IV morphine (3:1)<br>- Morphine IV : methadone oral : 7.9 : 1

DOI	- Retrospective cohort study<br>- N=155 (phase 1 baseline preintervention: N=36,<br>phase 2 pharmacist-managed weaning regimen: N=43,<br>phase 3 addition of WAT-1: N=36)<br>- Level of evidence: B	Implementation of an Opioid Weaning Protocol at a Tertiary Care Children's Hospital.<br>Ford J, 2022 (7)<br><br>Summary of study results<br>- <u>Objective of study</u><br>Evaluate the implementation of a pharmacist-managed opioid weaning regimen and objective opioid withdrawal assessment tool in pediatric patients requiring an opioid wean.<br>- <u>Study design</u><br>The study was conducted in 3 phases from chart review of patients admitted from 2012 to 2020: baseline preintervention (not a standardized weaning protocol) (phase 1), pharmacist-managed weaning regimen (phase 2), and addition of an objective assessment tool, the Withdrawal Assessment Tool-1 (twice daily nursing assessments) (phase 3). Withdrawal was defined as a WAT-1 score > 3.<br>Data collection included the total wean duration, total duration of hospital admission, and number of episodes of opioid withdrawal.<br>- <u>Rationale for methadone dose</u><br>Fentanyl dose (mcg/kg/hr) x weight (kg) x 0.08 (conversion factor) = ‘’x’’ mg of methadone q6h.<br>Morphine dose (mg/kg/hr) x weight (kg) x 1 (conversion factor) = ‘’x’’ mg of methadone q6h.<br>- <u>Taper schedule</u><br>Three standardized weans were developed with wean selection dependent on the duration of opioid exposure: low risk with < 15 days of opioid infusion, moderate risk with 16 to 30, and high risk with ≥ 31 days. The pharmacist managed wean protocol used in the current study was derived from the protocol described by Steineck et al. (2014) with a few revisions.<br>One such revision included avoiding opioid tapering when children had received < 5 days of opioids. In this case, opioid sparing therapies such as a clonidine taper or as needed opioid administrations may have been used.<br>*The tapering schedule is a reformulated text-based version of the protocol described by Ford et al. (2022).*<br>Transition from opioid infusion to schedules methadone:<br>after second methadone dose, decrease opioid infusion by 50% of the original opioid infusion;<br>after third methadone dose, decrease opioid infusion by 75% of the original opioid infusion;<br>after fourth methadone dose, discontinue opioid infusion.<br>Low-risk wean (opioid duration ≤15 days)<br>- Initial methadone dose ‘’x’’ mg x 1.3 for bioavailability of 70–80%.<br>- Taper methadone:<br>- X mg q6h 48h<br>- X mg q8h 48h	Dose recommendation suggested by this study: -<br><br>- **Indication**: prevention of opioid withdrawal symptoms in the PICU<br>- **Age/weight**: (years)<br>▪ Phase 1: 0.48 (0.15-2.44)<br>▪ Phase 2: 0.46 (0.16-1.71)<br>▪ Phase 3: 0.84 (0.20-2.61)<br>- **Dose**: mean unknown<br>- **RoA**: oral<br>- **Duration of use**: (days)<br>▪ Phase 1: 13.4 (9.81-19.8)<br>▪ Phase 2: 11.4 (6.86-22.9)<br>▪ Phase 3: 9.23 (7.84-13.1)<br><br>Critical appraisal of study results /considerations/remarks<br>- Morphine IV : methadone oral 4.6 : 1 (daily dose)<br>- Fentanyl IV : methadone oral 1 : 17.3 (daily dose)<br>- The study used the WAT-1 to evaluate opioid withdrawal symptoms

		- X mg q12h 48h<br>- X mg q24h 48h<br>Moderate-risk wean (opioid duration 16-30 days)<br>- Initial methadone dose ''x'' mg x 1.3 for bioavailability of 70–80%.<br>- Taper methadone:<br>- X mg q6h 48h<br>- 0.85*X mg q6h 48h<br>- 0.85*X mg q8h 48h<br>- 0.7*X mg q8h 48h<br>- 0.6*X mg q8h 48h<br>- 0.45*X mg q8h 48h<br>- 0.45*X mg q12h 48h<br>- 0.3*X mg q12h 48h<br>- 0.3*X mg q24h 48h<br>High-risk wean (opiod duration ≥31 days)<br>- Initial methadone dose ''x'' mg x 1.3 for bioavailability of 70–80%.<br>- Taper methadone:<br>- X mg q6h 48h<br>- 0.9*X mg q6h 48h<br>- 0.8*X mg q6h 48h<br>- 0.7*X mg q6h 48h<br>- 0.7*X mg q8h 48h<br>- 0.6*X mg q8h 48h<br>- 0.5*X mg q8h 48h<br>- 0.4*X mg q8h 48h<br>- 0.4*X mg q12h 48h<br>- 0.3*X mg q12h 48h<br>- 0.2*X mg q12h 48h<br>- 0.1*X mg q12h 48h<br>- 0.1*X mg q24h 48h<br>- Conclusions by authors related to efficacy/safety<br>Length of stay, wean duration, and episodes of withdrawal with need for rescue dose all decreased significantly from phase 1 to phase 3. Despite a more rapid opioid wean duration, documented withdrawal declined dramatically from 55.6% of all children having withdrawal in phase 1 to only 16.7% of all children having withdrawal in phase 3.	

DOI	- Retrospective cohort study<br>- N=51 (total cohort: N=70; 16 without data on methadone)<br>- Level of evidence: B	Methadone's Effect on Cardiac Repolarization: Safety in the PICU<br>Friedman SD 2020 (8)<br><br>Summary of study results<br>- Objective of study<br>This study aimed to describe the safety of methadone use in the PICU by assessing its effect on the corrected QT interval (QTc), with the hypothesis that methadone does not cause a significant change in QTc from baseline.<br>- Study design<br>All patients ≤18 years old who were initiated on methadone during PICU admission (September 2016–July 2017) for prevention or treatment of opioid withdrawal were identified. QTc intervals were retrospectively measured at baseline, 12 hours after the first dose, at steady state (96 hours), 12 hours after the highest dose, and prior to PICU discharge.<br>- Rationale for methadone dose<br>Within the institution, a standard conversion method for fentanyl infusions to methadone was used, although practice variation among providers existed. The standard approach was to determine the hourly fentanyl dose (mcg/hr) and use a conversion factor of 30 to obtain the IV methadone dose which is then converted to milligrams. IV methadone was considered two times as potent as enteral methadone and all numbers were reported as milligrams per day of IV methadone The starting dosage of methadone used in this study was based on the calculation published by Berens et al.<br>- Taper schedule<br>- No formal methadone tapering schedule was described because tapering efficacy was not a study objective.<br>- Patients were typically given an initial loading phase with methadone every 12 hours for three doses and then transitioned to once-nightly dosing.<br>- Some subjects remained on every-12-hour dosing.<br>- Conclusions by authors related to efficacy/safety<br>Methadone did not significantly prolong QTc intervals at 12 hours, steady state (96 hours), or PICU discharge compared with baseline. Two subjects (5.6%) had a QTc increase ≥40 ms at steady state; both had structural heart disease. No correlation was found between methadone dose and change in QTc interval. No subjects experienced torsades de pointes during the study period. The authors concluded that methadone can be safely used in critically ill children and does not significantly alter cardiac repolarization, although caution is warranted in patients with structural heart disease.	Dose recommendation suggested by this study: -<br><br>- **Indication**: prevention or treatment of opioid withdrawal in PICU patients<br>- **Age/weight**: median 3 months (IQR 2-24)<br>- **Dose**: starting dose: mean (SD) 0.23 (0.17) mg/kg/day; mean (SD) 0.28 (0.22) mg/kg/day (highest dose)<br>- **RoA**: IV<br>- **Duration of use**: 11-month period<br><br>Critical appraisal of study results /considerations/remarks<br>- Fentanyl IV : methadone IV: 1 : 30 (= Fentanyl IV : methadone oral 1 : 5 daily dose)<br>- No standardized withdrawal assessment tool was used

<table>
<tr>
<td>DOI</td>
<td>- Retrospective study
- N=67
- Level of evidence: B</td>
<td>
Characterization of early versus late opioid iatrogenic withdrawal syndrome in critically ill children transitioning from fentanyl infusions to methadone
Hintze TD, 2023 (9)

Summary of study results
- <u>Objective of study</u>
The purpose was to compare clinical manifestations among patients who developed opioid IWS within 24 hours (early) versus ≥24 hours (late) of fentanyl discontinuation when enteral methadone was initiated.
- <u>Study design</u>
Participants were children who received fentanyl infusions for ≥3 days and initiated on methadone prior to fentanyl discontinuation. The primary objective was to compare clinical characteristics between those with early (within 24 hours of discontinuation of fentanyl infusion) versus late (≥ 24 hours within 24 hours of discontinuation of fentanyl infusion) opioid IWS. Opioid IWS was defined as a Withdrawal Assessment Tool-1 score ≥3 within 5 days of fentanyl discontinuation. Secondary objectives included a comparison of time to IWS, clinical characteristics, and risk factors among patients with and without IWS.
- <u>Opioid use</u>
This table is an adapted and reformatted version of the data presented in Hintze et al. (2023), for illustrative purposes only.
<table>
<tr><td>Median (IQR)</td><td>Overall (N=67)</td><td>Opiod IWS (N=50)</td><td>No opioid IWS (N=17)</td></tr>
<tr><td>Initial rate (mcg/kg/h)</td><td>(1.0-2.0)</td><td>2.0 (1.0-2.0)</td><td>2.0 (1.0-2.0)</td></tr>
<tr><td>Peak rate (mcg/kg/h)</td><td>3.0 (2.5-3.0)</td><td>4.0 (3.0-4.0)</td><td>3.0 (2.0-3.0)</td></tr>
<tr><td>Infusion duration (h)</td><td>193.0 (139.0-193.0)</td><td>192.0 (139.0-192.0)</td><td>207.0 (158.0-207.0)</td></tr>
<tr><td>Cumulative exposure (mcg/kg)</td><td>72.5 (44.0-72.5)</td><td>78.5 (39.138.1)</td><td>65.3 (45.9-85.9)</td></tr>
</table>
- <u>Conclusions by authors related to efficacy/safety</u>
50 children developed opioid IWS within a median time of 3.5 hours despite the fact that enteral methadone was overlapped with fentanyl for a median of approximately 2 days. No differences were noted for those with and without IWS. 37 patients with IWS developed early IWS. A higher percentage of males in the late versus early group developed IWS, 100 percent versus 51.4 percent. The median overlap time with methadone and fentanyl was shorter in the early versus late IWS group, 27.5 versus 64.0 hours.
</td>
<td>
Dose recommendation suggested by this study: -

- Indication: prevention of opioid withdrawal symptoms in the PICU
- Age/weight: median (IQR): 0.8 years (0.5-1.0), 6.6 kg (4.4-10.3)
- Dose: 0.1 mg/kg every 6 hours
- RoA: oral
- Duration of use: -

Critical appraisal of study results /considerations/remarks
- Fixed-dose methadone
- The study used the WAT-1 to evaluate opioid withdrawal symptoms
</td>
</tr>
</table>

<table>
<tr>
<td>DOI</td>
<td>- Retrospective analysis
- N=43
- Level of evidence: B</td>
<td>Use of methadone for prevention of opioid withdrawal in critically ill children.
Jeffries SA, 2012 (10)

Summary of study results
- Objective of study
This study aimed to describe the institution’s experience with methadone for weaning opioid-dependent children in the PICU.
- Study design
The primary objectives were to determine if opioids were tapered according to the protocol and to determine the conversion ratio for IV morphine to oral methadone that was used. Secondary objectives were to describe the methadone dosage used and the clinical outcomes, to evaluate adjustments to methadone dosing, and to report the incidence of adverse effects. Validated scoring systems (the Withdrawal Assessment Tool and the State Behavioral Scale) were used to assess symptoms of withdrawal and degree of sedation, respectively. Patients with a WAT-1 score of 3 or above are considered to be experiencing withdrawal symptoms.
- Opioid use
Median amount of morphine received in the 24 h preceding conversion was 0.75 mg/kg (range 0.2 – 2.82 mg/kg).
This table is an adapted and reformatted version of the data presented in Jeffries et al. (2012), for illustrative purposes only.
<table>
<tr><th>Patient group</th><th>Opioid dose (mg morphine equivalents/kg/day)</th><th>Morphine:methadone conversion ratio</th></tr>
<tr><td>5-day weaning (N= 32)</td><td>0.79 (0.32–2.29)</td><td>1:0.78 (0.32–1.24)</td></tr>
<tr><td>10-day weaning (N=11)</td><td>0.71 (0.43–1.19)</td><td>1:0.98 (0.38–1.68)</td></tr>
<tr><td>All patients (N=43)</td><td>0.77 (0.32–2.29)</td><td>1:0.84 (0.32–1.68)</td></tr>
</table>
- Taper schedule
According to the protocol, patients are to be weaned with tapering over 5 days (20% of original dose per day) or 10 days (10% of original dose per day), depending on the duration of continuous morphine infusion.
- Conclusions by authors related to efficacy/safety
For 31 patients (72%), the protocol was not used, and there were no patients for whom the protocol was followed to completion. The median duration of weaning was 10 days (range 0–91 days). During the first 10 days of weaning, 18 patients (42%) experienced withdrawal symptoms. The methadone dose was increased for 11 (26%) of the 43 patients.</td>
<td>Dose recommendation suggested by this study:-

- Indication: prevention of opioid withdrawal symptoms in the PICU
- Age/weight: 0.25 – 201 months (median: 8)
- Dose: mean unknown
- RoA: oral
- Duration of use: (median)
▪ 5-day weaning: 9 days (0-48)
▪ 10-day weaning: 15 days (7- 91)

Critical appraisal of study results /considerations/remarks
- Morphine IV : methadone oral 1 : 0.78 (5-day weaning) 1 : 0.98 (10-day weaning) (daily dose)
- The study used the WAT-1 to evaluate opioid withdrawal symptoms
- The methadone protocol was not followed to completion for any patient. As such, the efficacy and safety of the protocol could not be evaluated
- Many patients experienced withdrawal symptoms, and the methadone dosage was increased in more than half of the patients. A 1:1 conversion ratio was likely too low</td>
</tr>
</table>

		Patients were sedated for a median of 1 day (range 0–9 days), were comfortable for a median of 6.5 days (range 1–64 days), and were agitated for a median of 2.5 days (range 0–23 days). Naloxone was required for 2 patients.	

DOI	- Retrospective descriptive study<br>- N=55<br>- Level of evidence: B	Evaluation of initial methadone dosing for prevention of iatrogenic opioid abstinence syndrome in children<br>Johnson PN 2012 (11)<br><br>Summary of study results<br>- Objective of study<br>To determine the initial methadone dosing utilized (mg/kg/day) for prevention of iatrogenic opioid abstinence syndrome (IOAS) and the impact of the initial dose on the number of dose changes required within 72 hours following discontinuation of opioid continuous infusion.<br>- Study design<br>Patients <18 years who received enteral methadone for prevention of IOAS between January and December 2008 were included. Patients receiving methadone for treatment of withdrawal, chronic pain, palliative care, or neonatal abstinence syndrome were excluded.<br>- Opioid use<br>Mean cumulative fentanyl exposure:<br>• Low-dose methadone group: 516.6 ± 301.4 µg/kg<br>• High-dose methadone group: 1752 ± 1365.8 µg/kg<br>Mean duration of fentanyl infusion:<br>• Low-dose group: 252.5 ± 172.8 hours<br>• High-dose group: 393.8 ± 331.4 hours<br>- Rationale for methadone dose<br>No standardized methadone conversion was used at the institution; initial dosing depended on clinician discretion.<br>- Taper schedule<br>No standardized methadone protocol was used in the institution. Most patients had methadone dose reductions of approximately 10–20% of the initial dose every 24–48 hours. The reduction amount and interval were prescriber dependent.<br>- Conclusions by authors related to efficacy/safety<br>The initial methadone dosage regimens varied greatly, and approximately half of the patients required a dose adjustment. No significant differences were found between low- and high-dose groups. The authors concluded that there is no "one-size-fits-all" approach for determining the initial methadone dose and recommended the use of a standardized withdrawal monitoring tool and dosing protocol.	Dose recommendation suggested by this study: -<br><br>- **Indication**: prevention of iatrogenic opioid abstinence syndrome (IOAS)<br>- **Age/weight**: median age: 0.5 years (range 0.03–12.2)<br>- **Dose**: low-dose group (<0.84 mg/kg/day): n=27; high-dose group (≥0.84 mg/kg/day): n=28<br>- **RoA**: oral<br>- **Duration of use**:<br>Mean duration of taper:<br>• Low-dose group: 14.7 ± 9.2 days<br>• High-dose group: 26.2 ± 11.5 days (P<0.01)<br><br>Critical appraisal of study results /considerations/remarks<br>- No standardized methadone conversion/protocol was used<br>- No standardized withdrawal assessment tool was used

<table>
<tr>
<td>DOI</td>
<td>- Prospective, single-centered, interventional study
- N=35 pharmacist-managed patients (prospective), N=28, physician-managed patients (retrospective)
- Level of evidence: B</td>
<td>Development and implementation of a pharmacist-managed, neonatal and pediatric, opioid-weaning protocol
Johnson MR, 2014 (12)

Summary of study results
- <u>Objective of study</u>
To compare the length of wean and abstinence severity in neonatal and pediatric patients with neonatal abstinence syndrome or iatrogenic opioid dependence treated with a pharmacist-managed, methadone-based protocol compared with physician-managed patients treated with either methadone or dilute tincture of opium (DTO).
- <u>Study design</u>
Wean duration and severity of neonatal abstinence syndrome were compared between groups using the Student *t* test. Patients with 5 days or less continuous opioid exposure or neonates with in utero opioid exposure were monitored for abstinence syndrome, and methadone was initiated if a patient exhibited withdrawal signs and symptoms with the previously defined scores. Patients with more than 5 days of continuous opioid exposure were empirically started on methadone.
- <u>Opioid use</u>
This table is an adapted and reformatted version of the data presented in Johnson et al. (2014), for illustrative purposes only.
<table>
<tr><td></td><td>RetroMeth</td><td>ProsMeth</td></tr>
<tr><td>Fentanyl rate (mcg/kg/min)</td><td>3.3 ± 1.55</td><td>3.13 ± 1.75</td></tr>
<tr><td>Fentanyl duration (days)</td><td>10.04 ± 3.95</td><td>11.37 ± 6.81</td></tr>
</table>
RetroMeth: retrospective study of physician-managed patients treated with methadone; ProsMeth: prospective study of patients in the pharmacist-managed
- <u>Rationale for methadone dose</u>
The initial dose of methadone was determined per protocol and was given orally every 6 or 8 hours for 48 hours. The maximum initial methadone dose for pediatric patients was 10 mg. The smaller dosage (0.1 mg/kg/dose) was based on current recommendations, and the larger dosage (0.2 mg/kg/dose) was based on clinical experience and a retrospective study (Siddappa 2003) showing that larger methadone doses may improve control of withdrawal symptoms without adverse drug reactions, although that dosage was based on a specific fentanyl conversion. The methadone interval was lengthened throughout weaning as follows: every 6 hours (if applicable), to every 8 hours, to every 12 hours, to every 24 hours. Weans consisted of 10% to 20% decreases in the total daily dose of methadone and were a fixed amount based on the patient's initial methadone dose or the largest dose required.</td>
<td>Dose recommendation suggested by this study:-

- Indication: neonatal abstinence syndrome or iatrogenic opioid dependence
- Age/weight (mean ± SD):
▪ ProsMeth: 38.49 ± 55.49 months
▪ RetroMeth: 33.22 ± 54.47 months
- Dose: mean unknown
- RoA: oral
- Duration of use: unknown

Critical appraisal of study results /considerations/remarks
- Fixed-dose methadone
- The study used the Finnegan Neonatal Abstinence Scoring tool < 6 months and the OBWS ≥ 6 months to evaluate opioid withdrawal symptoms</td>
</tr>
</table>

- <u>Taper schedule</u>
  The tapering schedule is a reformulated text-based version of the protocol described by Johnson et al. (2014).
  Iatrogenic exposure (72 hours to 5 days) with withdrawal signs:
  - Initiate methadone 0.1 mg/kg/dose orally every 6-8 hours
  - Taper methadone by 20%
  Iatrogenic exposure (>5 days):
  - Initiate methadone 0.2 mg/kg/dose orally every 6-8 hours
  - Taper methadone by 10%
- <u>Conclusions by authors related to efficacy/safety</u>
  This table is an adapted and reformatted version of the data presented in Johnson et al. (2014), for illustrative purposes only.

Protocol type	Mean duration (days) ± SD	95% CI (days)
RetroMeth (N=28)	9.82 ± 5.68	7.72–11.92
RetroOpium (N=5)	14.0 ± 5.39	2.98–18.72
ProsMeth (N=35)	8.69 ± 2.83	7.75–9.63

Use of a pharmacist-managed, methadone-based weaning protocol standardizes patient care and has the potential to decrease abstinence severity and shorten duration of wean versus physician-managed patients exposed to opioids in utero. Additionally, a methadone wean of 10% to 20% per day was well tolerated in both neonatal and pediatric patients.

<table>
<tr>
<td>DOI</td>
<td>- Prospective study
- Pre-Intervention N=46; Post-Intervention N=48
- Level of evidence: B</td>
<td>Sedation weaning initiative targeting methadone exposure: single center improvements in withdrawal symptoms and hospital length of stay for pediatric cardiac critical care
King CE, 2023

Summary of study results
- <u>Objective of study</u>
To decrease the average days of methadone exposure to within goal for moderate- and high-risk patients within 6 months.
- <u>Study design</u>
Sedation weaning guidelines were implemented over the course of 12 months. Data were tracked every 6 months and compared with the 12 months pre-intervention. Patients were stratified into low, moderate, and high risk withdrawal categories based on duration of opioid infusion exposure.
- <u>Opioid use</u>
This table is an adapted and reformatted version of the data presented in King et al. (2023), for illustrative purposes only.
<table>
<tr><td>Opioid</td><td>Pre N=</td><td>Post N=</td></tr>
<tr><td>Morphine</td><td>9</td><td>4</td></tr>
<tr><td>Fentanyl</td><td>46</td><td>48</td></tr>
</table>
- <u>Taper schedule</u>
The tapering schedule is a reformulated text-based version of the protocol described by King et al. (2023).
Risk stratification was based on the duration of continuous opioid infusion:
- Low risk: <5 days
- Moderate risk: 5–7 days
- High risk: >8 days
Methadone was initiated 24–48 hours prior to extubation in patients with an opioid infusion of ≥5 days. Dosing was determined using an opioid-to-methadone conversion approach.
Opioid weaning was initiated before extubation by reducing the infusion by approximately 50% after the third and fourth methadone doses.
Rescue doses of morphine were administered when the WAT-1 score was ≥4.
Methadone tapering was performed by reducing the dose by approximately 20% every 24–48 hours, provided fewer than three rescue doses were required within a 24-hour period.
Target weaning duration depended on risk category:
- Low risk: immediate discontinuation (0 days)
- Moderate risk: 5–7 days</td>
<td>Dose recommendation suggested by this study: -

- Indication: sedation medicating weaning in a ICU
- Age/weight: mean (SD):
Pre-Intervention: 48 days (77)
Post-Intervention: 55 days (78)
- Dose: mode dose: 0.05 mg/kg every 8 hours pre-intervention and 0.1 mg/kg every 6 hours post-intervention
- RoA: unknown
- Duration of use: mean (SD):
Pre-Intervention: 30.1 (24)
Post-Intervention: 19.9 (13)

Critical appraisal of study results /considerations/remarks:
- The study used the WAT-1 to evaluate opioid withdrawal symptoms</td>
</tr>
</table>

		- High risk: 10–14 days<br>- Conclusions by authors related to efficacy/safety<br>This study observed decreased dexmedetomidine infusion duration, decreased methadone wean duration, decreased frequency of elevated WAT-1 scores, and decreased hospital length of stay post-intervention. For the primary aim, methadone wean duration consistently decreased after each study period.	

<table>
<tr>
<td>DOI</td>
<td>- Retrospective analysis
- N=22
- Level of evidence: B</td>
<td>Enteral methadone to expedite fentanyl discontinuation and prevent opioid abstinence syndrome in the PICU.
Lugo RA, 2001 (13)

Summary of study results
- <u>Objective of study</u>
To determine if enterally administered methadone can facilitate fentanyl discontinuation and prevent withdrawal in children at high risk for opioid abstinence syndrome.
- <u>Study design</u>
Development of opioid abstinence syndrome was evaluated during fentanyl infusion for ≥ 9 days and methadone dosage reductions and for 72 hours thereafter.
- <u>Opioid use</u>
Fentanyl infusion rate on starting the taper was 5.0 ± 3.6 mcg/kg/hr.
This table is an adapted and reformatted version of the data presented in Lugo et al. (2001), for illustrative purposes only.
<table>
<tr><td>Parameter</td><td>Value</td></tr>
<tr><td>Duration of infusion (days), mean ± SD</td><td>17.8 ± 8.4</td></tr>
<tr><td>Peak infusion rate (μg/kg/h), mean ± SD</td><td>5.9 ± 3.8</td></tr>
<tr><td>Total cumulative dose (μg/kg), median (range)</td><td>1302 (354–7535)</td></tr>
<tr><td>Time to discontinuation (days), median (range)</td><td>2.6 (0–11.9)</td></tr>
</table>
- <u>Taper schedule</u>
After methadone is begun, the dosage of fentanyl is reduced 10–15% of the original dose every 8 hours until the infusion is discontinued in 2–3 days. Physicians are permitted to taper the fentanyl dosage more rapidly or slowly as required by the clinical situation. If signs or symptoms of withdrawal occur during the taper, the taper is suspended, an extra dose of fentanyl or methadone is administered, the maintenance dosage of methadone is increased by 50%, and the fentanyl taper is resumed 24 hours later. Two days after fentanyl is discontinued, the dosage of methadone is tapered by increasing the interval to every 8 hours for 2 days, then every 12 hours for 2 days, followed by 25% reductions of the original dose every 2–3 days until methadone is discontinued.
- <u>Conclusions by authors related to efficacy/safety</u>
Methadone was begun 1.6 ± 1.9 days before tapering fentanyl. Fentanyl was tapered and discontinued in a median of 2.6 days (range 0–11.9 days). One patient experienced significant</td>
<td>Dose recommendation suggested by this study: fixed dose (0.1/mg/kg every 6 hours)

- Indication: prevention of opioid withdrawal in children at high risk for opioid abstinence syndrome in the PICU
- Age/weight: 6.1 ± 5.4 years, 22.0 ± 18.6 kg
- Dose: 0.1 mg/kg every 6 hours (0.50 ± 0.22 mg/kg/day most commonly used in four divided doses)
- RoA: NG tube
- Duration of use: 18.2 ± 11.9 days

Critical appraisal of study results /considerations/remarks
- Fixed-dose methadone</td>
</tr>
</table>

		opioid withdrawal after fentanyl discontinuation, which resolved after reinstitution of fentanyl and increasing the dosage of methadone to 0.3 mg/kg every 6 hours.<br>- <u>Other considerations</u><br>The study recommend against using an "equivalent" dosage of methadone to treat or prevent withdrawal since this pharmacokinetic approach usually results in methadone dosages that are unnecessarily high for this indication. Furthermore, the half-lives of fentanyl and methadone in such calculations must be specific for a critically ill pediatric population, otherwise the dosage conversion is likely to be erroneous. For example, a dosage conversion using a fentanyl:methadone half-life ratio of 1:50–100 (based on a 30-minute fentanyl half-life) and a 100:1 fentanyl:methadone potency ratio was recommended. Although these proposed ratios result in a relatively low and reasonable calculated dosage of methadone for prevention of withdrawal, more accurate estimates of fentanyl and methadone half-lives in critically ill children result in a calculated equivalent dosage of methadone that is very large. For example, in perioperative children, methadone's half-life was 19 hours, and in critically ill children, fentanyl's half-life after prolonged infusion was 21.1 hours, which represents a 1:1 ratio of half-lives. Thus, if the daily dose of fentanyl is multiplied by 100 to account for the potency difference and divided by approximately 1 to account for the difference in half-lives, one arrives at an "equivalent" dosage of methadone (assuming similar distribution volumes) that greatly exceeds dosages necessary to prevent opioid withdrawal. This likely would result in prolonged opioid dependence and a longer opioid taper.	

DOI	- Prospective study<br>- N= 29<br>- Level of evidence: B	Efficacy of an enteral 10-day methadone wean to prevent opioid withdrawal in fentanyl-tolerant pediatric intensive care unit patients.<br>Meyer MM, 2001 (14)<br><br>Summary of study results<br>- <u>Objective of study</u><br>To demonstrate the efficacy of a 10-day, single daily dose, enteral methadone weaning protocol for the preventions of opioid withdrawal in pediatric patients after prolonged fentanyl exposure.<br>- <u>Study design</u><br>The signs and symptoms of opioid withdrawal are assessed by the nursing staff by using the Neonatal Abstinence Score (NAS) designed by Finnegan. The risk of opioid withdrawal is determined by a persistently elevated NAS (NAS ≥ 8 for three consecutive scores), a recent decrease or discontinuation of the fentanyl infusion (within 24–48 hrs before onset of symptoms), fentanyl infusion duration of ≥ 5 days, or a cumulative fentanyl dose of ≥ 1.5 mg/kg. If these criteria are met, methadone therapy is initiated.<br>- <u>Opioid use</u><br>- Duration of exposure: 14.5 ± 9.2 days<br>- Cumulative dose: 1.93 ± 1.53 mg/kg<br>- Peak fentanyl infusion: 9.6 ± 4.3 mcg/kg/hr<br>- <u>Rationale for methadone dose</u><br>Conversion from intravenous fentanyl to enteral methadone was performed using a stepwise approach based on opioid equivalence.<br>For example, in a child weighing 5 kg receiving a fentanyl infusion of 5 µg/kg/h, the total fentanyl dose corresponds to 25 µg per hour. This hourly fentanyl dose was converted to an equivalent morphine dose using an approximate potency ratio of 60:1 (morphine:fentanyl). Accordingly, the equivalent morphine dose was approximately 1.5 mg every 4 hours.<br>The intravenous morphine dose was subsequently converted to enteral methadone by applying a conversion factor of approximately 2, resulting in a methadone dose of about 3 mg administered enterally.<br>- <u>Taper schedule</u><br>Methadone was administered every 12 hours for the initial doses, after which tapering was initiated by gradually reducing the daily methadone dose by approximately 10%.<br>- <u>Conclusions by authors related to efficacy/safety</u><br>Twenty-five patients successfully completed the 10-day methadone weaning protocol without developing opioid withdrawal symptoms. Withdrawal complications that required a weaning delay were seen in three patients, and one patient expired before completion. Sixteen patients were	Dose recommendation suggested by this study:-<br><br>- **Indication**: prevention of opioid withdrawal in pediatric patients after prolonged fentanyl exposure in the PICU<br>- **Age/weight**: 1 day – 19.8 years (mean: 25.6 ± 54.4)<br>- **Dose**: mean unknown<br>- **RoA**: oral<br>- **Duration of use**: 10 days<br><br>Critical appraisal of study results /considerations/remarks<br>- Morphine IV : methadone oral 1.5 : 1 (daily dose)<br>- Fentanyl IV : methadone oral 1 : 10* (daily dose)<br>* The fentanyl dose was first converted to morphine (1 : 6) and then to methadone)<br>- The study used the NAS to evaluate opioid withdrawal symptoms. NAS has been validated in the neonatal population with in utero opioid exposure. Its use in the older iatrogenically exposed population has not been validated by strict scientific criteria

		discharged to complete their weaning schedule at home without incident.<br>There were no episodes of oversedation, desaturation, or hypotension during the methadone therapy period.	

DOI	- Prospective study<br>- N=20<br>- Level of evidence: B	Evaluation of an opiate-weaning protocol using methadone in pediatric intensive care unit patients.<br>Robertson RC, 2000 (15)<br><br>Summary of study results<br>- <u>Objective of study</u><br>To evaluate the efficacy of a standardized opiate-weaning protocol using methadone compared with methadone weaning before protocol development.<br>- <u>Study design</u><br>10 children who received methadone for weaning from continuous opiate infusions for ≥ 7 days compared with 10 patients undergoing weaning by standardized protocol. A standardized opiate-weaning protocol using methadone was developed by an interdisciplinary quality improvement team consisting of a pharmacist (PharmD), pharmacy resident, three critical care physicians, and a critical care nurse. The protocol addresses weaning patients who have been on a continuous opiate infusion of fentanyl or morphine for ≥ 7 days using methadone as a substitute for the continuous infusion. Demographic characteristics, length of opiate use, length of methadone wean, and presence of withdrawal complications were compared between protocol (P) and nonprotocol groups (NP).<br>- <u>Opioid use</u><br>NP: n=9 fentanyl, n=1 morphine<br>P: n=7 fentanyl, n=3 morphine<br>- <u>Rationale for methadone dose</u><br>The hourly morphine or fentanyl dose is converted to an equipotent dose of methadone given every 6 hrs. On the second day of the protocol weaning process, methadone is converted from intravenous to enteral form unless contradicted by patient status.<br>- <u>Taper schedule</u><br>With continuous opiate use for 7–14 days (short-term use), the daily methadone dose is decreased by 20% daily, producing a total weaning time of 5 days. Opiate use for >14 days (long-term use) is weaned by decreasing the daily methadone dose by 20% every other day, for a total expected weaning time of 10 days.<br>*The tapering schedule is a reformulated text-based version of the protocol described by Robertson et al. (2000).*<br>Opioid duration 7-14 days<br>- Use current 1-hr of the opioid to convert to an equipotent dose of methadone; Morphine IV : methadone IV/oral: 6 : 1 (daily dose); Fentanyl IV : methadone IV: 1:16.7 (daily dose).<br>- Day 1: Administer methadone intravenously every 6 hours for 24 hours.<br>- Day 2: Reduce the original opioid infusion by approximately 20% and continue methadone orally every 8 hours.	Dose recommendation suggested by this study:-<br><br>- **Indication**: prevention of opioid withdrawal in the PICU<br>- **Age/weight**: 3.5 months – 15 years<br>▪ NP: 14.5 months (median)<br>▪ P: 17 months (median)<br>- **Dose**: mean unknown<br>- **RoA**: IV and oral<br>- **Duration of use**: 5 or 10 days<br><br>Critical appraisal of study results /considerations/remarks<br>- Morphine IV : methadone IV/oral 6 : 1 (daily dose)<br>- Fentanyl IV : methadone IV 1 : 16.7 (daily dose)<br>After day 1, IV methadone was converted to oral methadone<br>- IV and oral methadone were used in this population/protocol<br>- No scoring system was used in the study to evaluate opioid withdrawal symptoms

		- Day 3: Reduce the original opioid infusion by approximately 20% and continue methadone orally every 8 hours.<br>- Day 4: Reduce the original opioid infusion by approximately 20% and continue methadone orally every 12 hours.<br>- Day 5: Reduce the original opioid infusion by approximately 20% and continue methadone orally every 24 hours.<br>- Day 6: Discontinue methadone<br>Opioid duration >14 days<br>- Use current 1-hr of the opioid to convert to an equipotent dose of methadone; Morphine IV : methadone IV/oral: 6 : 1 (daily dose); Fentanyl IV : methadone IV: 1:16.7 (daily dose).<br>- Day 1: Administer methadone intravenously every 6 hours for 24 hours.<br>- Day 2: Continue the same total daily dose, administered orally every 6 hours for 24 hours.<br>- Day 3: Reduce the original opioid infusion by approximately 20% and continue methadone orally every 6 hours.<br>- Day 5: Reduce the original opioid infusion by approximately 20% and continue methadone orally every 8 hours.<br>- Day 7: Reduce the original opioid infusion by approximately 20% and continue methadone orally every 12 hours.<br>- Day 9: Reduce the original opioid infusion by approximately 20% and continue methadone orally every 24 hours.<br>- Day 11: Discontinue methadone<br>*The maximum daily dose of methadone is not to exceed 40 mg/day.*<br>- Conclusions by authors related to efficacy/safety<br>P patients were weaned significantly faster than NP patients (median, 9 days and 20 days, respectively). P patients requiring short-term opiate use also weaned significantly faster than short-term NP patients (median, 5 days and 21.5 days, respectively). Withdrawal complications were seen in three NP patients with weaning delayed in two. Two P patients had withdrawal complications with no delay in weaning. Significant methadone calculation discrepancy occurred in one NP patient but in no P patients. Use of a standardized methadone-weaning protocol was associated with decreased duration of methadone use compared with previous practice without any increase in withdrawal or other side effects.<br>- Other considerations<br>Caution should be exercised in extrapolating its use in neonates and infants < 5 kg. The study did not use the protocol in any patient < 3.5 months old or weighing 6 kg.	

DOI	- Prospective study<br>- N=107 (preintervention: N=68, postintervention N=39)<br>- Level of evidence: B	**Implementation of a risk-stratified opioid weaning protocol in a pediatric intensive care unit.**<br>Sanchez-Pinto LN, 2018 (16)<br><br>Summary of study results<br>- <u>Objective of study</u><br>To implement a risk-stratified opioid weaning protocol to reduce the duration of opioids without increasing the incidence of withdrawal.<br>- <u>Study design</u><br>A pre- and post-interventional prospective study was undertaken in a large children's hospital pediatric ICU where a risk-stratified opioid weaning protocol was implemented. Patients were included if exposed to ≥ 7days of scheduled opioids. The primary outcome was duration of opioids and secondary outcome was hospital LOS. Patients were stratified into withdrawal risk categories based on the length of exposure to opioids. Risk categories were defined as continuous exposure to opioids before weaning for: 1) less than five days (low risk), 2) five to seven days (moderate risk), 3) between seven and 30 days (high risk), and 4) N30 days (very high risk). A prescribed weaning strategy was defined for each risk category. The WAT-1 scoring system is a validated score with good inter-rater reliability that was in use and part of the standard of care in our PICU prior to this study. Patients were defined as having significant withdrawal if their WAT-1 score was equal or greater than four and two greater than the baseline WAT-1, consistent with published thresholds.<br>- <u>Opioid use</u><br>The institution primarily uses fentanyl up to 4 mcg/kg/h, then hydromorphone for continuous opioid infusions.<br>- <u>Rationale for methadone dose</u><br>Methadone dosing can be derived from the fentanyl infusion rate. For infusion rates between 1 and 4 µg/kg/h, the corresponding enteral methadone dose typically ranges from 0.05 to 0.15 mg/kg administered every 8 hours, with higher infusion rates requiring higher methadone doses.<br>- <u>Taper schedule</u><br>The opioid weaning protocol consisted of three main parts: (a) an algorithm to delineate a weaning schedule stratified by risk of withdrawal (low, moderate, high and very high risk), (b)a table to convert the opioid infusion to a weaning medication, and (c) a bedside worksheet used by clinicians to track withdrawal symptoms as measured by the withdrawal assessment tool-1 (WAT-1). High and very high risk patients were recommended to convert to withdrawal-prevention doses of oral methadone that were not equianalgesic (0.05 to 0.15 mg/kg every 8 h).	Dose recommendation suggested by this study: -<br><br>- Indication: prevention of opioid withdrawal symptoms in the PICU<br>- Age/weight: years (IQR)<br>▪ Preintervention: 2.1 (0.6-9.8)<br>▪ Postintervention: 2.3 (0.4-14.2)<br>- Dose: mean unknown<br>- RoA: oral<br>- Duration of use: days (IQR)<br>▪ Preintervention: 18 (11.5-26.5)<br>▪ Postintervention: 12 (10-19)<br><br>Critical appraisal of study results /considerations/remarks<br>- Fixed-dose methadone: only for high-risk and very-high risk patients (moderate-risk patients used IV hydromorphone)<br>- The study used the WAT-1 to evaluate opioid withdrawal symptoms

		The tapering schedule is a reformulated text-based version of the protocol described by Sanchez-Pinto et al. (2018).<br>Low risk (<5 days)<br>- Discontinue opioid therapy without a structured taper.<br>Moderate risk (5-7 days), wean duration 5 days<br>- Convert continuous opioid infusion to intermittent IV hydromorphone.<br>- Discontinue the opioid infusion at time of conversion.<br>- Start hydromorphone (e.g. 0.1 mg/kg IV every 8 hours).<br>- Gradually taper the total daily opioid dose (e.g. ~20% per day).<br>High risk (8-30 days), wean duration 10 days<br>- Initiate enteral methadone dose.<br>- Continue opioid infusion during initial methadone dosing.<br>- After second methadone dose, reduce opioid infusion by 50%; after third methadone dose, reduce opioid infusion further by 50%.<br>- Continue stepwise reduction of opioid infusion.<br>- Discontinue opioid infusion after sequential methadone dosing.<br>- Taper methadone: reduce every other day by 20%.<br>Very risk (>30 days), wean duration >10 days<br>- Initiate enteral methadone dose.<br>- Continue opioid infusion during initial methadone dosing.<br>- After third methadone dose, reduce opioid infusion by 50%; after sixth methadone dose, reduce opioid infusion further by 50%.<br>- Continue stepwise reduction of opioid infusion.<br>- Discontinue opioid infusion after sequential methadone dosing.<br>- Taper methadone: reduce every other day by 10%.<br>- Conclusions by authors related to efficacy/safety<br>Patients in the post-intervention group had significantly shorter median length of scheduled opioids (17 vs. 22.5 days) and opioid wean (12 vs. 18 days), and decreased total exposure to opioids (33.2 vs 48.8 mg/kg/patient) than those in the pre-intervention group. As hypothesized, the study found that patients in the post-intervention group had fewer days on opioids and decreased total cumulative exposure to opioids without an increase in withdrawal symptoms compared to patients in the pre-intervention group. In addition, there was an associated trend toward a decreased hospital length of stay, although this did not achieve statistical significance.	

<table>
<tr>
<td>DOI</td>
<td>- Retrospective review
- N=30
- Level of evidence: B</td>
<td>Methadone dosage for prevention of opioid withdrawal in children.
Siddappa R, 2003 (17)

Summary of study results
- <u>Objective of study</u>
To evaluate methadone dosing as a risk factor for opioid withdrawal and to determine optimal dose and efficacy of methadone to prevent withdrawal.
- <u>Study design</u>
Data from 30 children who received an opioid infusion for ≥ 7 days and subsequently received methadone for opioid withdrawal (between January 2000 and July 2001) were analysed. Nurses documented the presence or absence of withdrawal signs daily. The PICU physician evaluated the symptoms recorded to confirm opioid withdrawal. Patients were classified into two groups. No or minimal withdrawal (group 1) are patients with < 3 symptoms and did not receive additional methadone. Significant withdrawal (group 2) patients are those with ≥ 3 symptoms or received additional methadone dose to prevent or treat withdrawal.
- <u>Opioid use</u>
This table is an adapted and reformatted version of the data presented in Siddappa et al. (2003), for illustrative purposes only.
<table>
<tr><td></td><td>No withdrawal</td><td>Withdrawal</td></tr>
<tr><td>Fentanyl max. dose (mcg/kg/hr)</td><td>8.2 (4-117)</td><td>14 (7-55)</td></tr>
<tr><td>Fentanyl cumulative dose (mg/kg)</td><td>1.25 (0.5-5.9)</td><td>4.2 (1-10.5)</td></tr>
</table>
- <u>Rationale for methadone dose</u>
The methadone dose was based upon the dose of opioid the patient was receiving for sedation. Fentanyl is 100 times more potent than methadone and its duration of action is 30–60 min. Duration of action of methadone is 12–24 h (12). Considering duration of action of methadone as 24 h and duration of fentanyl 45 min (0.75 h) equipotent methadone dose is equal to 3 x total daily fentanyl dose. This methadone dose was initially given intravenously in three divided doses per day for 48 h. The fentanyl dose was tapered at 50% decrements starting from the second dose of methadone and the fentanyl infusion was stopped at the fourth methadone dose and the route of methadone administration was changed from intravenous to oral.
- Methadone has a high bioavailability so no dose correction is required for the i.v. to oral conversion.
Methadone dose (mg) = (fentanyl daily dose (mg) x fentanyl potency x duration of action of fentanyl)/duration of action of methadone = fentanyl dose x 100 x 0.75/24 = fentanyl daily dose x 3.</td>
<td>Dose recommendation suggested by this study: methadone dose of 2.5 times the daily fentanyl dose (mg) as the starting dose of methadone for opioid withdrawal protocols

- Indication: prevention of opioid withdrawal in children in the PICU
- Age/weight: 0.1 – 16.2 years
- Dose: mean unknown
- RoA: IV and oral
- Duration of use: unknown

Critical appraisal of study results /considerations/remarks
- Fentanyl IV : methadone IV/oral 1 : 3 (daily dose)
- IV and oral methadone were used in this population/protocol
- No scoring system was used in the study to evaluate opioid withdrawal symptoms</td>
</tr>
</table>

		- <u>Taper schedule</u><br>The methadone wean was started the day after oral conversion and the rate of weaning was between 3 and 10% per day depending on the dose and duration of opioid sedation. During the methadone wean if the patient exhibited withdrawal symptoms an additional 10–20% of the methadone dose was administered intravenously by the PICU team on an as needed basis until the symptoms were controlled and the maintenance dose was increased accordingly if necessary.<br>- <u>Conclusions by authors related to efficacy/safety</u><br>Twenty patients had no or minimal withdrawal symptoms and 10 experienced significant withdrawal. To determine the optimal dose of methadone that would prevent withdrawal a ROC analysis was performed. The ROC was 0.80. The dose of methadone that had the highest sensitivity (90%) and specificity (70%) for preventing withdrawal was 80% of that derived from the formula (= 2.4 times the fentanyl dose per day in mg). This was then used to calculate the odds ratio of developing withdrawal because of an inadequate dose of methadone. The odds ratio to develop withdrawal with < 80% recommended methadone dose is 21%.	

DOI	- Partly prospective, partly retrospective cohort study<br>- Pretest N=219; posttest N=233<br>- Level of evidence: B	Effect of a sedation weaning protocol on safety and medication use among hospitalized children post critical illness<br>Solodiuk JC, 2019 (18)<br><br>Summary of study results<br>- <u>Objective of study</u><br>To decrease patients with prescriptions for high-risk medications (opioids, benzodiazepines and clonidine)<br>- <u>Study design</u><br>An evidence-based post PICU sedation weaning guideline was created and implemented over time with ongoing education, and review of progress with staff members with pre-post evaluation.<br>- <u>Taper schedule</u><br>*The tapering schedule is a reformulated text-based version of the protocol described by Solodiuk et al. (2019).*<br>Low risk (<5 days)<br>- No wean. Stop opioid and monitor patient for signs and symptoms of withdrawal.<br>Normal risk (5-20 days)<br>- Wean morphine 10% q8 hours. Wean off in 3 days.<br>High risk (>20 days)<br>- Start initial morphine wean 10% q24 hours. Increase to 15-20% q24 hours if patient tolerates.<br>- <u>Conclusions by authors related to efficacy/safety</u><br>Post intervention, there were significant differences in the numbers of patients discharged on home weaning from both opioids and benzodiazepines (11%, n = 24/219 pre; 3%, n = 7/233 post; p b 0.005). The number of patients discharged with a methadone wean decreased (7%, n = 16/219 pre; 0%, 0/233 post; p = 0.03). Despite these differences, there were no significant differences in the median hospital length of stay (42 pre; 39 post; p = 0.35). Post implementation more children had mild to moderate symptoms of withdrawal (11% pre; 21% post; p b 0.005) as compared to pre-implementation, however, the percentage of severe symptoms remained consistently low (0.6% pre; 1% post; p = 0.11)	Dose recommendation suggested by this study:-<br><br>- **Indication**: tapering of opioids after pain treatment in PICU<br>- **Age/weight**: median (IQR)<br>Pretest: 0.2 years (0.01-1.4)<br>Posttest: 0.8 years (0.04-6.1)<br>- **Dose**: mean unknown<br>- **RoA**: unknown<br>- **Duration of use**: duration<br><br>Critical appraisal of study results /considerations/remarks:<br>- The study used the WAT-1 to evaluate opioid withdrawal symptoms

DOI	- Retrospective study<br>- N=42 (SCG: N=21, RCG: N=21)<br>- Level of evidence: B	Conversion from prolonged intravenous fentanyl infusion to enteral methadone in critically ill children. Srinivasan V, 2017 (19)<br><br>Summary of study results<br>- <u>Objective of study</u><br>To describe our institutional experience with conversion from intravenous (IV) fentanyl infusion directly to enteral methadone and occurrence of withdrawal in critically ill mechanically ventilated children exposed to prolonged sedation and analgesia.<br>- <u>Study design</u><br>Patients were included if they were between 0-18 years of age, invasively mechanically ventilated via endotracheal tube or tracheostomy, on IV fentanyl infusion for more than 5 d and started on scheduled enteral methadone with the intention to wean off the IV fentanyl infusion completely. All patients were monitored for opioid withdrawal symptoms using the Withdrawal Assessment Tool-Version 1 (WAT-1). Patients were classified into the rapid conversion group (RCG) if they were completely converted from IV fentanyl infusion to enteral methadone in 48 h or less, or the slow conversion group (SCG) if they were completely converted from IV fentanyl infusion to enteral methadone in more than 48 h. The primary outcome measure was difference in WAT-1 scores between the RCG and the SCG at 7 d from the time of enteral methadone initiation. Secondary outcome measures were differences in WAT-1 scores at 12, 24, 48, 72 and 96 h from the time of enteral methadone initiation, as well as overall WAT-1 and overall pain scores during the 7 d from enteral methadone initiation. Additional secondary outcomes included differences in ventilator free days at 28 d (VFD), PICU length of stay (LOS), and use of daily rescue opioids and concomitant medications.<br>- <u>Opioid use</u><br>Cumulative dose of IV fentanyl infusion at time of initiation of oral methadone (mg/kg) (median, IQR)<br>▪ SCG: 1.64 (1.03-1.98)<br>▪ RCG: 1.48 (1.11-1.92)<br>Duration of IV fentanyl infusion prior to initiation of oral methadone (days) (median, IQR)<br>▪ SCG: 10 (8-21)<br>▪ RCG: 9 (8-14)<br>- <u>Rationale for methadone dose and taper schedule</u><br>The initial dose conversion from IV fentanyl infusion to enteral methadone was determined by the clinical team based on clinical judgment and in discussion with the Clinical Pharmacist.<br>The suggested time frame for conversion from IV fentanyl infusion to enteral methadone was usually 48 h, but was not standardized and left to attending physician discretion. After the second dose of enteral methadone, the IV fentanyl infusion was decreased by 50%. After the third dose	Dose recommendation suggested by this study: -<br><br>- **Indication**: prevention of opioid withdrawal symptoms in the PICU<br>- **Age/weight**: median years (IQR)<br>▪ SCG: 2 (0.8-4.0)<br>▪ RCG: 1 (0.3-3.5)<br>- **Dose**: mean unknown<br>- **RoA**: oral<br>- **Duration of use:** 7 days<br><br>Critical appraisal of study results /considerations/remarks<br>- Fentanyl IV : methadone oral 1 : 2.5 (daily dose)<br>- The study used the WAT-1 to evaluate opioid withdrawal symptoms

		of enteral methadone, the IV fentanyl infusion was decreased by a further 50%. After the fourth dose of enteral methadone, the IV fentanyl infusion was typically discontinued. Thereafter, the dosing of enteral methadone was adjusted by the attending physician to prevent both withdrawal symptoms as well as oversedation.<br>Conversion from intravenous fentanyl to enteral methadone was performed using a stepwise equivalence-based approach.<br>For example, in a child weighing 10 kg receiving a fentanyl infusion of 5 µg/kg/h, the total daily fentanyl dose corresponds to approximately 1.2 mg.<br>This fentanyl dose was converted to methadone using a conversion factor of approximately 2.5, resulting in a total daily methadone dose of about 3 mg. This dose was divided into two administrations of 1.5 mg given every 12 hours.<br>During the transition phase, the fentanyl infusion was maintained initially. After the second methadone dose, the fentanyl infusion was reduced by approximately 50%. Following the third methadone dose, the infusion was reduced again by 50%, and after the fourth methadone dose, the fentanyl infusion was discontinued.<br>- Conclusions by authors related to efficacy/safety<br>Compared to SCG, RCG had lower median WAT-1 scores at 7 d (2.5 vs 5). Additionally, RCG had lower overall median pain scores (3 vs 6), and required less median daily rescue opioids (3 vs 12) than SCG. The starting daily median methadone dose was 2.3 times the daily median fentanyl dose in the RCG, compared to 1.1 times in the SCG. In those children who converted successfully from IV fentanyl infusion to enteral methadone within a period of 48 h, a methadone: fentanyl dose conversion ratio of approximately 2.5:1 appeared to minimize withdrawal with less need for rescue opioids.	

DOI	- Retrospective chart review<br>- N=52 (protocol group: N=20, control group: N=32)<br>- Level of evidence: B	Evaluation of a pharmacist-managed methadone taper.<br>Steineck KF, 2014 (20)<br><br>Summary of study results<br>- <u>Objective of study</u><br>To evaluate the efficacy of a pharmacist-managed methadone taper as compared to previous prescribing practices.<br>- <u>Study design</u><br>To assess the primary objective, the study measured length of methadone taper, number of additional doses of opioid used during the taper, number of days patients remained on opioid infusions in addition to methadone, withdrawal scores, and length of hospital stay. Patients were stratified into groups for risk of withdrawal based on their opioid exposure. Patients were considered low risk if they had received less than 5 days of an opioid infusion, moderate risk if they received 5–9 days, high risk if they received greater than or equal to 10 days or a cumulative fentanyl dose of 1.500–2.499 µg/kg or opioid equivalent, and they were considered very high risk if they had received greater than or equal to 28 days or a cumulative fentanyl dose of greater than or equal to 2.500 µg/kg or opioid equivalent. Withdrawal scores were calculated from a modified Finnegan scoring tool that was developed within the institution and was in place during the study period.<br>- <u>Taper schedule</u><br>The taper schedule was based on clinical experience managing withdrawal at the institution.<br>- <u>Conclusions by authors related to efficacy/safety</u><br>The average taper length before pharmacist intervention was 24.7 days; after implementation it was 15 days. There was no statistical difference in the number of additional doses of opioid required or withdrawal scores between the populations. Opioid infusions were stopped, on average, 1.54 days sooner in the intervention group. A decrease in hospital length of stay was also seen in the intervention group. Despite the shorter methadone taper, decreased length of time on continuous opioids, and fewer as-needed doses of morphine, there was no increase in withdrawal scores of the intervention group, and the average scores were in an acceptable range.	Dose recommendation suggested by this study:-<br><br>- Indication: prevention of opioid withdrawal symptoms in the PICU<br>- Age/weight: (months)<br>▪ Protocol group: 27.6 (1-180)<br>▪ Control group: 18.7 (1-192)<br>- Dose: mean unknown<br>- RoA: IV or oral<br>- Duration of use: mean (range)<br>▪ Protocol group: 24.7 days (7-72)<br>▪ Control group: 15 days (3-34)<br><br>Critical appraisal of study results /considerations/remarks<br>- Fixed-dose methadone based on total cumulative fentanyl dose (mg/kg) or duration of use<br>- The study used a modified Finnegan scoring tool to evaluate opioid withdrawal symptoms

DOI	- Open-label, randomized controlled trial<br>- N=30<br>- Level of evidence: B	**Protocolized Sedative Weaning vs Usual Care in Pediatric Critically Ill Patients: A Pilot Randomized Controlled Trial**<br>**Tiacharoen D 2020 (21)**<br><br>**Summary of study results**<br>- Objective of study<br>To establish a sedative-weaning protocol and compare withdrawal symptoms between protocolized weaning and usual care in critically ill children at risk for withdrawal syndrome.<br>- Study design<br>Patients aged 1 month to 18 years who received continuous opioid and/or benzodiazepine infusions for at least 5 days were enrolled in an open-label randomized controlled trial. Patients were randomized to either a protocolized weaning group (n = 19) or usual care group (n = 11). Patients with pre-existing withdrawal syndrome, methadone allergy, significant drug interactions with methadone, or end-stage disease were excluded.<br>- Opioid use<br>Median cumulative fentanyl dose before enrollment:<br>• Protocol group: 521.9 µg/kg<br>• Usual care group: 470.0 µg/kg<br>Median duration of fentanyl exposure: approximately 9–10 days.<br>- Rationale for methadone dose<br>A standardized protocol was developed based on previous studies. Fentanyl infusions were converted to oral methadone according to the pre-conversion fentanyl dose and patient weight. Fentanyl IV : Methadone PO = 1 : 6.5.<br>- Taper schedule<br>(Reduced doses 50% in patients who had renal or hepatic impairment)<br>**High-risk patients (when a cumulative dose > 0.5 mg/kg of fentanyl equivalent doses or duration of sedative administration > 10 days)**<br>• Methadone reduced by 10% of pre-taper dose daily.<br>**Low-risk patients**<br>• Methadone reduced by 20% of pre-taper dose daily.<br>**Multiple medications**<br>• If receiving more than one sedative, medications were tapered on alternating days.<br>- Conclusions by authors related to efficacy/safety<br>The implementation of a protocolized sedative-weaning protocol did not reduce the overall incidence of iatrogenic withdrawal syndrome compared with usual care (84% vs 81%). However, the protocol significantly reduced the percentage of withdrawal days, the need for rescue	**Dose recommendation suggested by this study: -**<br><br>- **Indication**: prevention of iatrogenic withdrawal syndrome (IWS)<br>- **Age/weight**: protocol group: 1.57 (IQR 0.81-4.91); usual care; usual care: 1.73 (IQR 0.53-5.44)<br>- **Dose**: protocol group: mean 0.17 mg/kg/day (IQR 0.13-0.23); usual care: mean 0.22 (IQR 0.17-0.35)<br>- **RoA**: oral<br>- **Duration of use**: median methadone duration: protocol group: 10 days usual care group: 10 days<br><br>**Critical appraisal of study results /considerations/remarks**<br>- Fentanyl IV : methadone oral 1 : 6.5 (= 1 : 26 daily dose)<br>- Morphine IV : methadone oral 3.85 : 1 (daily dose)<br>- The study used the WAT-1 to evaluate opioid withdrawal symptoms

		morphine therapy, and the length of ICU stay. No increase in adverse drug reactions was observed. The authors concluded that the protocol improved clinically relevant outcomes and could be safely implemented, although external validation is required.	
DOI	- Case reports<br>- N=3<br>- Level of evidence: C	Methadone as treatment for iatrogenic narcotic dependency in pediatric intensive care unit patients<br>Tobias JD, 1990 (22)<br><br>Summary of study results<br>Patient 1:<br>- Age: neonate. After 1 month: 0.1 mg/kg methadone every 12 hr by NG tube.<br>- Opioid: fentanyl 1 – 2 mcg/kg every 1 to 2 hr during neonatal period.<br>- Dose methadone: 0.1 mg/kg every 12 hr. Weaned by 10% to 20% every week and discontinued after 6 weeks. After 6 weeks no further withdrawal symptoms were noted.<br><br>Patient 2:<br>- Age: neonate (3 weeks). After 8 weeks: 0.1 mg/kg every 12 hr methadone by NG tube.<br>- Opioid: fentanyl 2 – 3 mcg/kg/hr during neonatal period (3 weeks). After 4 days fentanyl was discontinued and morphine 0.1 – 0.15 mg/kg/dose was started for the next 8 weeks.<br>- Methadone: 0.1 mg/kg every 12 hr. Methadone was decreased by 0.01 mg/kg/wk for the next 6 weeks, then discontinued with no recurrence of symptoms.<br><br>Patient 3:<br>- Age: neonate. After 4 weeks: 0.1 mg/kg every 12 hr methadone by NG tube.<br>- Opioid: fentanyl 1 – 2 mcg/kg every 1 to 2 hr during neonatal period followed by morphine 0.1 mg/kg every 3 hr.<br>- Methadone: 0.1 mg/kg every 12 hr. Methadone was weaned slowly over the next 4 weeks.<br><br>Overall dose: 0.1 mg/kg by every 12 hr by NG tube, then decreased by 10 to 20% every week. No recurrence of symptoms was noted. No additional narcotics were necessary.	Dose recommendation suggested by this study:-<br><br>- Indication: narcotic addiction in infants who underwent surgery for congenital heart disease and required prolonged postoperative ventilation and intensive care management<br>- Age/weight: see study results<br>- Dose: 0.1 mg/kg every 12 hr<br>- RoA: NG tube<br>- Duration of use: 4-6 weeks<br><br>Critical appraisal of study results /considerations/remarks<br>- Fixed-dose methadone

DOI	- Case report<br>- N=1<br>- Level of evidence: C	**Outpatient therapy of iatrogenic drug dependency following prolonged sedation in the pediatric intensive care unit**<br>**Tobias JD, 1994 (23)**<br><br>**Summary of study results**<br>- Opioid: morphine PCA 2 mg/hr with 2 mg every 10 min as needed. Morphine changed to fentanyl 20 mcg/hr and 20 mcg every 5 min as needed. Fentanyl increased to 40 mcg/hr with 40 mcg every 5 min as needed.<br>- Methadone: 2 mg every 12 hr (0.05 mg/kg). Within 24 h, the symptoms resolved.<br>- Taper schedule: 2 mg 2 bid week 1, 1 mg 2 bid week 2, 0.5 2 bid week 3, and 0.5 1 bid week 4. The patient completed the taper schedule without problems or signs of opioid withdrawal.<br><br>In the discussion section of the study:<br>Conversion from intravenous fentanyl to oral methadone requires consideration of pharmacological differences, including potency, half-life, and oral bioavailability.<br>For example, in a 10-kg patient receiving a fentanyl infusion of 10 µg/kg/h, the total daily fentanyl dose corresponds to approximately 2.4 mg.<br>An equivalent methadone dose can be estimated by accounting for differences in potency (approximately 100:1 fentanyl to methadone), pharmacokinetics, and oral bioavailability (approximately 75%). Based on these factors, the calculated total daily oral methadone dose is approximately 7.2 mg.<br>Methadone is then administered enterally, typically divided into twice-daily dosing.<br>During the transition phase, the intravenous opioid infusion is gradually tapered over 12–24 hours. Additional intravenous opioid doses may be provided as needed to manage withdrawal symptoms.<br>Patients should be monitored closely during the initial period, typically for 3–4 days, after which methadone is gradually reduced by approximately 30–50% per week until discontinuation.	**Dose recommendation suggested by this study:-**<br><br>- **Indication**: prevention or treatment of withdrawal symptoms in the PICU patient<br>- **Age/weight**: 11 years, 36 kg<br>- **Dose**: 2 mg every 12 hr (0.05 mg/kg)<br>- **RoA**: oral<br>- **Duration of use**: 5 weeks<br><br>**Critical appraisal of study results /considerations/remarks**<br>- In Table 1, the study calculated a daily methadone dose of 7.2 mg. When calculating backwards, they used a bioavailability of 67% for methadone, as the numbers would not align otherwise. The study mentions a bioavailability range of 50–75% for methadone<br>*2.4 mg/day fentanyl x 100 = 240 mg/day methadone / 50 = 4.8 mg/day methadone / 0.75 = 6.4 mg/day methadone. 6.4 / 2.4 = 2.67*<br>*≈ 1 : 3*

DOI	- Retrospective chart review<br>- N=18<br>- Level of evidence: B	Outpatient therapy of iatrogenic drug dependency following prolonged sedation in the pediatric intensive care unit.<br>Tobias JD, 1996 (24)<br><br>Summary of study results<br>- <u>Objective of study</u><br>To present the additional experience in 18 patients with use of methadone for out-patient therapy in this patient population.<br>- <u>Study design</u><br>Symptoms of opioid withdrawal (as assessed by the Finnegan score) or excessive sedation that occurred after switching to methadone were noted. Problems with out-patient follow-up and methadone administration were determined.<br>- <u>Opioid use</u><br>The duration of the fentanyl infusion varied from 12 to 55 days, whereas the maximum rate of fentanyl infusion was 9 to 24 mcg/kg/hr in 17 of 18 patient.<br>- <u>Rationale for methadone dose</u><br>Generally, the dose is not increased to compensate for the oral bioavailability or the two-fold difference based on potency and half-life. Clinical experience suggests this compensation is not needed to prevent withdrawal symptom. It may be that the higher dose is needed to provide sedation, but not to prevent withdrawal. Therefore, the methadone starting dose is equivalent to the total daily fentanyl dose (i.e., methadone, 2.4 mg/day). Rescue doses of morphine are 0.05 mg/kg. The total morphine requirement over a 24-hour period is added to the next day's methadone dose. Additional rescue doses of morphine are used as needed during the next 72-hour period, but no change is made in the subsequent methadone dose until 72 hours have elapsed to allow for attainment of a steady-state serum concentration of methadone. If excessive sedation occurs, one methadone dose is withheld, and the dose is decreased by 10 to 20%.<br>- <u>Taper schedule</u><br>Oral methadone is tapered once a week. The dose is decreased by 20% of the initial dose so that the oral dose is gradually decreased and then discontinued after a 5- to 6-week period.<br>- <u>Conclusions by authors related to efficacy/safety</u><br>All patients were successfully converted to oral methadone using the guidelines of the described protocol. Two patients were judged to be excessively sedated (i.e. required tactile stimuli to rouse). The next dose of methadone was withheld, and subsequent doses were decreased by 10 to 20%. During the switch from intravenous fentanyl to oral methadone, all patients were monitored with continuous pulse oximetry. Room air oxygen saturations of less than 93% developed in none. Three patients manifested symptoms of mild opioid withdrawal (according to the Finnegan score) during the initial 72-hour period after changing over to oral methadone.	Dose recommendation suggested by this study:-<br><br>- **Indication**: prevention of opioid withdrawal in the PICU<br>- **Age/weight**: 3 months – 16 years, 4.8 – 51 kg<br>- **Dose**: mean unknown<br>- **RoA**: oral<br>- **Duration of use**: 5-6 weeks<br><br>Critical appraisal of study results /considerations/remarks<br>- No correction on potency, $t_{1/2}$ or F<br>- Fentanyl IV : methadone oral 1 : 1 (daily dose)<br>- The study used the Finnegan Score to evaluate opioid withdrawal symptoms

		These three patients received "rescue doses" of intravenous morphine, and the subsequent dose of methadone was modified. No other symptoms of withdrawal were noted.<br>- Other considerations<br>Not other changes should be made for at least 72 hours following a dose adjustment of methadone because it may take this long to achieve a new steady-state serum concentration as a result of the new dose.	

DOI	- Prospective cohort study<br>-N=46 (standardized protocol group: N=22, retrospective control group: N=24)<br>- Level of evidence: B	Shortened Taper Duration after Implementation of a Standardized Protocol for Iatrogenic Benzodiazepine and Opioid Withdrawal in Pediatric Patients: Results of a Cohort Study.<br>Vipond JM, 2018 (25)<br><br>Summary of study results<br>- <u>Objective of study</u><br>A standardized pharmacist-managed methadone and lorazepam taper protocol was implemented at a pediatric tertiary care facility with the aim to reduce the length of taper for patients with iatrogenic withdrawal.<br>- <u>Study design</u><br>The primary endpoint was the length of methadone and/or lorazepam taper. Secondary endpoints included evaluation for significant differences between the control and standardized protocol groups regarding additional breakthrough withdrawal medications, pediatric intensive care unit (PICU) and hospital length of stay. The critical care nurses assessed patients for withdrawal symptoms every 12 hours using the Withdrawal Assessment Tool-1 (WAT-1).<br>- <u>Opioid use</u><br>Cumulative morphine, median dose (mg/kg):<br>▪ Retrospective control group: 19.7 ± 16.5<br>▪ Standardized protocol group: 21.6 ± 16.4<br>- <u>Taper schedule</u><br>The study protocol, modified from Steineck et al. (2014), was created by a group of pharmacists and critical care physicians after a literature review and consultation with additional pediatric institutions regarding dosing and scheduling for the taper.<br>*The tapering schedule is a reformulated text-based version of the protocol described by Vipond et al. (2018).*<br>No treatment required for patients with <5 days exposure unless withdrawal occurs.<br>Low-risk patients (<5 days opioid/BZD infusion)<br>Methadone:<br>- Day 1: 0.05 mg/kg per dose PO every 8 hours (3 doses)<br>- Day 2: 0.05 mg/kg per dose PO every 12 hours (2 doses)<br>- Day 3: 0.05 mg/kg per dose PO every 24 hours (1 dose)<br>Moderate-risk patients (5-9 days opioid/BZD infusion)<br>- Day 1: 0.1 mg/kg per dose PO every 8 hours (3 doses)<br>- Day 2: 0.08 mg/kg per dose PO every 8 hours (3 doses)<br>- Day 3: 0.06 mg/kg per dose PO every 8 hours (3 doses)<br>- Day 4: 0.04 mg/kg per dose PO every 8 hours (3 doses)	Dose recommendation suggested by this study:-<br><br>- Indication: prevention of opioid withdrawal symptoms in the PICU<br>- Age/weight: (months)<br>▪ Standardized protocol group: 26.6 ± 42.2<br>▪ Retrospective control group: 7.0 ± 10.5<br>- Dose: mean unknown<br>- RoA: oral<br>- Duration of use:<br>▪ Standardized protocol group: 6.0 days (IQR: 3.0-9.0)<br>▪ Retrospective control group: 9.5 days (IQR: 5.5–14.5)<br><br>Critical appraisal of study results /considerations/remarks<br>- Fixed-dose methadone based on total cumulative morphine dose (mg/kg) or duration of use<br>- The study used the WAT-1 to evaluate opioid withdrawal symptoms<br>- Almost all previously published studies on withdrawal using methadone were included in the review for developing the protocol used in this study<br>- The standardized protocol cohort had older children compared with the control, which may have

		- Day 5: 0.04 mg/kg per dose PO every 12 hours (2 doses)<br>- Day 6: 0.04 mg/kg per dose PO every 24 hours (1 dose)<br>**High-risk patients (>10 days opioid/BZD infusion)**<br>Methadone:<br>- Day 1: 0.1 mg/kg per dose PO every 6 hours (8 doses)<br>- Day 3: 0.1 mg/kg per dose PO every 8 hours (6 doses)<br>- Day 5: 0.08 mg/kg per dose PO every 8 hours (6 doses)<br>- Day 7: 0.06 mg/kg per dose PO every 8 hours (6 doses)<br>- Day 9: 0.04 mg/kg per dose PO every 8 hours (6 doses)<br>- Day 11: 0.04 mg/kg per dose PO every 12 hours (4 doses)<br>- Day 13: 0.04 mg/kg per dose PO every 24 hours (2 doses)<br>**Very high-risk patients (>28 days opioid/BZD infusion)**<br>Methadone:<br>- Day 1: 0.2 mg/kg per dose PO every 6 hours (8 doses)<br>- Day 3: 0.2 mg/kg per dose PO every 8 hours (6 doses)<br>- Day 5: 0.17 mg/kg per dose PO every 8 hours (6 doses)<br>- Day 7: 0.14 mg/kg per dose PO every 8 hours (6 doses)<br>- Day 9: 0.11 mg/kg per dose PO every 8 hours (6 doses)<br>- Day 11: 0.08 mg/kg per dose PO every 8 hours (6 doses)<br>- Day 13: 0.05 mg/kg per dose PO every 8 hours (6 doses)<br>- Day 15: 0.05 mg/kg per dose PO every 12 hours (4 doses)<br>- Day 17: 0.05 mg/kg per dose PO every 24 hours (2 doses)<br>- Conclusions by authors related to efficacy/safety<br>In the methadone group, 22.7% of patients (5/22) had a deviation from the standard protocol. One patient required an increase in methadone dose, 2 patients were initiated on a lower starting dose per physician discretion, 1 patient's taper was shortened to facilitate discharge, and 1 patient had the dose of methadone decreased due to concerns for excess sedation. Median methadone taper length before protocol implementation was 9.5 days with an interquartile range (IQR) of 5.5–14.5 days; after protocol implementation, it was 6.0 (IQR, 3.0–9.0) days (P = 0.0145). The use of a standardized withdrawal protocol resulted in shorter taper duration for both the methadone and lorazepam groups. There was no difference in PICU or hospital length of stay.<br>- Other considerations<br>There was a trend toward increased utilization of intravenous morphine and/or lorazepam for breakthrough withdrawal symptoms in the standardized protocol group compared with the control group although the difference was not statistically significant. Although an increase in	

		breakthrough medication use might be correlated with increased withdrawal symptoms, the higher utilization was possibly secondary to several factors including availability of these medications, introduction of the WAT-1 scoring tool, and heightened awareness of withdrawal symptoms by nursing and providers. With the introduction of the WAT-1 scoring tool, nursing staff became more aware of withdrawal signs and symptoms, enabling them to readily recognize withdrawal and therefore quickly manage it appropriately with breakthrough medications.	

DOI	- Retrospective cohort study<br>- N=46 (preintervention: N= 25, postintervention: N= 26)<br>- Level of confidence: B	Iatrogenic Opiate Withdrawal in Pediatric Patients: Implementation of a Standardized Methadone Weaning Protocol and Withdrawal Assessment Tool.<br>Walters RA, 2021 (26)<br><br>Summary of study results<br>- Objective of study<br>To compare the duration of methadone exposure and the incidence of withdrawal symptoms pre- and post protocol implementation.<br>- Study design<br>The preintervention phase included patients weaned off of opiate infusions before implementation of a standardized weaning protocol and withdrawal symptom scoring tool. Patients in the postintervention phase were started on a standardized methadone wean based on total duration and dose of continuous opiate infusion exposure in the 24 hours preceding methadone initiation. Patients in the postintervention phase were monitored for withdrawal using the withdrawal assessment tool (WAT-1). Scores greater than or equal to 3 indicated clinically significant withdrawal. Intravenous morphine 0.05 mg/kg was available to manage symptoms of withdrawal.<br>- Rationale for methadone dose<br>Initial methadone dosing was based on the opioid infusion rate during the preceding 24 hours. For patients receiving morphine infusion at doses between 0.02 and 0.06 mg/kg/h, the initial methadone dose ranged from approximately 0.05 mg/kg administered every 6 hours to 0.1 mg/kg administered every 24–48 hours.<br>For morphine infusion rates between 0.07 and 1 mg/kg/h, methadone was typically initiated at 0.1 mg/kg per dose given every 6 hours for 24–48 hours.<br>In patients receiving fentanyl infusion, the methadone dose was adjusted according to infusion rate. A fentanyl infusion of 1 µg/kg/h corresponded to a methadone dose of approximately 0.1 mg/kg every 6 hours for 24–48 hours, whereas an infusion rate of 2 µg/kg/h corresponded to approximately 0.2 mg/kg every 6 hours over the same period.<br>Dose calculations were based on equivalency conversions, assuming an approximate 1:1 relationship between hourly morphine dose and methadone dose, and a fentanyl-to-methadone conversion factor of approximately 100:1. These adjustments also accounted for differences in pharmacokinetics and bioavailability.<br>- Taper schedule<br>The protocol was based on previous weaning strategies reported in the literature by Robertson et al. (2000). Morphine infusions were converted and rounded to either a 0.05 mg/kg or 0.1 mg/kg	Dose recommendation suggested by this study: -<br><br>- **Indication**: prevention of opioid withdrawal symptoms in the PICU<br>- **Age/weight**: months (median)<br>▪ Preintervention: 5.3 (1.3-24)<br>▪ Postintervention: 5.8 (3.5-14)<br>- **Dose**: mean unknown<br>- **RoA**: IV or oral<br>- **Duration of use**: (days)<br>▪ Preintervention: 17 (13-22)<br>▪ Postintervention: 5 (5-10)<br><br>Critical appraisal of study results /considerations/remarks<br>- The protocol is based on Robertson et al. (2000), and this study also included older children<br>- Morphine IV : methadone IV 6 : 1 (daily dose)<br>- Fentanyl IV : methadone IV 1 : 16.7 (daily dose)<br>After day 1, IV methadone was converted to oral methadone<br>- The study used the WAT-1 to evaluate opioid withdrawal symptoms<br>- In the preintervention phase, withdrawal assessment was dependent on the nurses' interpretation. his negated the

		dose of methadone in order to simplify order entry in the electronic health record (EHR). This daily dose was administered orally or intravenously based on the patient's status every 6 hours for the first 24 to 48 hours of the methadone taper, for patients receiving a 5-day and 10-day wean, respectively. The continuous opiate infusion the patient was receiving was reduced by 50% after the third dose of methadone and then discontinued after the fourth dose. Patients who had been exposed to continuous opiate infusions for 5 to 14 days received a 5-day wean, and patients exposed greater than 14 days received a 10-day wean.<br>- Conclusions by authors related to efficacy/safety<br>Total methadone duration decreased significantly from a median of 17 (13-22 interquartile range [IQR]) to 5 (5-10 IQR) days after implementation of the methadone weaning protocol. Number of morphine boluses administered increased from a median of 3 (0- 6 IQR) to 4 (0-5 IQR) doses per patient. This could be attributed to the increased peak opiate infusion dose and extended duration relative to the preintervention phase. Also, heightened awareness to withdrawal symptoms after receiving education on the WAT-1 tool and an "as-needed" order for morphine were only consistently present in the postintervention phase. Patients in the postintervention phase had significant reductions in methadone exposure after implementation of a standardized methadone weaning protocol and assessment tool.<br>- Other considerations<br>The majority of our patients were 2 years of age or younger, making it difficult to generalize this protocol to older children and adolescent.	opportunity for direct comparison of withdrawal incidence between the groups using a validated tool

DOI

- Retrospective study
- N=177 (pre protocol: N=75, post protocol N=102)
- Level of evidence: B

Exposure-Based Methadone and Lorazepam Weaning Protocol Reduces Wean Length in Children. Wilson AK, 2021 (27)

Summary of study results

- <u>Objective of study</u>
  Determine if a standardized methadone and lorazepam weaning protocol that is based on dose and duration of exposure can reduce the length of opioid and benzodiazepine weaning and shorten hospital stay.
- <u>Study design</u>
  The 3-day minimum was selected from our clinical experience with withdrawal symptoms in patients who received sedative/analgesic infusions for greater but not less than 3 days and is supported by research indicating a 20% risk of withdrawal in patients on opioid infusions for 3 to 5 days. Before protocol implementation, patients were weaned from opioids and benzodiazepines per attending physician discretion. Withdrawal symptoms were assessed clinically by the bedside nurse without a formalized withdrawal tool and communicated to the physician. Rescue doses of medication and adjustments of the wean in terms of dose, frequency, and weaning interval were made per the attending physician preference.
- <u>Opioid use</u>
  This table is an adapted and reformatted version of the data presented in Siddappa et al. (2003), for illustrative purposes only.

Outcome	Weaning category, N=	Pre-protocol, median (IQR)	Post-protocol, median (IQR)	*p* value
Duration of opioid infusion, days	No weaning<br>*Pre N= 20; Post N=24*	3.8 (3.5–4.3)	4.5 (3.8–4.7)	0.025
	Short-term weaning<br>*Pre N=45; Post N=68*	7.8 (6.5–10.3)	7.6 (5.9–9.5)	0.348
	Long-term weaning<br>*Pre N=9; Post N=9*	21.0 (16.8–33.5)	19.4 (16.1–24.7)	0.453
Cumulative opioid exposure, mg/kg	No weaning<br>*Pre N= 20; Post N=24*	18.0 (11.1–28.7)	18.7 (12.0–26.5)	1.00
	Short-term weaning<br>*Pre N=45; Post N=68*	52.1 (37.5–68.1)	36.2 (24.8–51.9)	0.002

Dose recommendation suggested by this study: -

- Indication: prevention of opioid withdrawal symptoms in the PICU
- Age/weight: median years (range)
  - Pre protocol: 0.6 (0-21.6)
  - Post protocol: 0.4 (0-20.5)
- Dose: mean unknown
- RoA: oral
- Duration of use: see conclusions

Critical appraisal of study results /considerations/remarks

- Fentanyl IV : methadone oral 1 : 8.3 (daily dose)
- Morphine IV : methadone oral 6 : 1 (daily dose)
- The study used the WAT-1 to evaluate opioid withdrawal symptoms

	Long-term weaning *Pre N=9; Post N=9*	N=9 166.2 (133.5–440.9)	172.1 (93.4–236.5)	0.436

- <u>Rationale for methadone dose</u>
  To convert opioid infusion dosing to methadone dosing, 50% of the equipotent dose was used to account for methadone's long half-life and high bioavailability. Specifically, the hourly rate of fentanyl (mcg/kg/hr) was multiplied by 0.05 to determine the starting dose of methadone (mg/kg), which was dosed every 6 hours. For morphine infusions, the hourly rate (mg/kg/hr) was multiplied by 1 to determine the starting dose of methadone (mg/kg), which was dosed every 6 hours. Both agents were converted to a maximum dose of 0.2 mg/kg/dose and never more than 10 mg/dose.
- <u>Taper schedule</u>
  The first enteral dose of methadone was given 24–48 hours before stopping the infusion. Subsequently, the infusion rate was reduced by 25% of the initial rate every 6 hours, beginning after the second enteral dose.

  <u>Short-course weaning (infusion duration 5–13 days)</u>
  1. Initial enteral dose administered every 6 hours for four doses.
  2. Dose reduced by 20% and continued every 6 hours for four doses.
  3. Administration interval extended to every 8 hours for three doses.
  4. Administration interval extended to every 12 hours for two doses.
  5. Methadone reduced to every 24 hours for one dose; lorazepam proceeded directly to the final taper step.
  6. If the methadone dose exceeded 0.1 mg/kg/dose, the dose was further reduced by 20% increments until these thresholds were reached, after which treatment was discontinued.

  <u>Long-course weaning (infusion duration >13 days)</u>
  1. Initial enteral dose administered every 6 hours for eight doses.
  2. Dose reduced by 20% and continued every 6 hours for eight doses.
  3. Administration interval extended to every 8 hours for six doses.
  4. Administration interval extended to every 12 hours for four doses.
  5. Methadone reduced to every 24 hours for two doses; lorazepam proceeded directly to the final taper step.
  6. If the methadone dose exceeded 0.1 mg/kg/dose, the dose was reduced by 20% every other day until the discontinuation threshold was reached.
- <u>Conclusions by authors related to efficacy/safety</u>
  Median number of days on methadone for patients on opioid infusions for 5 to 13 days was reduced from 8.5 to 5.7 days (n = 45 [pre], n = 68 [post]) and for patients on opioid infusions for

		≥14 days, from 29.7 to 11.5 days (n = 9 [pre], n = 9 [post]) after protocol implementation. There was no difference in complications between the prepotocol and postprotocol groups in terms of oversedation requiring the use of naloxone (none in both groups), re-admittance to the hospital for withdrawal (2.7% [n = 2] vs 1% [n = 1], or use of rescue doses of morphine median (IQR) 1 (0.3) vs 1 (0.4) or lorazepam 0 (0.2) vs 0 (0.2) for treatment of withdrawal.	

Qualifying evidence for studies

A1	Systematic reviews or meta-analyses involving at least some A2-level studies
A2	Good quality randomized comparative clinical trial of sufficient size and consistency according to Jadad classification (total score of 4 or 5)
B	Randomized clinical trials of poor quality or insufficient size (Corresponds to Jadad classification of 0-3) or other comparative study (non-randomized, comparative cohort study; patient-control study; retrospective study with sufficient patients and good controls).
C	Non comparative studies, case-reports (rechallenge), case series
D	Consensus, expert opinion

2. PBPK modeling

2.1 Methods

The PBPK platform Simcyp™ (version 23, Certara, Sheffield, UK) was used for all simulations. The PBPK models for methadone, fentanyl, and morphine were extracted from literature, using a standardized PubMed search query (see 2. Literature search queries). For methadone, the PBPK model of McPhail et al. was used, consisting of two compounds: the R- and S-enantiomer (28). The model was initially developed to predict the pharmacokinetic (PK) profile of neonates treated for neonatal abstinence syndrome (NAS).

Fentanyl and morphine models from Yao et al. and Emoto et al. were used, respectively, both exploring drug PK in the pediatric population (29-31). Default Simcyp™ populations were used; for adults we used the 'Sim-healthy volunteer' population or the 'Sim-NEurCaucasian' in case of subjects above the age of 65 years. For children, the 'Sim-Paediatric' population was used and for cytochrome P450 (CYP) 3A4, ontogeny profile 2 (by Upreti) was selected. The model was not verified in preterm neonates.

2.1.1 Model verification

To verify the PBPK models, we used the workflow as described by van der Heijden et al. (32), starting with the adult population to ensure that we had replicated the models correctly. McPhail et al. developed the PBPK models of R- and S-methadone for simulations in term neonates, so we subsequently verified the model in children. However, to assure that our replicated model was able to capture neonatal PK as well, we requested the raw PK data from the study by Wiles et al. (33) to check this. For the pediatric part, we used PK data from clinical PK studies to verify the models, extracted from the PubMed database using a standardized search query (see 2. Literature search queries).

The PBPK model for fentanyl was initially developed for the establishment of a virtual Chinese pediatric population, where fentanyl was one of four probe drugs used to evaluate the population (31). The morphine model was built to characterize its clearance in children and adults by including the organic cation transporter (OCT1) and the uridine 5'-diphosphate glucuronosyltransferase (UGT)2B7 (30). Both models were verified according to the similar workflow as described for methadone.

All models were verified with clinical PK studies by mimicking the virtual trial design (e.g., age range, proportion of females, administered dose, and trial duration) of each PK study. Ten trials with ten virtual subjects each were conducted. The PBPK compound models were used in their published form, without any changes (28, 30, 31). To assess the accuracy of the methadone model predictions (i.e., model performance), we calculated the predicted-to-observed PK parameter ratios for several PK parameters, for which a ratio within the 2-fold range was considered acceptable. In addition, we compared predicted plasma concentration-time profiles with observed data (i.e., visual predictive check).

2.1.2 Model simulations

Since organ maturation and enzyme ontogeny affect drug PK during childhood, we compared simulated methadone exposure, expressed as area under the curve (AUC), in various different age groups: 0-2 weeks postnatal age (PNA), 2-4 weeks PNA, 4 weeks-3 months, 3-6 months, 6-12 months, 1-2 years, 2-6 years, 6-12 years, and 12-18 years. For this comparison we used a 24-hour AUC at steady state. Methadone exposure of age groups that appeared similar were merged and the merged age groups were used for further simulations. The age group with the lowest concentrations was used for simulations comparing different conversion ratios and tapering schedules to ensure that all age groups would reach the minimum effective methadone concentration of 60 ng/mL.

To visualize the conversion from fentanyl or morphine to methadone, we simulated a 1 µg/kg/h infusion for fentanyl and a 10 µg/kg/h infusion for morphine. Ten trials of ten virtual subjects with 50% females were conducted in the selected age group. First, the fentanyl and morphine doses were converted to methadone according to several conversion ratios found in literature and local protocols (Table E) to define the most optimal conversion ratio resulting in methadone levels nearest by 60 ng/mL (7, 27, 34). We then simulated various tapering protocols based on the aforementioned fentanyl and morphine infusions, converted according to the optimal conversion ratios, to determine the most optimal tapering protocol. Lastly, the effect of age was explored by simulating the opioid conversion and methadone tapering in previously defined age groups.

Table E. Conversion simulations from fentanyl and morphine to methadone.

#	Drug	Dose	Conversion factor Drug : Methadone	Methadone dose
1	Fentanyl	1 µg/kg/h	1 : 3 (17, 23)	0.072 mg/kg/day
2		1 µg/kg/h	1 : 8.33 (27)	0.2 mg/kg/day
3		1 µg/kg/h	1 : 10 (34)	0.24 mg/kg/day
4		1 µg/kg/h	1 : 17.33 (7)	0.416 mg/kg/day
5	Morphine	10 µg/kg/h	6 : 1 (7, 27)	0.04 mg/kg/day
6		10 µg/kg/h	1 : 1 (34)	0.24 mg/kg/day

2.2 Literature search queries

A standardized PubMed search query was designed to find publicly available PBPK models. For 'drug' we used methadone, fentanyl, and morphine, respectively:

*(((("**drug**"[Title/Abstract] AND ((("physiolog*"[Title] OR "mechanistic*"[Title]) AND ("kinetic*"[Title] OR "pharmacokinetic*"[Title]) AND ("model*"[Title] OR "simulat*"[Title])) OR "pbpk*"[Title/Abstract] OR "simcyp*"[Title/Abstract] OR "gastroplus*"[Title/Abstract] OR "PK-Sim"[Title/Abstract] OR "physiologically based pharmacokinetic model*"[Title/Abstract] OR "physiology based pharmacokinetic model*"[Title/Abstract])) NOT ("pesticides"[MeSH Terms] OR "pesticide*"[Title/Abstract] OR "insecticide*"[Title/Abstract] OR "herbicide*"[Title/Abstract] OR "fungicide*"[Title/Abstract] OR "rodenticide*"[Title/Abstract] OR "environmental pollution"[MeSH Terms] OR "metal*"[Title/Abstract] OR "environmental*"[Title/Abstract] OR "toxicant*"[Title/Abstract])) NOT (("animals"[MeSH Terms] NOT "humans"[MeSH Terms]) OR (("Mouse"[Title] OR "Mice"[Title] OR "Rat"[Title] OR "Rats"[Title]) NOT ("Man"[Title] OR "Men"[Title] OR "Woman"[Title] OR "Women"[Title] OR "human*"[Title] OR "adult*"[Title])))) NOT "Review"[Publication Type]) AND "English"[Language]*

→ results methadone (02-12-24): 10
→ results fentanyl (02-12-24): 21
→ results morphine (02-12-24): 36

To find PK data of methadone, we used the following, standardized, PubMed search queries; one for adults and one for pediatrics. Similar articles and references were checked for any other relevant publications that were not found with the initial search queries.

Adult PK data:

((Pharmacokinet[Title/Abstract]) AND (**drug***[Title] OR **synonym***[Title]) AND (Healthy[Title] OR volunteer*[Title] OR adult*[Title] OR subject*[Title] OR Man[Title] OR Men[Title] OR Woman[Title] OR Women[Title] OR male*[Title] OR female*[Title])) OR (("Pharmacokinetics of"[Title]) AND ("**drug**"[Title] OR **drug***[Title] OR **synonym***[Title]) OR ("**drug** pharmacokinetics"[Title]))*

→ results methadone (synonyms symoron, mephenon, and denolin, 02-12-24): 95
→ results fentanyl (no synonyms, 01-05-2025): 186
→ results morphine (no synonyms, 01-05-2025): 256

Paediatric PK data:
(Pharmacokinet[Title/Abstract]) AND (***drug****[Title] OR* ***synonym****[Title]) AND (Infan*[Title] OR newborn*[Title] OR new-born*[Title] OR perinat*[Title] OR neonat*[Title] OR baby[Title] OR baby*[Title] OR babies[Title] OR prematur*[Title] OR preterm*[Title] OR toddler*[Title] OR minors[Title] OR minors*[Title] OR boy[Title] OR boys[Title] OR boyfriend[Title] OR boyhood[Title] OR girl*[Title] OR kid[Title] OR kids[Title] OR child[Title] OR child*[Title] OR children*[Title] OR schoolchild*[Title] OR schoolchild[Title] OR school child[Title] OR school child*[Title] OR adolescen*[Title] OR juvenil*[Title] OR youth*[Title] OR teen*[Title] OR under*age*[Title] OR pubescen*[Title] OR pediatrics[MeSH] OR pediatric*[Title] OR paediatric*[Title] OR peadiatric*[Title])*
→ results methadone (synonyms symoron, mephenon, and denolin, 02-12-24): 22
→ results fentanyl (no synonyms, 01-05-2025): 65
→ results morphine (no synonyms, 01-05-2025): 90

2.3 Results PBPK modeling

2.3.1 Model verification

The studies used for model verification can be found in 4. Pharmacokinetic studies (Tables F-J). Model verification results of methadone are shown by the predicted-to-observed PK parameter (P-O) ratios (Fig. B) and the visual prediction checks (Appendices 1-3). In adults, 95% of predicted PK parameters for methadone were within 2-fold of the observed PK parameters and for children 8-18 years of age, 90% of all PK parameters were within 2-fold of the observed parameters. For adults receiving a methadone single oral dose (SD PO), the four P-O ratios outside 2-fold were from one study, showing a discrepancy between the individually calculated AUC values and their plasma concentration-time curves (two 'outliers' showing a higher S-methadone plasma-concentration-time curve, with an exposure comparable to R-methadone, cannot be traced back to the table with individual AUC values as all S-methadone AUC values are substantially lower than R-methadone AUC values) (35). For children, the four methadone P-O ratios outside the 2-fold range were also from one clinical study (36). For this study, many assumptions had to be made (i.e., specifics of administered methadone preparation, duration of infusion, timing of C_{max}, timing of C_{min}, exact age of children) due to incomplete reporting, which might have led to a mismatch of our assumptions with reality. For neonates, no PK parameters were known, a visual predictive check can be found in Appendix 3, showing three extreme outliers, all from one patient. These were already evident in the study by Wiles et al. (33), who attributed this to high inter-individual variability, which incidentally was also not captured by their model. Unfortunately, PK data from children between the age of 1 month and 7 years was lacking, so we were unable to verify the model in this sub-population. However, the virtual pediatric population in Simcyp is thoroughly validated over the entire age range, which its justifies extrapolation, as also encouraged by regulatory authorities such as EMA and FDA (37-40). Overall, we concluded that methadone model performance was accurate.

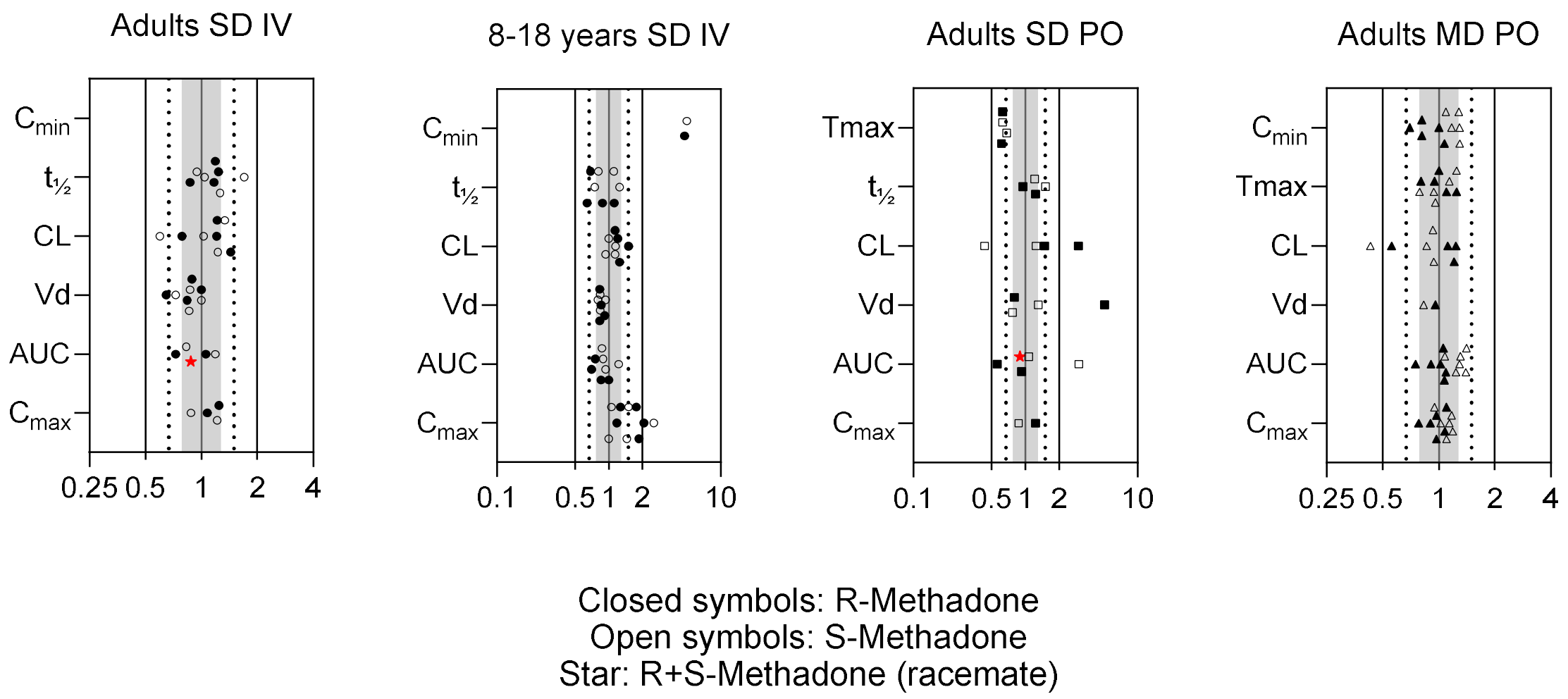

Fig B. Methadone predicted-to-observed PK parameter ratios. The black lines represent the 2-fold range, the dashed lines the 1.5-fold range, the gray shaded area represents the 1.25-fold range and the gray line represents the unity line. Abbreviations: AUC: area under the curve, CL: clearance, C_{max}: maximum concentration, C_{min}: minimum concentration, IV: intravenous, MD: multi-dose, PO: oral, SD: single dose, $t_{½}$: half-life, Vd: volume of distribution.

Model verification of fentanyl was less extensive as fewer PK studies were available to verify the model with. The study by Singleton et al. was used to build the original model, verification with three other adult studies showed some underprediction of CL and Vd (Figure A9) (41-44). The underprediction of adult clearance was also observed by Yao et al. (31) and appears less pronounced in the pediatric population (Figure A10) (44, 45). Vd was not considered by Yao et al. and for model development they used Simcyp to predict this value (31). Compared to the Summary of Product Characteristics, the Vd in the PBPK model is slightly lower (4.07 L/kg compared to 5.7 L/kg), which may explain the somewhat higher predicted fentanyl concentrations. Model predictions in the pediatric population could only be externally verified with the data from Cruzat et al., showing a similar median fentanyl concentration, but also a higher interindividual variability (45).

The PBPK model for morphine was initially developed to characterize the role of the organic cation transporter (OCT1) (30). Unfortunately, they were not able to consider the ontogeny of this transporter, due to lack of knowledge about it. As they had transporter data from children from the age of 6 years, their model was validated in adults and children this age. However, our simulations showed that the model also captured the PK of younger children (Figure A12), only neonatal predictions showed some deviations, mainly an overprediction of CL and Vd (data not shown), resulting in a slight underprediction of morphine plasma concentrations.

2.3.2 Model simulations

Simulations of methadone in various age groups showed differences in methadone exposure (expressed as AUC_{ss}, Figure C), although the exposure in children aged 1-18 years appear similar. Therefore, we merged the age groups of 1 to 18 years. As the exposure in 1-18-year-olds is smallest, with also the lowest C_{max}, we used this age group for comparison of different conversion ratios and tapering schedules to ensure that all age groups would reach the minimum effective methadone concentration of 60 ng/mL.

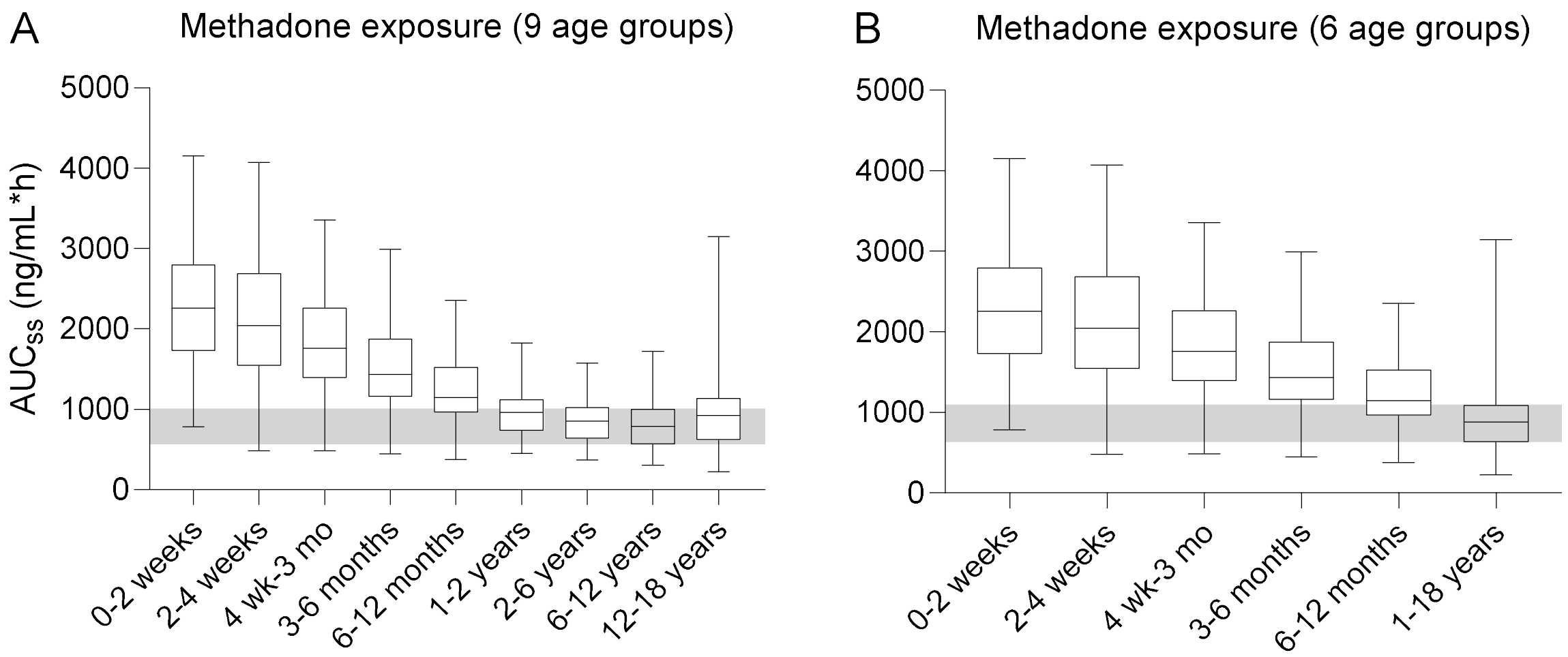

Fig C. Exposure of methadone in various age groups. On the left, methadone AUC_{ss} values in the initial age groups and on the right, methadone AUC_{ss} values in the merged age groups. Boxes indicate the median prediction with the 25th and 75th percentile ranges, whiskers indicate the predicted min and max values of the simulated age groups. The gray horizontal bar represents the AUC of the 6-12-year-olds (left) or 1-18-year-olds (right). AUC_{ss}: area under the curve at steady state.

2.3.3 Model predictions

Simulations of 1 µg/kg/h fentanyl and 10 µg/kg/h morphine converted according to various conversion ratios are depicted in Figure 2 (original manuscript) and Figure D-G, showing that for fentanyl, a 1:10 conversion is most appropriate and for morphine a 1:1 conversion (Figure 2). These conversion ratios were subsequently used to explore the age related differences in drug exposure (Figure 4). In addition, we also simulated a 1 µg/kg/h fentanyl infusion, a 10 µg/kg/h morphine infusion, and the low-risk methadone tapering schedule in different age groups (Figure H).

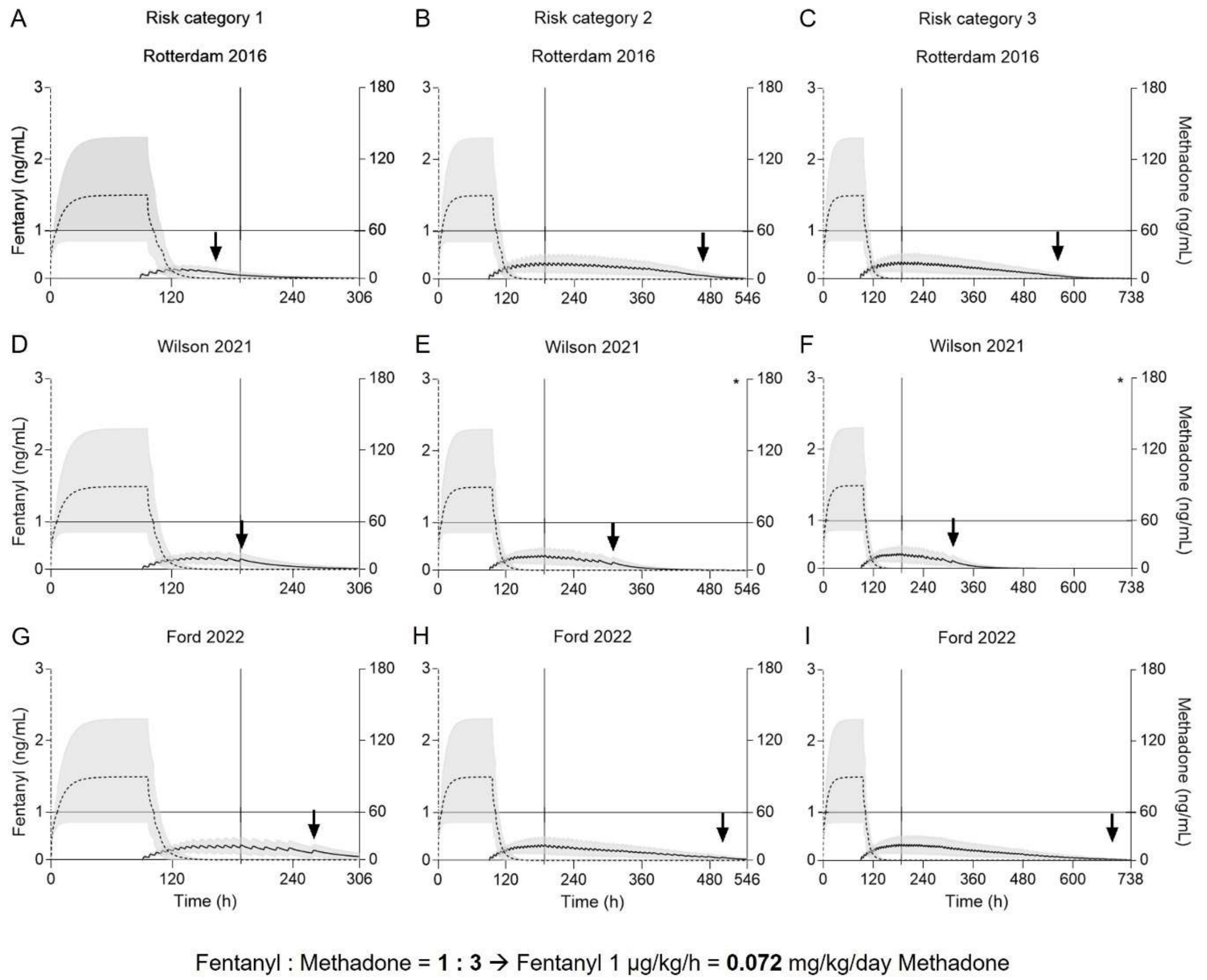

Fig D. Simulations of a 1:3 conversion of fentanyl to methadone, tapered according to three tapering schedules. Fentanyl 1 µg/kg/h is converted into 0.072 mg/kg/day methadone. Each row represents a tapering protocol, each column a withdrawal risk category. The left (dotted) y-axis represents the fentanyl concentration, the right y-axis the methadone concentration. The dotted line is the mean predicted plasma concentration of fentanyl, the bold black line is the mean predicted plasma concentration of methadone. The gray areas are the 5th to 95th percentile of these predictions. The black horizontal line represents the minimal analgesic concentration of fentanyl (1 ng/mL) and the minimal methadone concentration needed to avoid withdrawal symptoms (60 ng/mL). To compare the timing of C_{max}, a black vertical line at t=88 hours after the initiation of methadone administration was drawn, with the black arrows indicating the last administered dose. The methadone tapering schedules are simulated in 1-18-year-olds. *Similar tapering schedule.

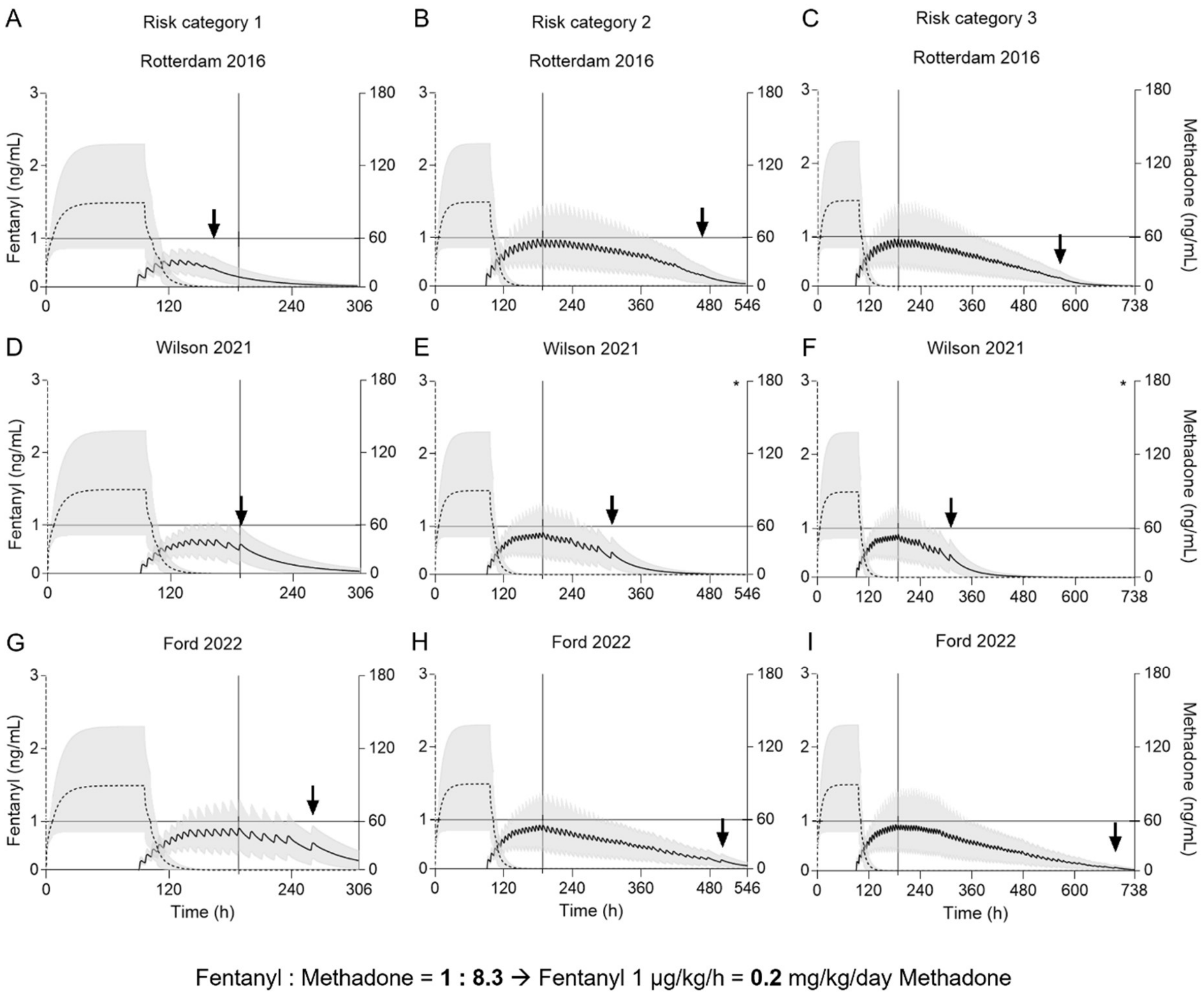

Fig E. Simulations of a 1:8.33 conversion of fentanyl to methadone, tapered according to three tapering schedules. Fentanyl 1 µg/kg/h is converted into 0.2 mg/kg/day methadone. Each row represents a tapering protocol, each column a withdrawal risk category. The left (dotted) y-axis represents the fentanyl concentration, the right y-axis the methadone concentration. The dotted line is the mean predicted plasma concentration of fentanyl, the bold black line is the mean predicted plasma concentration of methadone. The gray areas are the 5th to 95th percentile of these predictions. The black horizontal line represents the minimal analgesic concentration of fentanyl (1 ng/mL) and the minimal methadone concentration needed to avoid withdrawal symptoms (60 ng/mL). To compare the timing of C_{max}, a black vertical line at t=88 hours after the initiation of methadone administration was drawn, with the black arrows indicating the last administered dose. The methadone tapering schedules are simulated in 1-18-year-olds. *Similar tapering schedule.

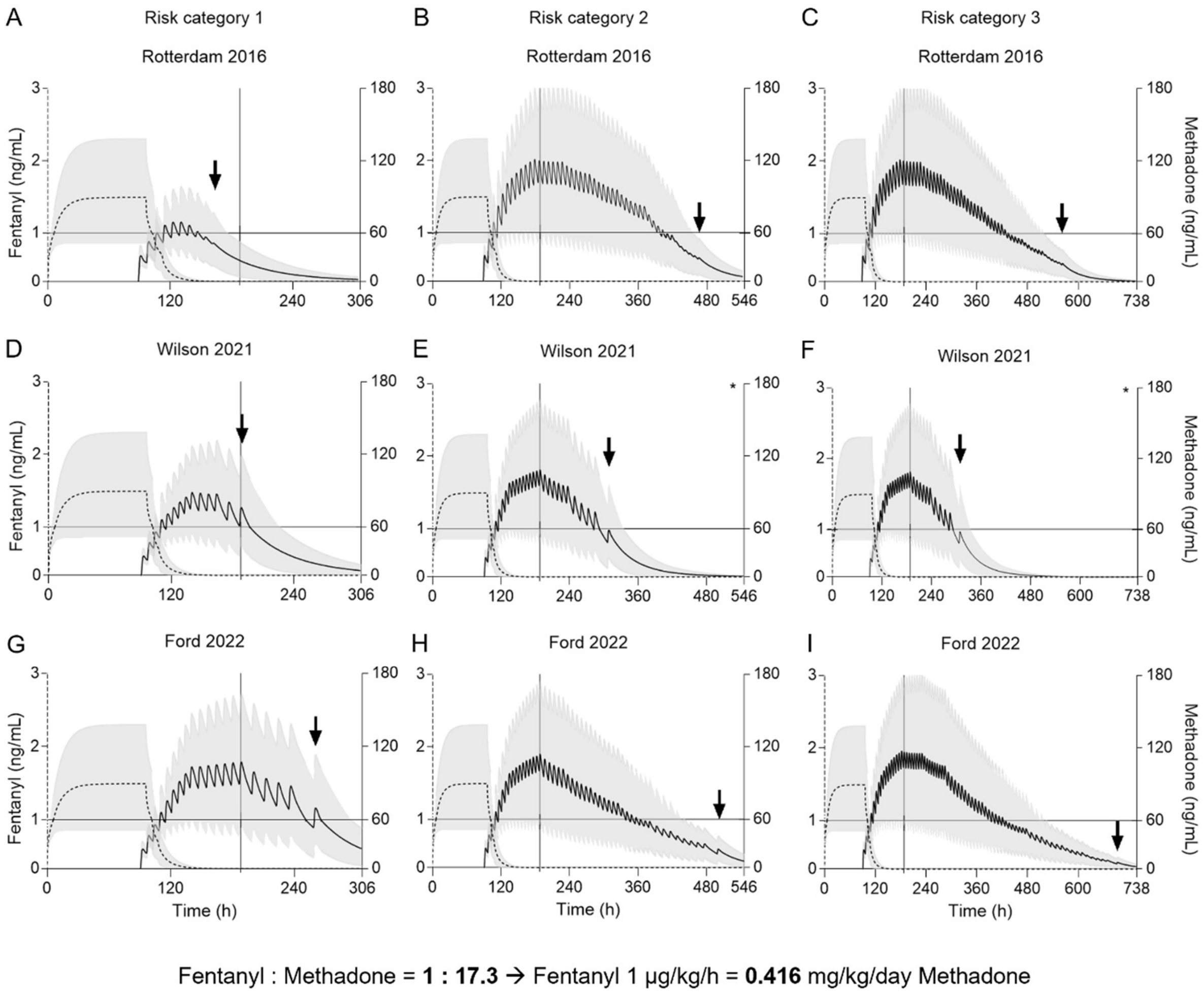

Fig F. Simulations of a 1:17.33 conversion of fentanyl to methadone, tapered according to three tapering schedules. Fentanyl 1 µg/kg/h is converted into 0.4 mg/kg/day methadone. Each row represents a tapering protocol, each column a withdrawal risk category. The left (dotted) y-axis represents the fentanyl concentration, the right y-axis the methadone concentration. The dotted line is the mean predicted plasma concentration of fentanyl, the bold black line is the mean predicted plasma concentration of methadone. The gray areas are the 5th to 95th percentile of these predictions. The black horizontal line represents the minimal analgesic concentration of fentanyl (1 ng/mL) and the minimal methadone concentration needed to avoid withdrawal symptoms (60 ng/mL). To compare the timing of C_{max}, a black vertical line at t=88 hours after the initiation of methadone administration was drawn, with the black arrows indicating the last administered dose. The methadone tapering schedules are simulated in 1-18-year-olds. *Similar tapering schedule.

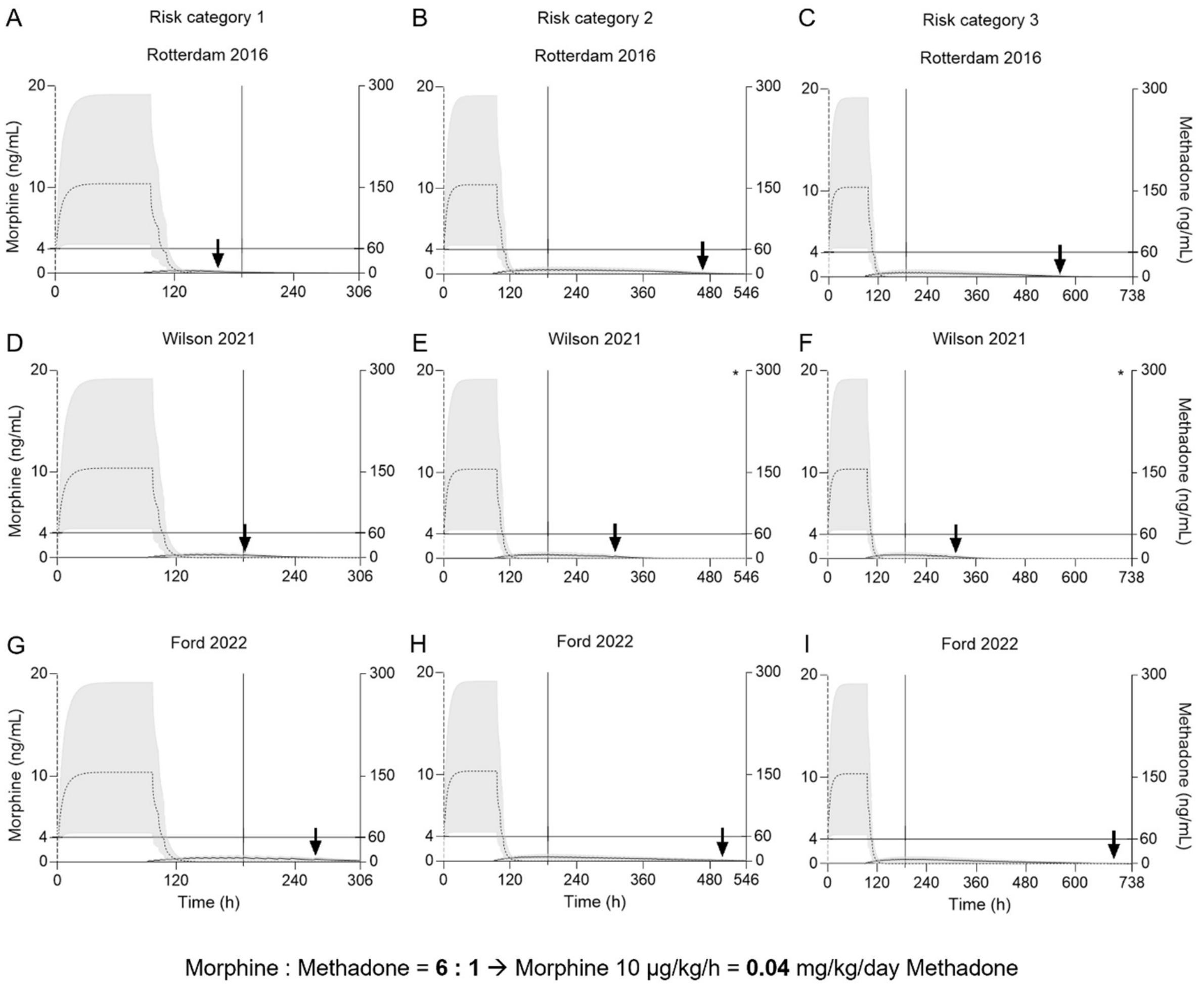

Fig G. Simulations of a 6:1 conversion of morphine to methadone, tapered according to three tapering schedules. Morphine 10 µg/kg/h is converted into 0.04 mg/kg/day methadone. Each row represents a tapering protocol, each column a withdrawal risk category. The left (dotted) y-axis represents the morphine concentration, the right y-axis the methadone concentration. The dotted line is the mean predicted plasma concentration of morphine, the bold black line is the mean predicted plasma concentration of methadone. The gray areas are the 5th to 95th percentile of these predictions. The black horizontal line represents the minimal analgesic concentration of morphine (4 ng/mL) and the minimal methadone concentration needed to avoid withdrawal symptoms (60 ng/mL). To compare the timing of C_{max}, a black vertical line at t=88 hours after the initiation of methadone administration was drawn, with the black arrows indicating the last administered dose. The methadone tapering schedules are simulated in 1-18-year-olds. *Similar tapering schedule.

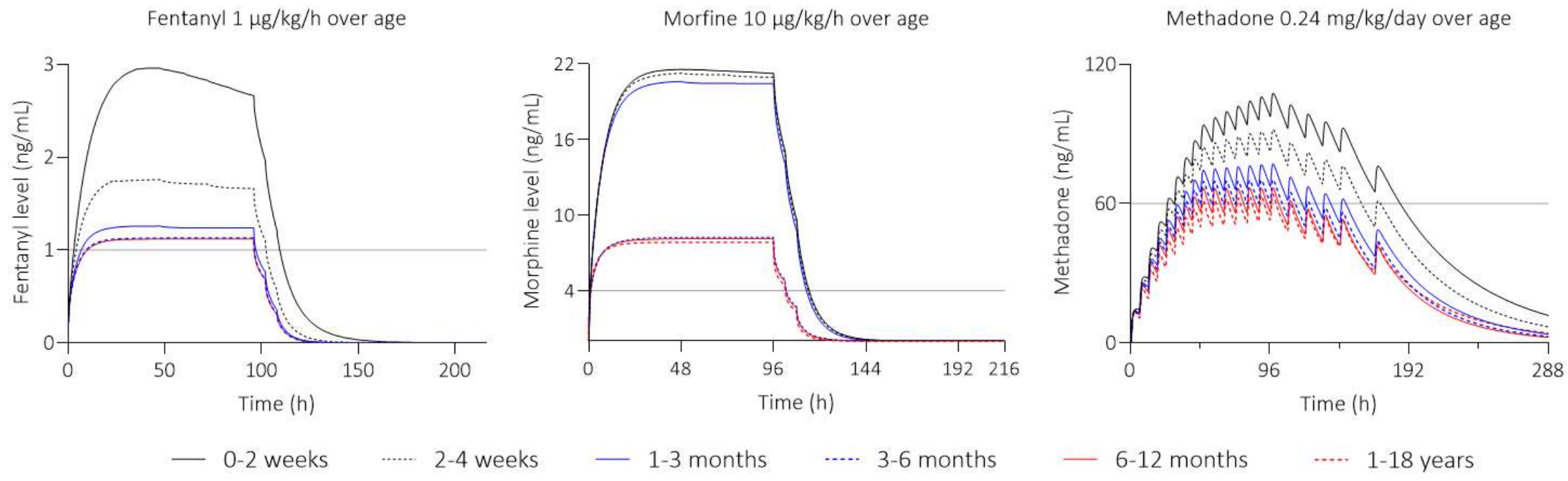

Fig H. Age related differences in drug exposure. Mean plasma concentration-time curves of fentanyl (left), morphine (middle), and methadone (right) are simulated in six different age groups (0-2 and 2-4 weeks postnatal, 4 weeks to 3 months, 3-6 and 6-12 months, and 1-18 years). The horizontal line represents the minimal therapeutic drug concentration.

2.4 Pharmacokinetic studies

Table F. Methadone PK studies in adults

Study	Design	N	Dose	Administration	Health status	Age range (years)	Comed.	Fasted /fed	Prop. of females	Trial duration
IV										
Horst 2016 (46)	SD	11	0.1-0.125 mg/kg	Bolus	Pain in SCD	24.5 ± 6.3	Morphine or hydromorphone	na	0.33	96 h
Kharasch 2009 (47)	SD	12	5.4 mg	IV, *ns*	Healthy	18-34	DDI study	Fasted	0.5	96 h
Kristensen 1996 (48)	SD	7	10-30 mg	IV	Pain	46-76	Morphine a.o.	na	0.29	120 h
Meresaar 1981 (49)	SD	8	20 mg	IV bolus	Addicts	21-29	*ns*	Fasted	0.125	48 h
PO										
Benmebarek 2004 (50)	MD	8	63-150 mg	PO	Addicts	21-45	Benzodiapines a.o.	Fasted	0.125	22 h
Boulton 2001 (35)	SD	8	0.2 mg/kg	PO	Healthy	22-34	No	Fasted	1	96 h
Crauwels 2013 (51)	MD	13	60-100 mg q24h	PO, *ns*	Addicts	31-54	*ns*	Fed	0	24 h
Foster 2000 (52)	MD	18	0.12-1.9 mg/kg	Syrup	Addicts	21-45	Benzodiazepines	*ns*	0.39	24 h
Friedland 2005 (53)	MD	16	*ns*	Oral solution	Addicts	31-57	*ns*	Fed	0.39	24 h
Hendrix 2004 (54)	MD	16	81.2 ± 15.3 mg	PO solution HCl	Addicts	40.3 ± 6.3	No	Fasted	0.5	24 h
Hulskotte 2015 (55)	MD	10	20-150 mg/day	Tablet/liquid	Addicts	34.2 ± 10.2	*ns*	Standardized breakfast	0.2	24 h
Kharasch 2009 (47)	SD	12	9.86 mg	PO, *ns*	Healthy	18-34	DDI study	Fasted	0.5	96 h
Kristensen 1996 (48)	SD	7	10-60 mg	PO	Pain	46-76	Morphine a.o.	*ns*, fasted	0.29	120 h
Meresaar 1981 (49)	SD	8	20 mg	PO tablet	Addicts	21-29	*ns*	Fasted	0.125	48 h

Studies in *Italic* were used to build and/or verify the model (28, 56). Abbreviations: a.o.: amongst others, IV: intravenous, MD: multi dose, na: not applicable, *ns*: not specified, PO: oral, qXh: every X hours, SCD: sickle cell disease, SD: single dose

Table G. Methadone PK studies in children

Study	Design	N	Dose	Administration	Health status	Age range	Comed.	Fasted /fed	Prop. of females	Trial duration
IV										
Horst 2016 (46)	SD	12	0.1-0.125 mg/kg	Infusion 10 min	Pain in SCD	14.5 ± 2.3 y	Morphine or hydromorphone	na	0.25	96 h
Sadhasivam 2021 (36)	SD	38	0.1 mg/kg	IV	Intraoperative PE/PSF	15.6 (IQR 13.6-16.3)/ 14.2 (IQR 12.8-16)	Oxycodone, hydromorphone, morphine	na	0.47	54 h
Sharma 2011 (57)	SD	31	0.1 mg/kg<br>0.2 mg/kg<br>0.3 mg/kg, max 20 mg	IV	Perioperative	10-18 y	Anesthetics, pain medication	na	0.8<br>0.4<br>0.82	96 h
Stemland 2012 (58)	SD	17	0.25 mg/kg	IV	Pre-operative	12-15 y	Anesthetics	na	0.88	48 h
PO										
Sadhasivam 2021 (36)	MD	38	0.1 mg/kg q12h	PO, *ns*	Postoperative PE/PSF	15.6 (IQR 13.6-16.3)/ 14.2 (IQR 12.8-16)	Oxycodone, hydromorphone, morphine	*ns*, fed	0.47	54 h
Wiles 2015 (33)	*SD*	*20*	*0.05 mg/kg*	*PO, ns*	*NAS*	*1-20 d PNA, median 38 wk GA, (IQR 37.4-39.5)*	*ns*	*ns, fed*	*0.55*	*8 h*

Studies in *Italic* were used to build and/or verify the model (28). Abbreviations: d: day, GA: gestational age, IQR: inter quartile range, IV: intravenous, MD: multi dose, na: not applicable, NAS: neonatal abstinence syndrome, *ns*: not specified, PE: pectus excavatum, PNA: post-natal age, PO: oral, PSF: posterior spinal fusion, qXh: every X hours, SCD: sickle cell disease, SD: single dose, wk: week, y: year

Table H. Fentanyl PK studies

Study	Design	N	Dose	Administration	Health status	Age range	Comed.	Prop. of females	Trial duration
Fentanyl IV - adults									
Duthie 1986 (41)	MD	10	100 µg bolus, 100 µg/h CI	IV bolus, time *ns*, CI 24 hours	Orthopedic surgery	61 ± 8 y	Other anesthetics	*ns*	28 h
		13	100 µg bolus, 100 µg/h CI	IV bolus, time *ns*, CI 24 hours	Abdominal surgery	49 ± 14 y	Other anesthetics	*ns*	28 h
Singleton 1987 (44)	SD	7	Corrected for 1 µg/kg	IV 2 min bolus	Elective non-cardiac surgery	18-41 y	Other anesthetics	*ns*	4 h
Saari 2008 (42)	SD	12	5 µg/kg	IV 2 min bolus	Healthy volunteers	Young adults	No	0.58	24 h
Sandler 1992 (43)	MD	16	1.5 µg/kg bolus,<br>CI of 1-1.6 µg/kg/h	IV bolus, time *ns*, CI ± 24 h	Elective thoracotomy	61 ± 13 y	Other anesthetics	0.5	24 h
Fentanyl IV - children									
Cruzat 2024 (45)	SD	19	2 µg/kg	IV bolus, time *ns*	Elective surgery	3-10 y	Other anesthetics	0.95	2.33 h
Singleton 1987 (44)	SD	7<br>7<br>7	Corrected for 1 µg/kg	IV 2 min bolus	Elective non-cardiac surgery	3-10 mo<br>1-9 y<br>18-41 y	Other anesthetics	*ns*	4 h

Studies in *Italic* were used to build and/or verify the models (30, 31). Abbreviations: CI: continuous infusion, h: hours, IV: intravenous, MD: multi dose, min: minutes, mo: months, ns: not specified, SD: single dose, wk: week, y: year

Table I. Morphine PK studies in adults

Study	Design	N	Dose	Administration	Health status	Age range	Comed.	Prop. of females	Trial duration
Morphine IV - adults									
Crews 2001 (59)	SD	12	10 mg (Sulphate) = 7.44 mg morphine	IV 5 min bolus	Healthy volunteers	25.8 ± 8.8 y	No	0.5	24 h
Dershwitz 2000 (60)	SD	15	8.8 mg (unknown salt) → 6.6 mg morphine (assumption)	IV 7 min infusion	Healthy volunteers	22-45 y	No	0.33	4.3 h
Duflot 2021 (61)	SD	14	1-5 mg, dose normalized to 1 mg (assumption) (unknown salt) → 0.75 mg	IV 1 min bolus	Healthy volunteers	18-60 y	No	0.5	5.08 h
Hanna 1991 (62)	SD	6	120 µg/kg (Sulphate) = 89.31 µg/kg morphine	IV 4 min bolus	Healthy volunteers	30.6 ± 2.5 y	No	0.5	6 h
Khalili-Mahani 2012 (63)	CI	12	100 µg/kg/h bolus, 30 µg/kg/h CI (unknown salt) → 75 µg/kg/h bolus, 22.5 µg/kg/h CI	IV 1 min bolus, CI 2.5 h	Healthy volunteers	18-40 y	No	0	2.5 h
Lötsch 1996 (64)	CI	4	130 µg/kg (HCL) bolus (= 99 µg/kg), 5 (= 3.8) µg/kg/h CI	IV 5 min bolus, CI 8 h	Healthy volunteers	23-29 y	No	0	12 h
		4	240 µg/kg (HCL) bolus (= 180 µg/kg), 69 (= 52) µg/kg/h CI	IV 5 min bolus, CI 4 h					10 h
Morphine IV - children									

Unknown which studies were used to build and/or verify the model (30). IV morphine solutions are usually HCL or sulphate salts, with a molecular weight of 375.9 g/mol or 383.4 g/mol, respectively. In case it is not mentioned what formulation was used, the mean of these two weights were used to convert the dose to morphine base. Abbreviations: CI: continuous infusion, h: hours, HCL: hydrochloride, IV: intravenous, min: minutes, ns: not specified, SD: single dose, y: year

Table J. Morphine PK studies in children

Study	Design	N	Dose	Administration	Health status	Age range	Comed.	Prop. of females	Trial duration
Bouwmeester 2003 (65)	CI	31<br>32<br>16<br>18	100 µg/kg (HCL) = 76 µg/kg morphine,<br>10-12.3 µg/kg/h maintenance	IV 2 min bolus, then CI	Non-cardiac thoracic and abdominal surgery	0-4 wk<br>1-6 mo<br>6-12 mo<br>1-3 y	Other anesthetics	0.41<br>0.35<br>0.40<br>0.54	24 h
Dahlstrom 1979 (66)	SD	8<br>8<br>1	0.15 mg/kg (HCL) = 0.11 mg/kg morphine<br>0.15 mg/kg (HCL) = 0.11 mg/kg morphine<br>0.15 mg/kg bolus + 0.075 mg/kg bolus	IV bolus, time *ns*	Surgery	1-7 y<br>7-15 y<br>7-15 y	Other anesthetics	0.42<br>0.5<br>0.5	8 h
Dalesio 2020 (67)	SD	18	0.05 mg/kg (HCL) = 0.038 mg/kg morphine	IV bolus, time *ns*	Surgery	5-12 y	Other anesthetics	0.39	9 h
De Hoogd 2021 (68)	CI	35	0.1 mg/kg bolus, 0.031 mg/kg/h from 0-24 h, 0.016 mg/kg/h from 24-48 h (unknown salt) → 0.075 mg/kg bolus, 0.023 and 0.012 mg/kg/h	IV bolus, time *ns*, CI for 48 h	Cardiac surgery with CPB	3-36 mo	Other anesthetics	*ns*	48 h
Jimenez 2012 (69)	SD	68	0.15 mg/kg (unknown salt) → 0.11 mg/kg	IV bolus, time *ns*	Tonsillectomy or adenoid-ectomy	3-17 y	Other anesthetics	0.51	6 h
Pokela 1993 (70)	SD	10<br>10<br>7	0.1 mg/kg (HCL) = 0.076 mg/kg morphine	IV 1 min bolus	Surgery	<1 wk<br>1 wk-2 mo<br>2-6 mo	Other anesthetics	*ns*	12 h<br>8 h<br>6 h

Unknown which studies were used to build and/or verify the model (30). IV morphine solutions are usually HCL or sulphate salts, with a molecular weight of 375.9 g/mol or 383.4 g/mol, respectively. In case it is not mentioned what formulation was used, the mean of these two weights were used to convert the dose to morphine base. Abbreviations: CI: continuous infusion, h: hours, HCL: hydrochloride, IV: intravenous, min: minutes, mo: months, ns: not specified, SD: single dose, wk: week, y: year

2.5 References

1. Abdouni R, Reyburn-Orne T, Youssef TH, et al.: Impact of a Standardized Treatment Guideline for Pediatric Iatrogenic Opioid Dependence: A Quality Improvement Initiative. *J Pediatr Pharmacol Ther* 2016; 21:54-65

2. Amirnovin R, Sanchez-Pinto LN, Okuhara C, et al.: Implementation of a Risk-Stratified Opioid and Benzodiazepine Weaning Protocol in a Pediatric Cardiac ICU. *Pediatr Crit Care Med* 2018; 19:1024-1032

3. Berens RJ, Meyer MT, Mikhailov TA, et al.: A prospective evaluation of opioid weaning in opioid-dependent pediatric critical care patients. *Anesth Analg* 2006; 102:1045-1050

4. Bowens CD, Thompson JA, Thompson MT, et al.: A trial of methadone tapering schedules in pediatric intensive care unit patients exposed to prolonged sedative infusions. *Pediatr Crit Care Med* 2011; 12:504-511

5. Curley MA, Wypij D, Watson RS, et al.: Protocolized sedation vs usual care in pediatric patients mechanically ventilated for acute respiratory failure: a randomized clinical trial. *Jama* 2015; 313:379-389

6. Fife A, Postier A, Flood A, et al.: Methadone conversion in infants and children: Retrospective cohort study of 199 pediatric inpatients. *J Opioid Manag* 2016; 12:123-130

7. Ford J, Harthan A, McGarvey J, et al.: Implementation of an Opioid Weaning Protocol at a Tertiary Care Children's Hospital. *Hosp Pediatr* 2022; 12:945-953

8. Friedman SD, Kovach JR, Thompson NE: Methadone's Effect on Cardiac Repolarization: Safety in the PICU. *Pediatr Crit Care Med* 2020; 21:e747-e751

9. Hintze TD, Miller JL, Neely SB, et al.: Characterization of early versus late opioid iatrogenic withdrawal syndrome in critically ill children transitioning from fentanyl -infusions to methadone. *J Opioid Manag* 2023; 19:43-56

10. Jeffries SA, McGloin R, Pitfield AF, et al.: Use of methadone for prevention of opioid withdrawal in critically ill children. *Can J Hosp Pharm* 2012; 65:12-18

11. Johnson PN, Miller J, Harrison D: Evaluation of initial methadone dosing for prevention of iatrogenic opioid abstinence syndrome in children. *J Pediatr Intensive Care* 2012; 1:105-113

12. Johnson MR, Nash DR, Laird MR, et al.: Development and implementation of a pharmacist-managed, neonatal and pediatric, opioid-weaning protocol. *J Pediatr Pharmacol Ther* 2014; 19:165-173

13. Lugo RA, MacLaren R, Cash J, et al.: Enteral methadone to expedite fentanyl discontinuation and prevent opioid abstinence syndrome in the PICU. *Pharmacotherapy* 2001; 21:1566-1573

14. Meyer MM, Berens RJ: Efficacy of an enteral 10-day methadone wean to prevent opioid withdrawal in fentanyl-tolerant pediatric intensive care unit patients. *Pediatr Crit Care Med* 2001; 2:329-333

15. Robertson RC, Darsey E, Fortenberry JD, et al.: Evaluation of an opiate-weaning protocol using methadone in pediatric intensive care unit patients. *Pediatr Crit Care Med* 2000; 1:119-123

16. Sanchez-Pinto LN, Nelson LP, Lieu P, et al.: Implementation of a risk-stratified opioid weaning protocol in a pediatric intensive care unit. *J Crit Care* 2018; 43:214-219

17. Siddappa R, Fletcher JE, Heard AM, et al.: Methadone dosage for prevention of opioid withdrawal in children. *Paediatr Anaesth* 2003; 13:805-810

18. Solodiuk JC, Greco CD, O'Donnell KA, et al.: Effect of a Sedation Weaning Protocol on Safety and Medication Use among Hospitalized Children Post Critical Illness. *J Pediatr Nurs* 2019; 49:18-23

19. Srinivasan V, Pung D, O'Neill SP: Conversion from prolonged intravenous fentanyl infusion to enteral methadone in critically ill children. *World J Clin Pediatr* 2017; 6:110-117

20. Steineck KJ, Skoglund AK, Carlson MK, et al.: Evaluation of a pharmacist-managed methadone taper*. *Pediatr Crit Care Med* 2014; 15:206-210

21. Tiacharoen D, Lertbunrian R, Veawpanich J, et al.: Protocolized Sedative Weaning vs Usual Care in Pediatric Critically Ill Patients: A Pilot Randomized Controlled Trial. *Indian J Crit Care Med* 2020; 24:451-458

22. Tobias JD, Schleien CL, Haun SE: Methadone as treatment for iatrogenic narcotic dependency in pediatric intensive care unit patients. *Crit Care Med* 1990; 18:1292-1293
23. Tobias JD, Deshpande JK, Gregory DF: Outpatient therapy of iatrogenic drug dependency following prolonged sedation in the pediatric intensive care unit. *Intensive Care Med* 1994; 20:504-507
24. Tobias J: Out-patient Therapy of Iatrogenic Opioid Dependency following Prolonged Sedation in the Pediatric Intensive Care Unit. *Journal of Intensive Care Medicine* 1996; 11:284-287
25. Vipond JM, Heiberger AL, Thompson PA, et al.: Shortened Taper Duration after Implementation of a Standardized Protocol for Iatrogenic Benzodiazepine and Opioid Withdrawal in Pediatric Patients: Results of a Cohort Study. *Pediatr Qual Saf* 2018; 3:e079
26. Walters RA, Izquierdo M, Rodriguez JC, et al.: Iatrogenic Opiate Withdrawal in Pediatric Patients: Implementation of a Standardized Methadone Weaning Protocol and Withdrawal Assessment Tool. *J Pharm Pract* 2021; 34:417-422
27. Wilson AK, Ragsdale CE, Sehgal I, et al.: Exposure-Based Methadone and Lorazepam Weaning Protocol Reduces Wean Length in Children. *J Pediatr Pharmacol Ther* 2021; 26:42-49
28. McPhail BT, Emoto C, Fukuda T, et al.: Utilizing Pediatric Physiologically Based Pharmacokinetic Models to Examine Factors That Contribute to Methadone Pharmacokinetic Variability in Neonatal Abstinence Syndrome Patients. *J Clin Pharmacol* 2020; 60:453-465
29. Codaccioni M, Southall RL, Dinh J, et al.: Prediction of Pediatric Pharmacokinetics for CYP3A4 Metabolized Drugs: Comparison of the Performance of Two Hepatic Ontogeny Within a Physiologically Based Pharmacokinetic Model. *J Clin Pharmacol* 2024; 64:1083-1094
30. Emoto C, Fukuda T, Johnson TN, et al.: Characterization of Contributing Factors to Variability in Morphine Clearance Through PBPK Modeling Implemented With OCT1 Transporter. *CPT Pharmacometrics Syst Pharmacol* 2017; 6:110-119
31. Yao X, Liu X, Tu S, et al.: Development of a Virtual Chinese Pediatric Population Physiological Model Targeting Specific Metabolism and Kidney Elimination Pathways. *Front Pharmacol* 2021; 12:648697
32. van der Heijden JEM, Freriksen JJM, de Hoop-Sommen MA, et al.: Physiologically-Based Pharmacokinetic Modeling for Drug Dosing in Pediatric Patients: A Tutorial for a Pragmatic Approach in Clinical Care. *Clin Pharmacol Ther* 2023; 114:960-971
33. Wiles JR, Isemann B, Mizuno T, et al.: Pharmacokinetics of Oral Methadone in the Treatment of Neonatal Abstinence Syndrome: A Pilot Study. *J Pediatr* 2015; 167:1214-1220.e1213
34. Erasmus MC: Sedativa/opiaten, afbouwen bij kinderen. Versie 2 (30-05-2016).
35. Boulton DW, Arnaud P, DeVane CL: Pharmacokinetics and pharmacodynamics of methadone enantiomers after a single oral dose of racemate. *Clin Pharmacol Ther* 2001; 70:48-57
36. Sadhasivam S, Aruldhas BW, Packiasabapathy S, et al.: A Novel Perioperative Multidose Methadone-Based Multimodal Analgesic Strategy in Children Achieved Safe and Low Analgesic Blood Methadone Levels Enabling Opioid-Sparing Sustained Analgesia With Minimal Adverse Effects. *Anesth Analg* 2021; 133:327-337
37. EMA: ICH E11(R1) guideline on clinical investigation of medicinal products in the pediatric population. Available at: https://www.ema.europa.eu/en/documents/scientific-guideline/ich-e11r1-guideline-clinical-investigation-medicinal-products-pediatric-population-revision-1-addendum_en.pdf. Accessed
38. EMA: ICH E11A Guideline on pediatric extrapolation. Step 5 Available at: https://www.ema.europa.eu/en/documents/scientific-guideline/ich-guideline-e11a-pediatric-extrapolation-step-5_en.pdf. Accessed
39. FDA: Physiologically Based Pharmacokinetic Analyses — Format and Content; Guidance for Industry. 2018
40. Johnson TN, Rostami-Hodjegan A, Tucker GT: Prediction of the clearance of eleven drugs and associated variability in neonates, infants and children. *Clin Pharmacokinet* 2006; 45:931-956
41. Duthie DJ, McLaren AD, Nimmo WS: Pharmacokinetics of fentanyl during constant rate i.v. infusion for the relief of pain after surgery. *Br J Anaesth* 1986; 58:950-956

42. Saari TI, Laine K, Neuvonen M, et al.: Effect of voriconazole and fluconazole on the pharmacokinetics of intravenous fentanyl. *Eur J Clin Pharmacol* 2008; 64:25-30
43. Sandler AN, Stringer D, Panos L, et al.: A randomized, double-blind comparison of lumbar epidural and intravenous fentanyl infusions for postthoracotomy pain relief. Analgesic, pharmacokinetic, and respiratory effects. *Anesthesiology* 1992; 77:626-634
44. Singleton MA, Rosen JI, Fisher DM: Plasma concentrations of fentanyl in infants, children and adults. *Can J Anaesth* 1987; 34:152-155
45. Cruzat F, Ibacache M, González A, et al.: Assessment of the antinociceptive effect of a single fentanyl bolus dose in children: A pharmacokinetic and pharmacodynamic analysis based on the nociception level index during sevoflurane general anesthesia. *Paediatr Anaesth* 2024; 34:1258-1266
46. Horst J, Frei-Jones M, Deych E, et al.: Pharmacokinetics and analgesic effects of methadone in children and adults with sickle cell disease. *Pediatr Blood Cancer* 2016; 63:2123-2130
47. Kharasch ED, Hoffer C, Whittington D, et al.: Methadone pharmacokinetics are independent of cytochrome P4503A (CYP3A) activity and gastrointestinal drug transport: insights from methadone interactions with ritonavir/indinavir. *Anesthesiology* 2009; 110:660-672
48. Kristensen K, Blemmer T, Angelo HR, et al.: Stereoselective pharmacokinetics of methadone in chronic pain patients. *Ther Drug Monit* 1996; 18:221-227
49. Meresaar U, Nilsson MI, Holmstrand J, et al.: Single dose pharmacokinetics and bioavailability of methadone in man studied with a stable isotope method. *Eur J Clin Pharmacol* 1981; 20:473-478
50. Benmebarek M, Devaud C, Gex-Fabry M, et al.: Effects of grapefruit juice on the pharmacokinetics of the enantiomers of methadone. *Clin Pharmacol Ther* 2004; 76:55-63
51. Crauwels HM, van Heeswijk RP, Vandevoorde A, et al.: The effect of rilpivirine on the pharmacokinetics of methadone in HIV-negative volunteers. *J Clin Pharmacol* 2014; 54:133-140
52. Foster DJ, Somogyi AA, Dyer KR, et al.: Steady-state pharmacokinetics of (R)- and (S)-methadone in methadone maintenance patients. *Br J Clin Pharmacol* 2000; 50:427-440
53. Friedland G, Andrews L, Schreibman T, et al.: Lack of an effect of atazanavir on steady-state pharmacokinetics of methadone in patients chronically treated for opiate addiction. *Aids* 2005; 19:1635-1641
54. Hendrix CW, Wakeford J, Wire MB, et al.: Pharmacokinetics and pharmacodynamics of methadone enantiomers after coadministration with amprenavir in opioid-dependent subjects. *Pharmacotherapy* 2004; 24:1110-1121
55. Hulskotte EG, Bruce RD, Feng HP, et al.: Pharmacokinetic interaction between HCV protease inhibitor boceprevir and methadone or buprenorphine in subjects on stable maintenance therapy. *Eur J Clin Pharmacol* 2015; 71:303-311
56. Ke AB, Nallani SC, Zhao P, et al.: Expansion of a PBPK model to predict disposition in pregnant women of drugs cleared via multiple CYP enzymes, including CYP2B6, CYP2C9 and CYP2C19. *Br J Clin Pharmacol* 2014; 77:554-570
57. Sharma A, Tallchief D, Blood J, et al.: Perioperative pharmacokinetics of methadone in adolescents. *Anesthesiology* 2011; 115:1153-1161
58. Stemland CJ, Witte J, Colquhoun DA, et al.: The pharmacokinetics of methadone in adolescents undergoing posterior spinal fusion. *Paediatr Anaesth* 2013; 23:51-57
59. Crews KR, Murthy BP, Hussey EK, et al.: Lack of effect of ondansetron on the pharmacokinetics and analgesic effects of morphine and metabolites after single-dose morphine administration in healthy volunteers. *Br J Clin Pharmacol* 2001; 51:309-316
60. Dershwitz M, Walsh JL, Morishige RJ, et al.: Pharmacokinetics and pharmacodynamics of inhaled versus intravenous morphine in healthy volunteers. *Anesthesiology* 2000; 93:619-628
61. Duflot T, Pereira T, Tavolacci MP, et al.: Pharmacokinetic modeling of morphine and its glucuronides: Comparison of nebulization versus intravenous route in healthy volunteers. *CPT Pharmacometrics Syst Pharmacol* 2022; 11:82-93
62. Hanna MH, Peat SJ, Knibb AA, et al.: Disposition of morphine-6-glucuronide and morphine in healthy volunteers. *Br J Anaesth* 1991; 66:103-107

63. Khalili-Mahani N, Zoethout RM, Beckmann CF, et al.: Effects of morphine and alcohol on functional brain connectivity during "resting state": a placebo-controlled crossover study in healthy young men. *Hum Brain Mapp* 2012; 33:1003-1018
64. Lötsch J, Stockmann A, Kobal G, et al.: Pharmacokinetics of morphine and its glucuronides after intravenous infusion of morphine and morphine-6-glucuronide in healthy volunteers. *Clin Pharmacol Ther* 1996; 60:316-325
65. Bouwmeester NJ, van den Anker JN, Hop WC, et al.: Age- and therapy-related effects on morphine requirements and plasma concentrations of morphine and its metabolites in postoperative infants. *Br J Anaesth* 2003; 90:642-652
66. Dahlström B, Bolme P, Feychting H, et al.: Morphine kinetics in children. *Clin Pharmacol Ther* 1979; 26:354-365
67. Dalesio NM, Lee CKK, Hendrix CW, et al.: Effects of Obstructive Sleep Apnea and Obesity on Morphine Pharmacokinetics in Children. *Anesth Analg* 2020; 131:876-884
68. de Hoogd S, Goulooze SC, Valkenburg AJ, et al.: Postoperative breakthrough pain in paediatric cardiac surgery not reduced by increased morphine concentrations. *Pediatr Res* 2021; 90:1201-1206
69. Jimenez N, Anderson GD, Shen DD, et al.: Is ethnicity associated with morphine's side effects in children? Morphine pharmacokinetics, analgesic response, and side effects in children having tonsillectomy. *Paediatr Anaesth* 2012; 22:669-675
70. Pokela ML, Olkkola KT, Seppälä T, et al.: Age-related morphine kinetics in infants. *Dev Pharmacol Ther* 1993; 20:26-34

Appendix 1. Model verification; methadone in adults

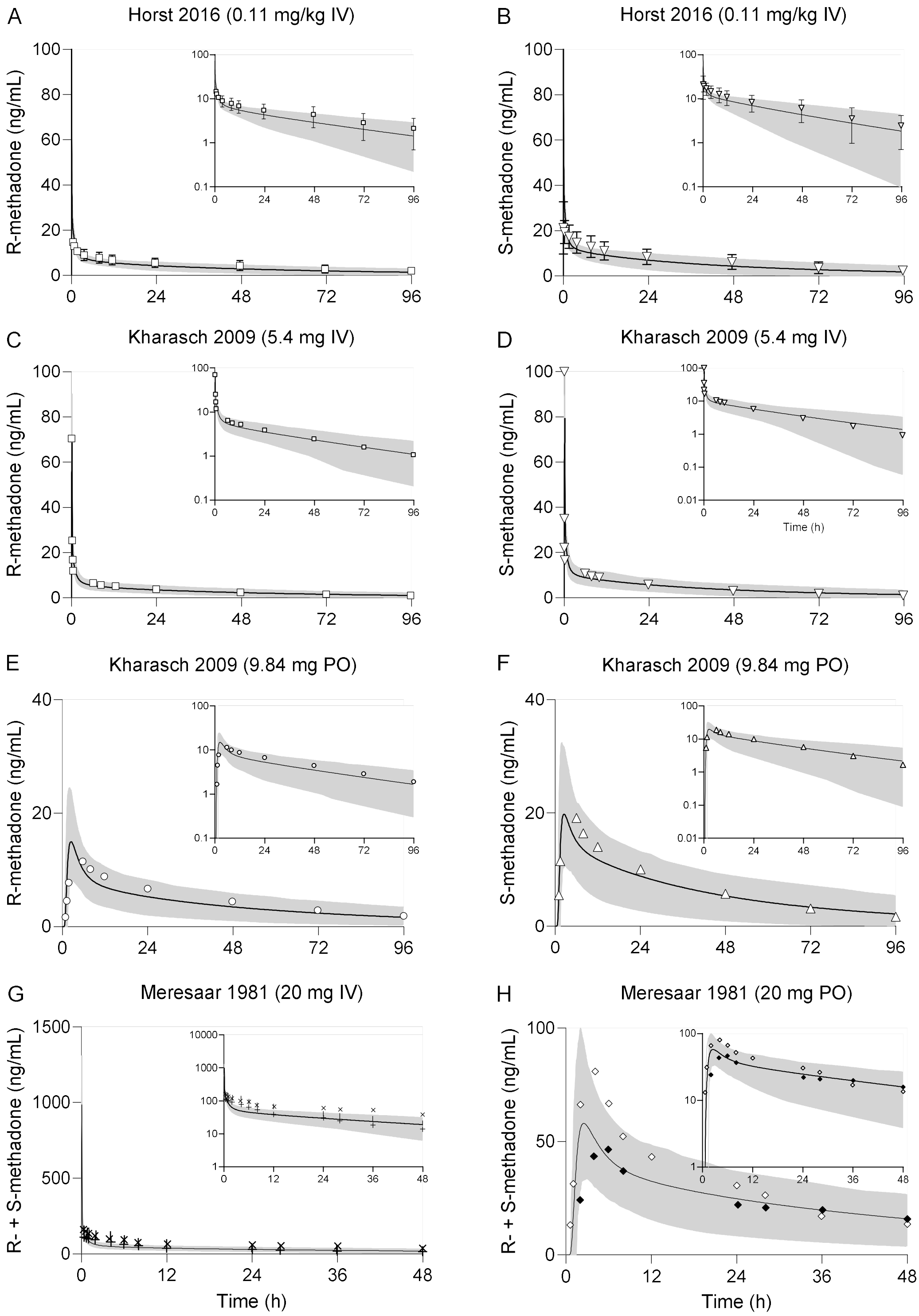

Figure A1. Visual predictive checks for methadone in adults (single dose, IV and PO). The solid line is the predicted mean of the simulated population and the shaded area represents the 5th to 95th percentile of the virtual population. In graphs A-F, symbols are mean observed datapoints (± standard deviation), open squares and open triangles up-side down are IV administrations, open circles and open triangles are PO administrations. (46, 47) Symbols in graphs G and H are individual data points. (49) Inserts show semi-log plots. R-methadone concentrations on the left (A, C, and E), S-methadone concentrations on the right (B, D, and F), and racemate methadone concentrations in graphs G and H. Abbreviations: IV: intravenous, PO: oral.

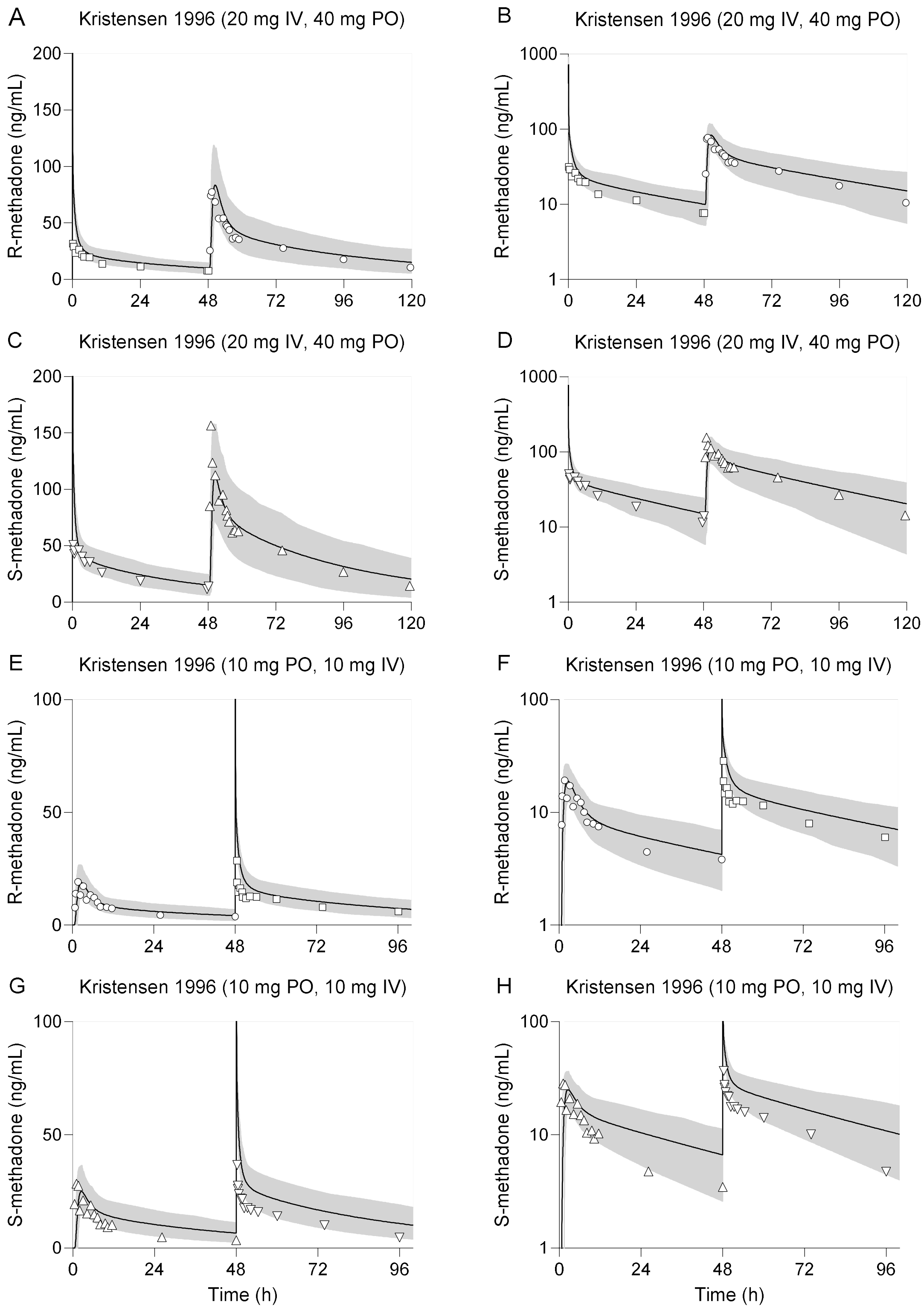

Figure A2. Visual predictive checks for methadone in adults (two doses, one IV and one PO). The solid line is the predicted mean of the simulated population and the shaded area represents the 5th to 95th percentile of the virtual population. Symbols are mean observed datapoints, open squares and open triangles up-side down are IV administrations, open circles and open triangles are PO administrations. (48) R-methadone concentrations on the left (A, C, E, and G), S-methadone concentrations on the right (B, D, F, and H). Abbreviations: IV: intravenous, PO: oral.

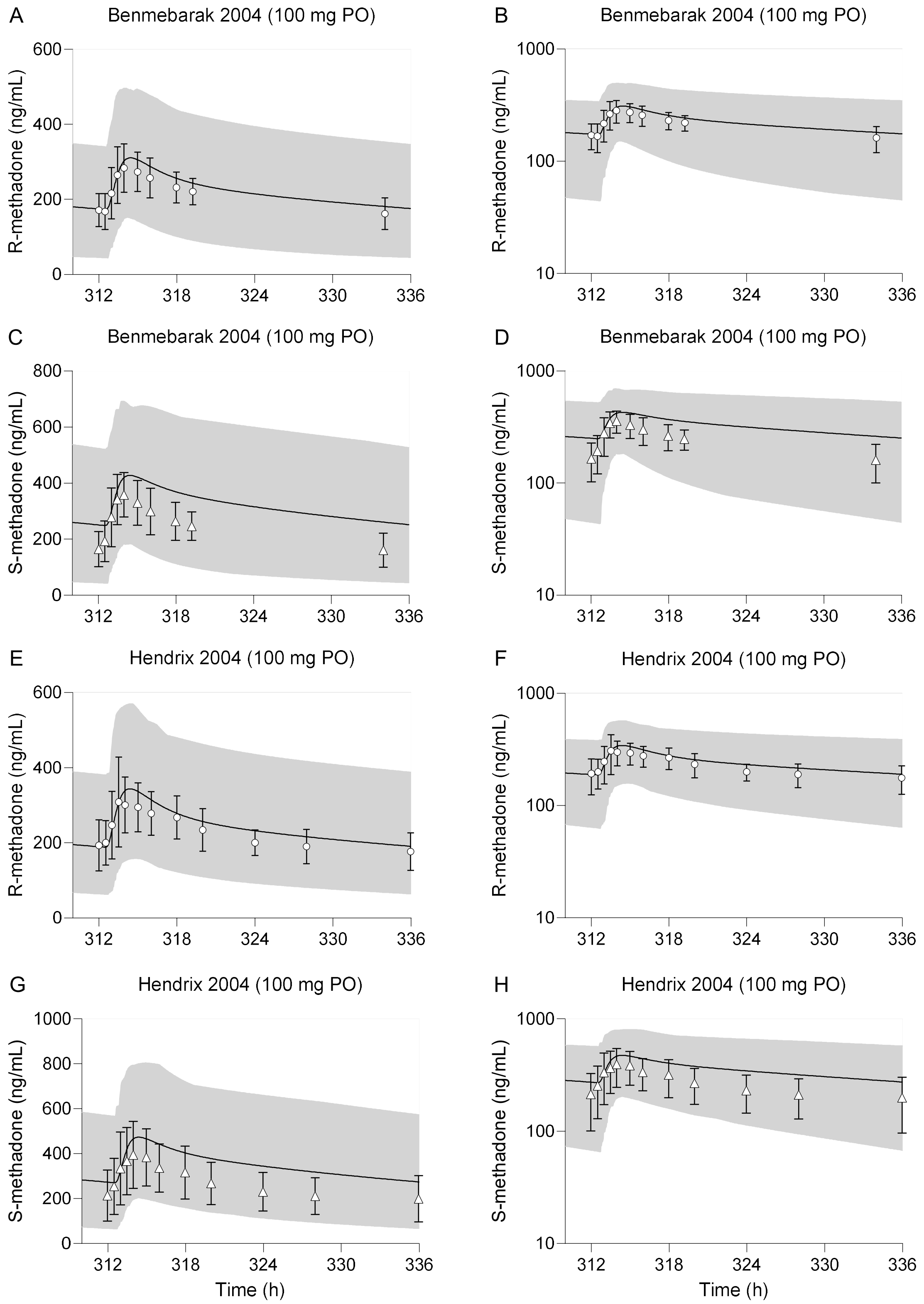

Figure A3. Visual predictive checks for methadone in adults (multidose, PO). The solid line is the predicted mean of the simulated population and the shaded area represents the 5th to 95th percentile of the virtual population. Symbols are observed datapoints (mean ± standard deviation), open circles are R-methadone concentrations and open triangles are S-methadone concentrations. (50, 54) On the right side (graphs B, D, F, and H) are semi-log plots of graphs A, C, E, and G, respectively. Abbreviations: PO: oral.

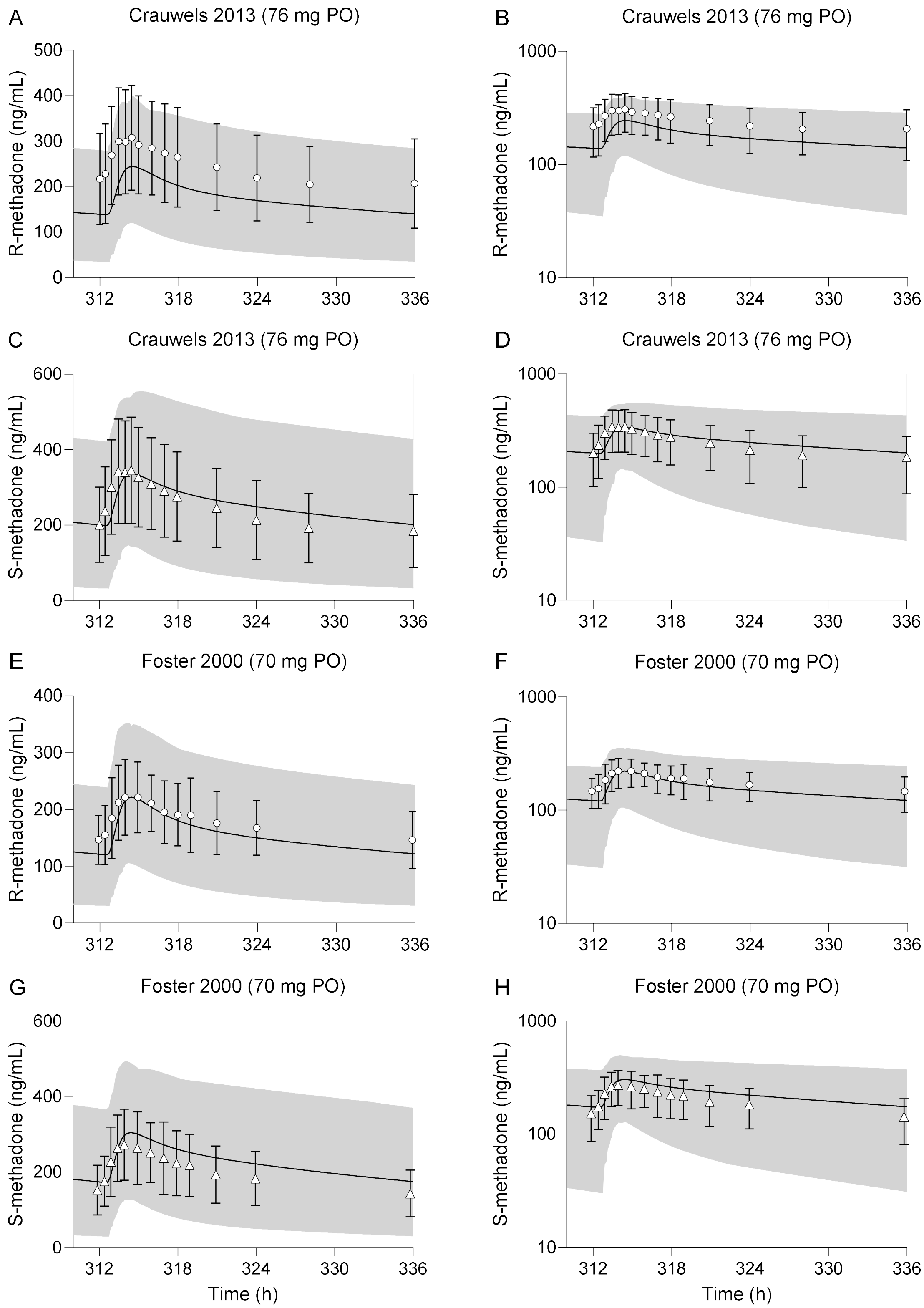

Figure A4. Visual predictive checks for methadone in adults (multidose, PO). The solid line is the predicted mean of the simulated population and the shaded area represents the 5th to 95th percentile of the virtual population. Symbols are observed datapoints (mean ± standard deviation), open circles are R-methadone concentrations and open triangles are S-methadone concentrations. (51, 52) On the right side (graphs B, D, F, and H) are semi-log plots of graphs A, C, E, and G, respectively. Abbreviations: PO: oral.

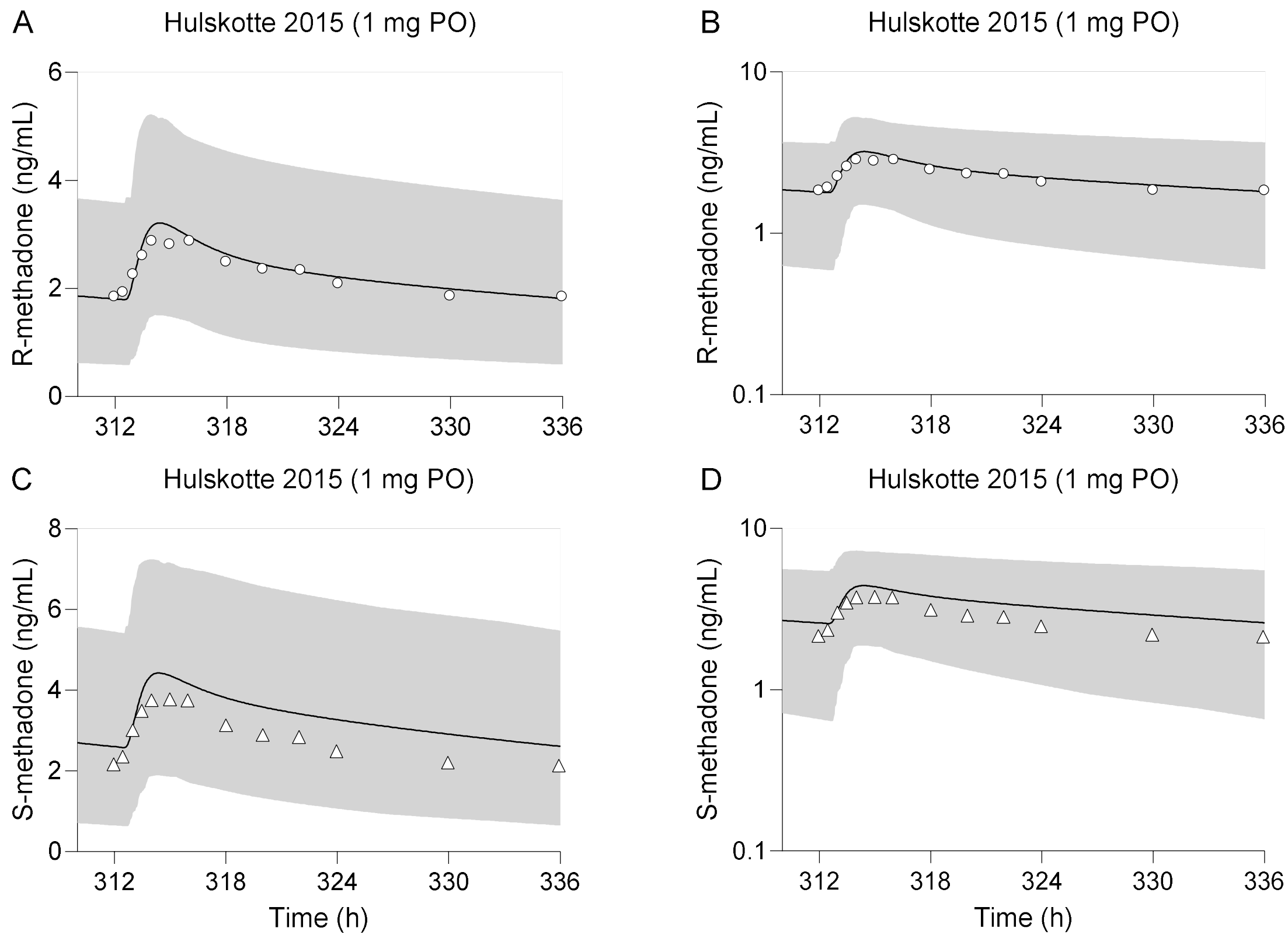

Figure A5. Visual predictive checks for methadone in adults (multidose, PO). The solid line is the predicted mean of the simulated population and the shaded area represents the 5th to 95th percentile of the virtual population. Symbols are mean observed datapoints, open circles are R-methadone concentrations and open triangles are S-methadone concentrations. (55) On the right side (graphs B and D) are semi-log plots of graphs A and C, respectively. Abbreviations: PO: oral.

Appendix 2. Model verification; methadone in pediatrics

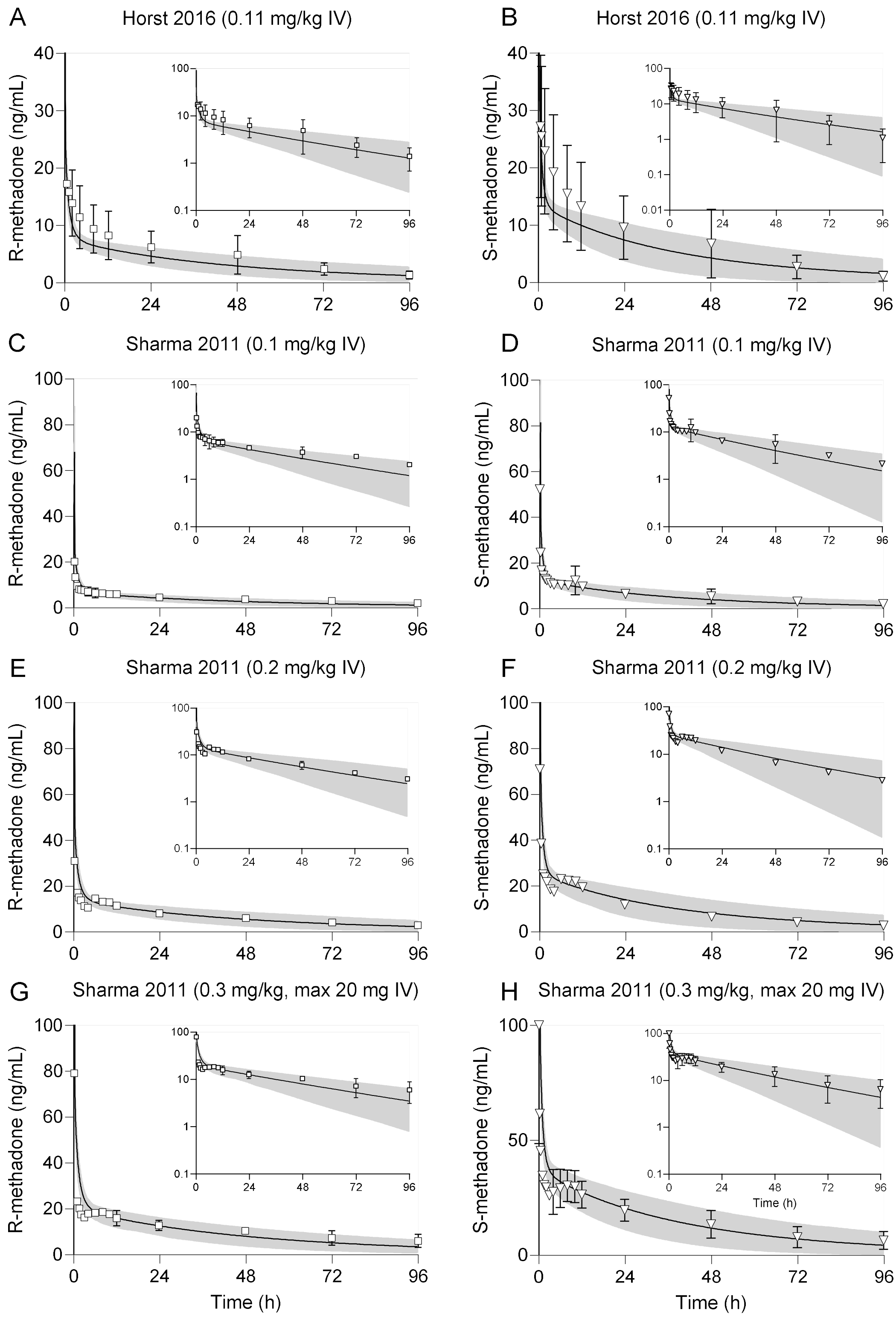

Figure A6. Visual predictive checks for methadone in pediatrics (single dose, IV). The solid line is the predicted mean of the simulated population and the shaded area represents the 5th to 95th percentile of the virtual population. Symbols are observed datapoints (mean ± standard deviation), inserts show semi-log plots. R-methadone concentrations (open squares) on the left (A, C, E, and G), S-methadone concentrations (open triangles up-side down) on the right (B, D, F, and H). Abbreviations: IV: intravenous.

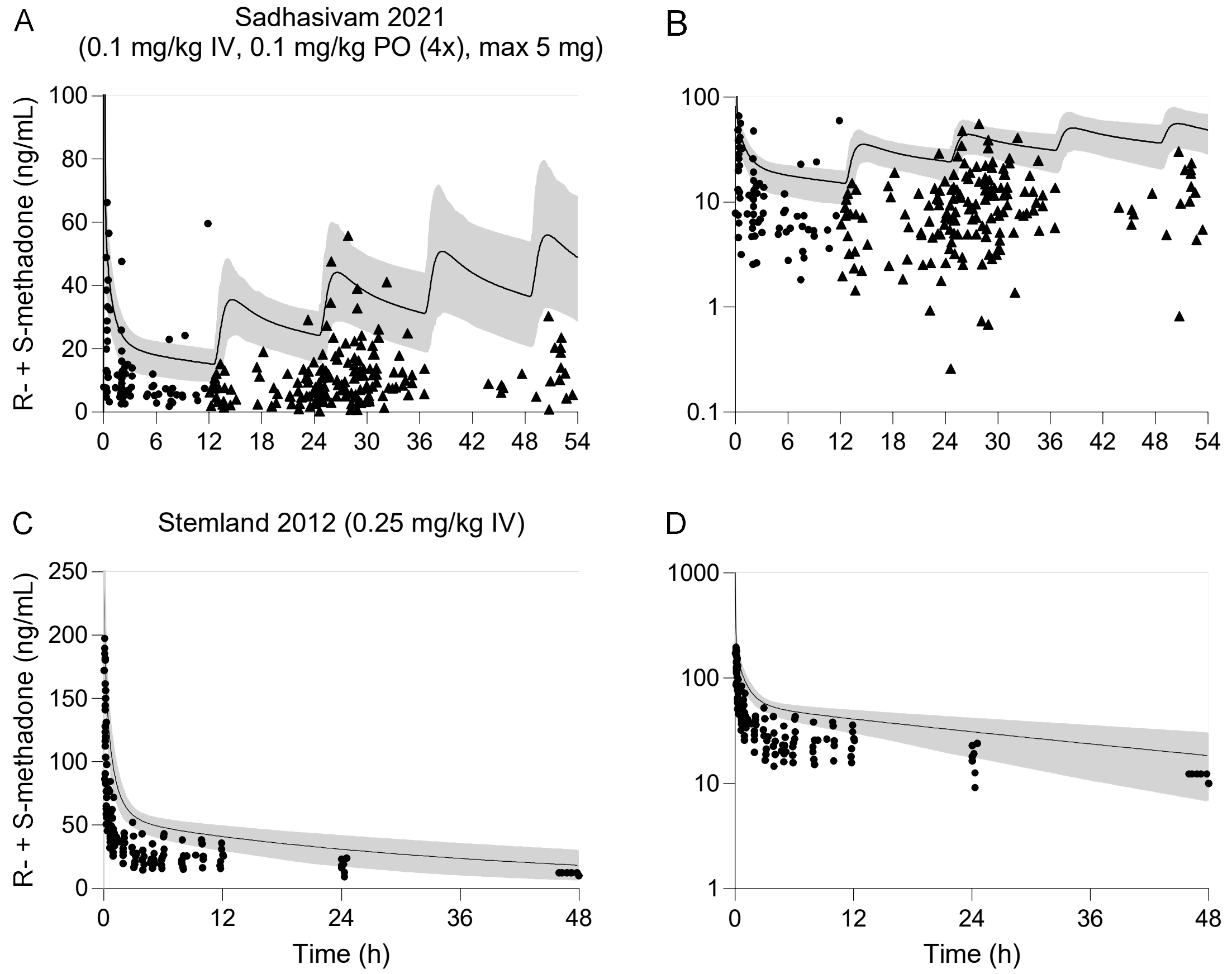

Figure A7. Visual predictive checks for methadone in pediatrics (single or multidose, PO and/or IV). The solid line is the predicted mean of the simulated population and the shaded area represents the 5th to 95th percentile of the virtual population. Symbols are individual observed datapoints of racemate methadone concentrations (36, 58). In the study by Sadhasivam et al., at first one IV dose is administered (solid circles), followed by four PO doses (solid triangles). In the study by Stemland et al., a single IV dose Is administered. On the right side (graphs B and D) are semi-log plots of graphs A and C, respectively. Abbreviations: IV: intravenous, PO: oral

Appendix 3. Model verification; methadone in neonates

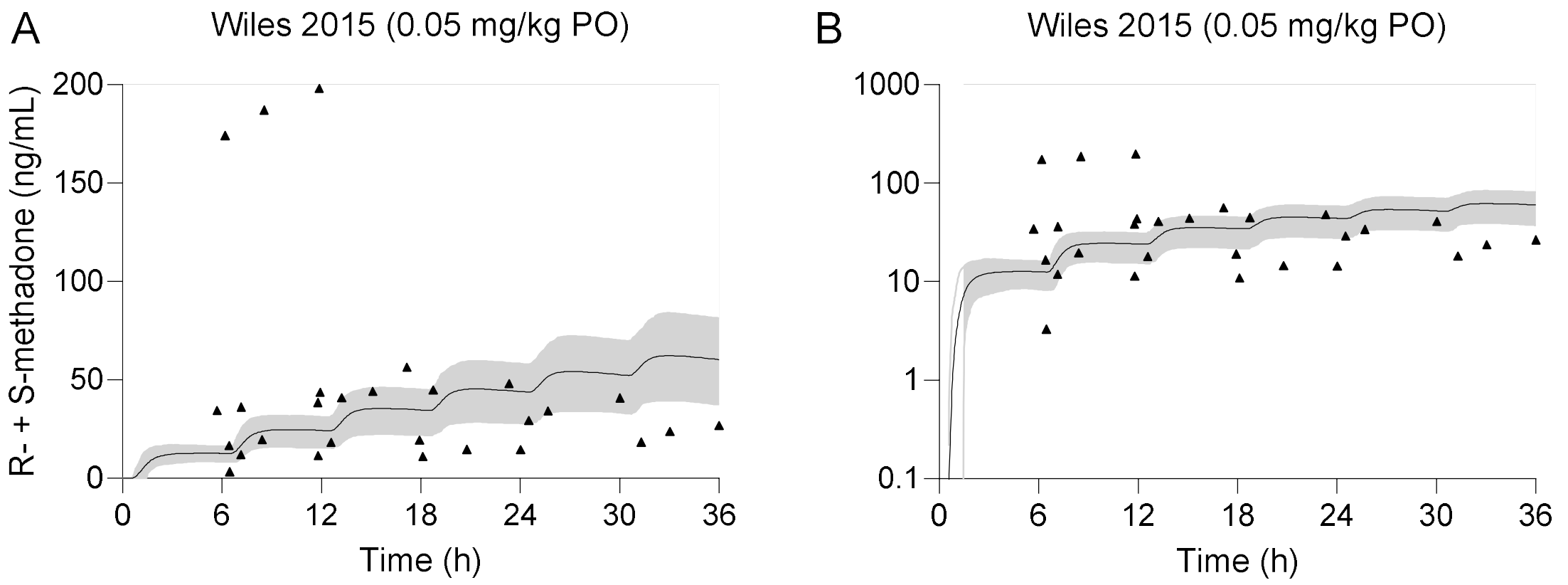

Figure A8. Visual predictive check for methadone in neonates (multidose, PO). The solid line is the predicted mean of the simulated population and the shaded area represents the 5th to 95th percentile of the virtual population. Symbols are individual observed datapoints of racemate methadone concentrations (33). Graph B is the semi-log plot of graph A. Abbreviation: PO: oral

Appendix 4. Model verification; fentanyl

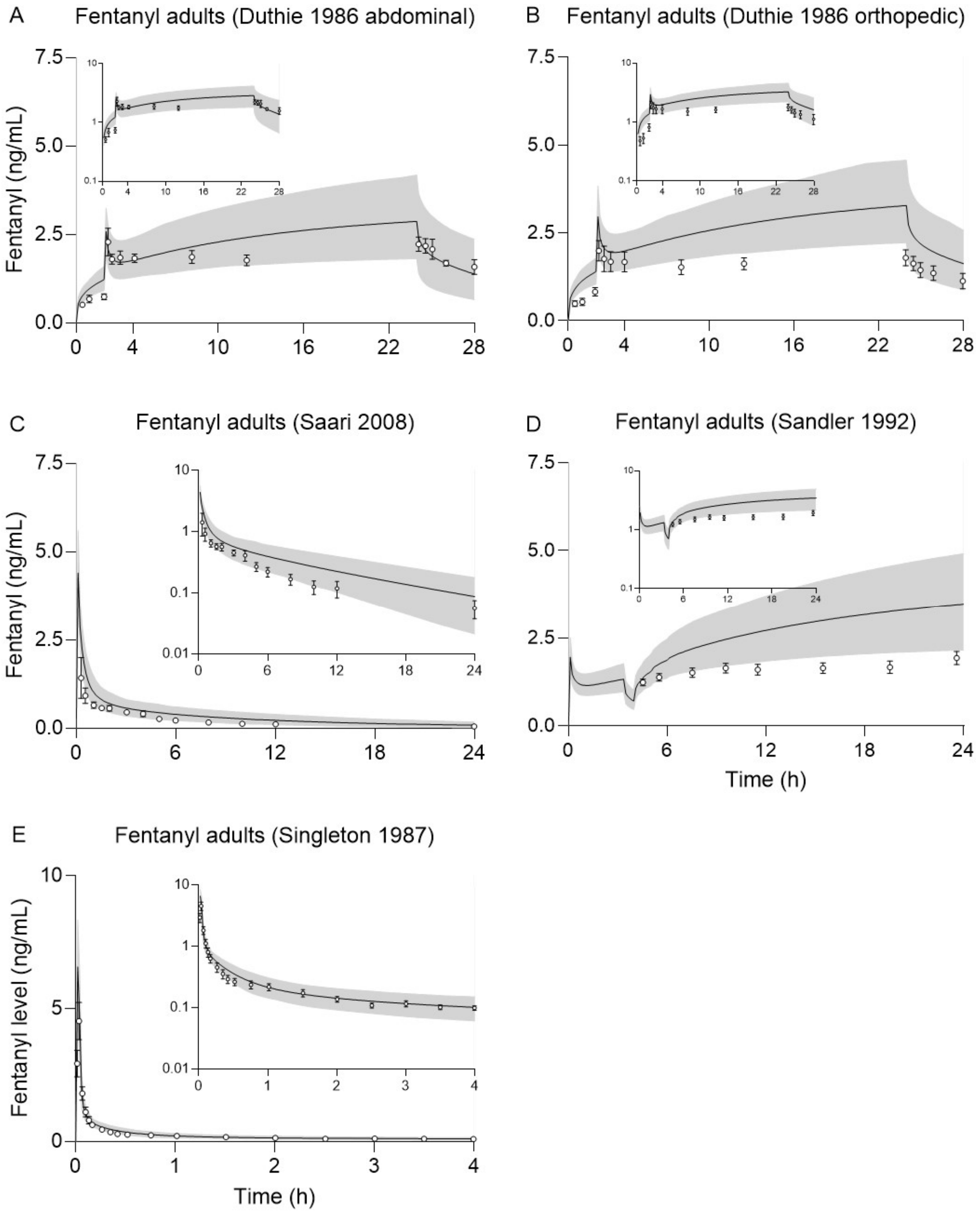

Figure A9. Visual predictive check for fentanyl in adults. The solid line is the predicted mean of the simulated population and the shaded area represents the 5th to 95th percentile of the virtual population. Symbols are observed datapoints (mean ± standard deviation), inserts show semi-log plots (41-44). The following doses were simulated: CI of 100 µg/h, after 2 hours an IV bolus of 100 µg (A, B), IV bolus of 5 µg/kg (C), a loading dose of 1.5 µg/kg, followed by varying infusions of 1-1.6 µg/kg/h (D), and an IV bolus of 1 µg/kg (E).

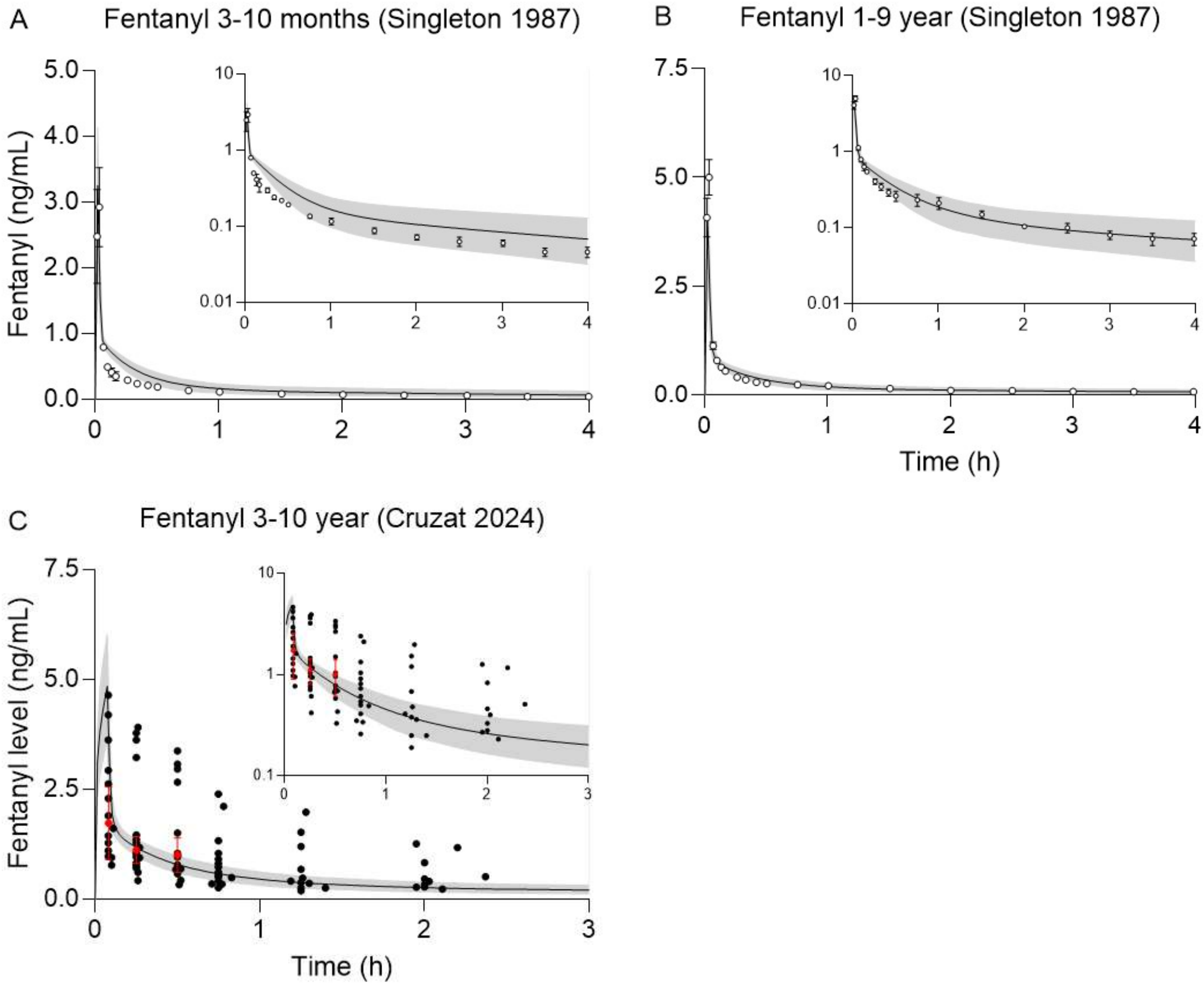

Figure A10. Visual predictive check for fentanyl in children. The solid line is the predicted mean of the simulated population and the shaded area represents the 5th to 95th percentile of the virtual population. Symbols are observed datapoints (mean ± standard deviation), inserts show semi-log plots (44, 45). The following IV doses were simulated: 1 µg/kg (A+B), 2 µg/kg (C).

Appendix 5. Model verification; morphine

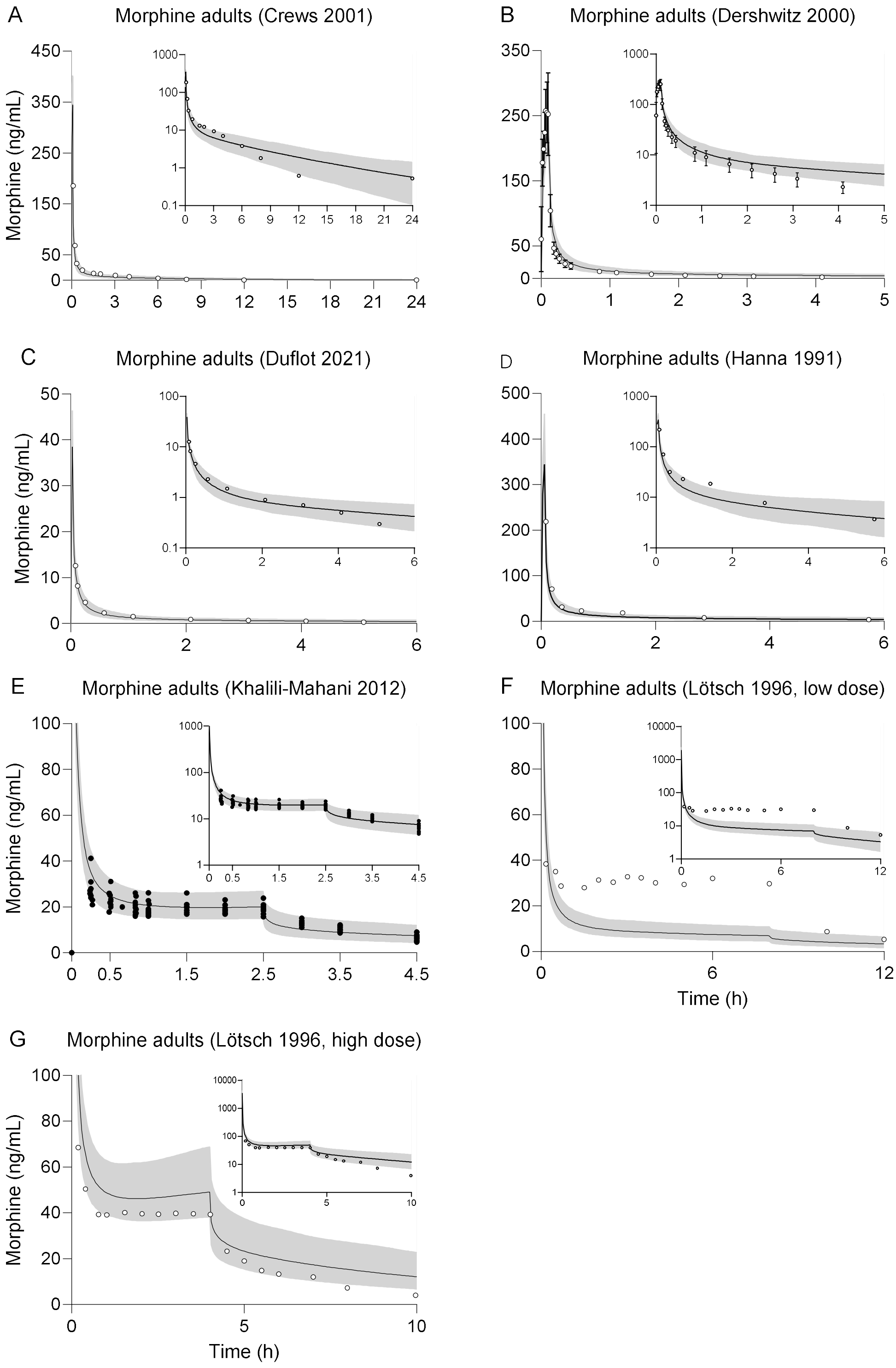

Figure A11. Visual predictive check for morphine in adults. The solid line is the predicted mean of the simulated population and the shaded area represents the 5th to 95th percentile of the virtual population. Open symbols are mean observed datapoints (± standard deviation), closed symbols are individual datapoints (62, 65, 66), inserts show semi-log plots. The following IV dosages were simulated: 10 mg (A), 8.8 mg (B), 1 mg (C), 120 µg/kg(D), 100 µg/kg bolus, followed by 30 µg/kg/h (E), 130 µg/kg bolus, followed by 5 µg/kg/h infusion (F), 240 µg/kg bolus, followed by 69 µg/kg/h infusion (G).

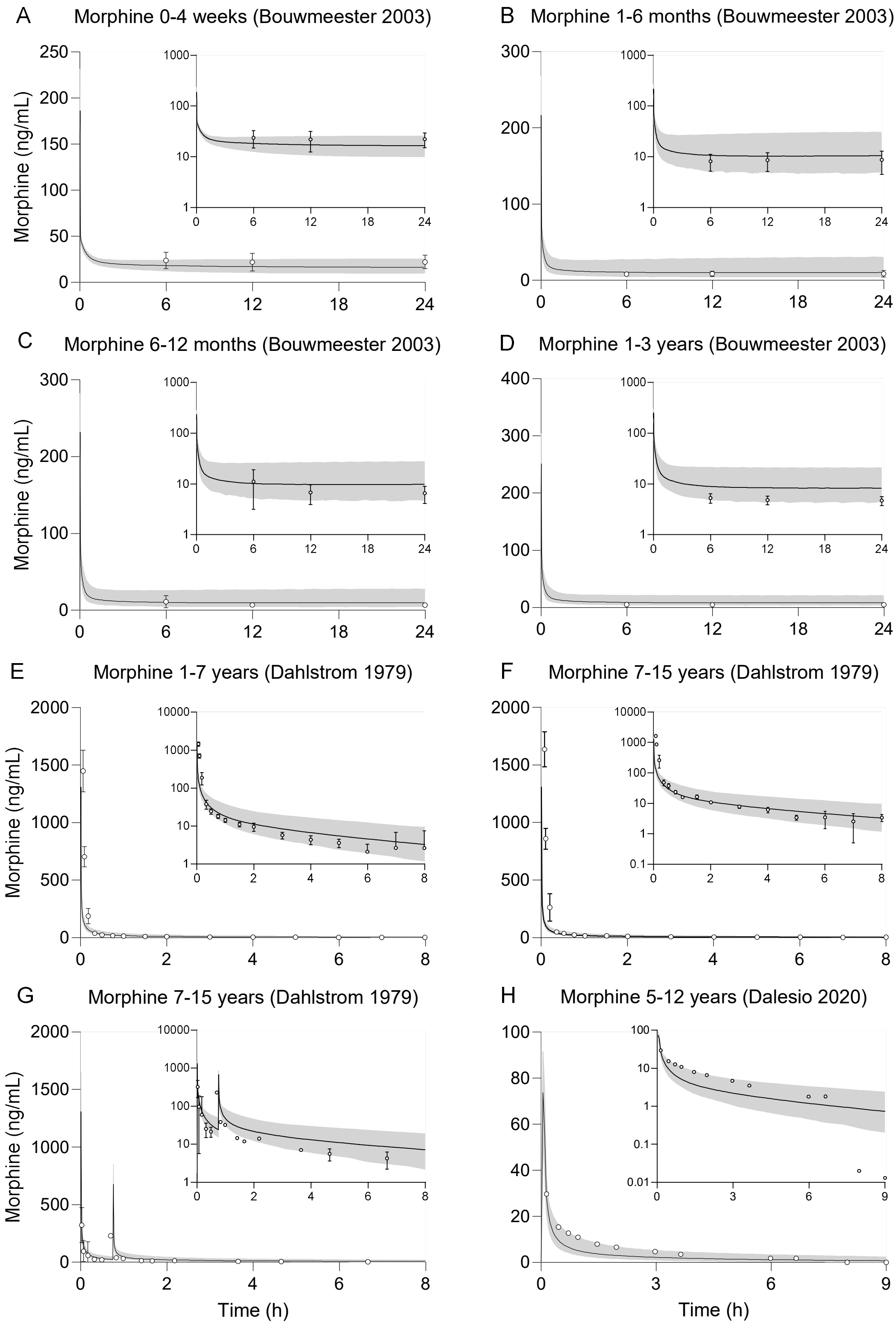
A Morphine 0-4 weeks (Bouwmeester 2003)
B Morphine 1-6 months (Bouwmeester 2003)
C Morphine 6-12 months (Bouwmeester 2003)
D Morphine 1-3 years (Bouwmeester 2003)
E Morphine 1-7 years (Dahlstrom 1979)
F Morphine 7-15 years (Dahlstrom 1979)
G Morphine 7-15 years (Dahlstrom 1979)
H Morphine 5-12 years (Dalesio 2020)
Morphine (ng/mL)
Time (h)

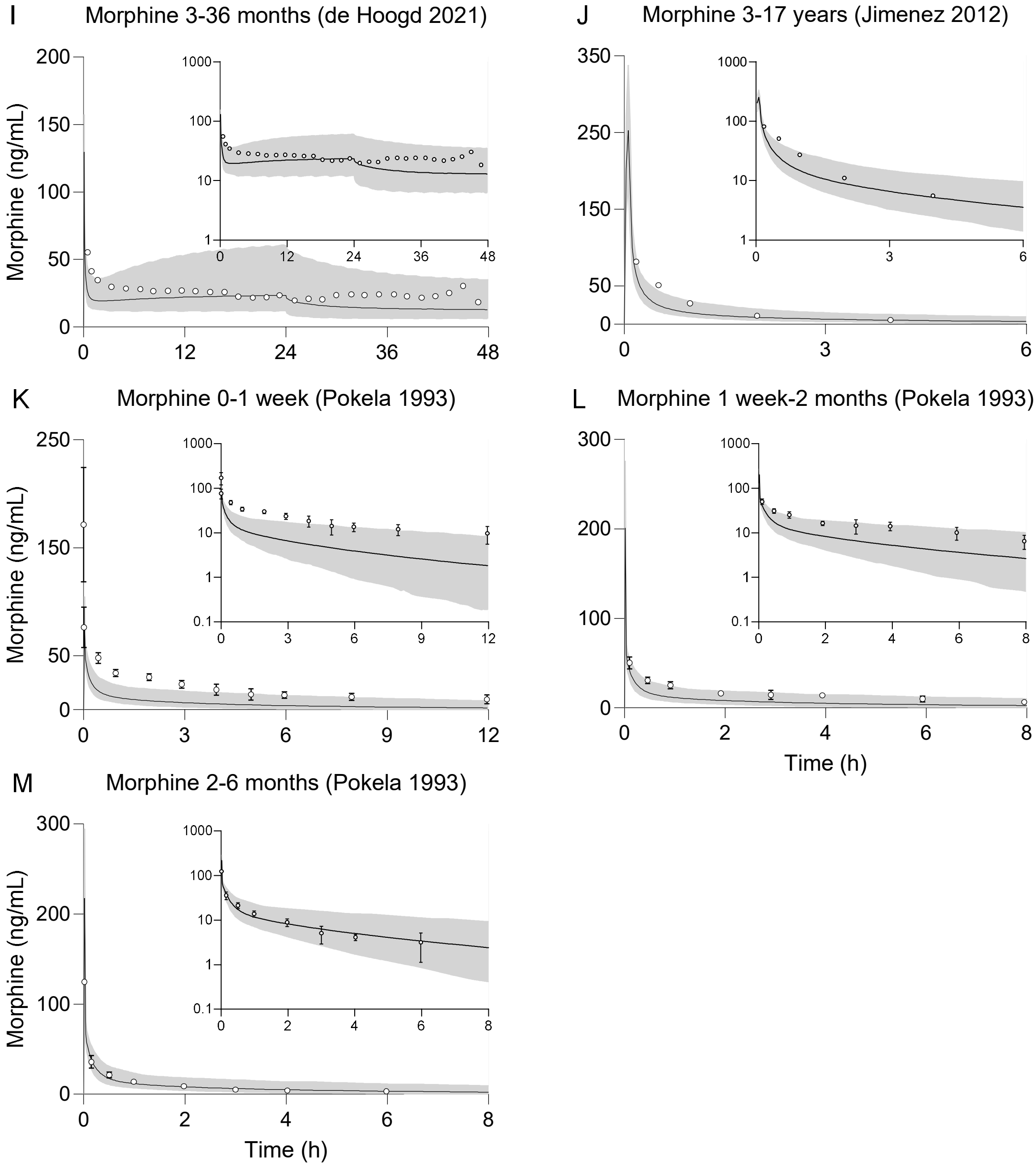

Figure A12. Visual predictive check for morphine in children. The solid line is the predicted mean of the simulated population and the shaded area represents the 5th to 95th percentile of the virtual population. Symbols are observed datapoints (mean ± standard deviation) (62, 65, 66), inserts show semi-log plots. The following IV dosages were simulated: 100 µg/kg bolus, followed by 10-12.3 µg/kg/h infusion (A-D), 0.15 mg/kg (E+F), 0.15 mg/kg + 0.075 mg/kg (G), 0.05 mg/kg (H), 0.1 mg/kg bolus, followed by a 24h infusion of 0.031 mg/kg/h, followed by a 24h infusion of 0.016 mg/kg/h (I), 0.15 mg/kg (J), 0.1 mg/kg (K-M).
